# Discovery of diversity in xylan biosynthetic genes by transcriptional profiling of a heteroxylan containing mucilaginous tissue

**DOI:** 10.3389/fpls.2013.00183

**Published:** 2013-06-07

**Authors:** Jacob K. Jensen, Nathan Johnson, Curtis G. Wilkerson

**Affiliations:** ^1^Department of Plant Biology, Michigan State UniversityEast Lansing, MI, USA; ^2^DOE Great Lakes Bioenergy Research Center, Michigan State UniversityEast Lansing, MI, USA; ^3^Department of Biochemistry and Molecular Biology, Michigan State UniversityEast Lansing, MI, USA

**Keywords:** xylan, psyllium, secondary cell wall, irx, glycosyltransferase, mucilage

## Abstract

The exact biochemical steps of xylan backbone synthesis remain elusive. In Arabidopsis, three non-redundant genes from two glycosyltransferase (GT) families, IRX9 and IRX14 from GT43 and IRX10 from GT47, are candidates for forming the xylan backbone. In other plants, evidence exists that different tissues express these three genes at widely different levels, which suggests that diversity in the makeup of the xylan synthase complex exists. Recently we have profiled the transcripts present in the developing mucilaginous tissue of psyllium (*Plantago ovata Forsk*). This tissue was found to have high expression levels of an IRX10 homolog, but very low levels of the two GT43 family members. This contrasts with recent wheat endosperm tissue profiling that found a relatively high abundance of the GT43 family members. We have performed an in-depth analysis of all GTs genes expressed in four developmental stages of the psyllium mucilagenous layer and in a single stage of the psyllium stem using RNA-Seq. This analysis revealed several IRX10 homologs, an expansion in GT61 (homologs of At3g18170/At3g18180), and several GTs from other GT families that are highly abundant and specifically expressed in the mucilaginous tissue. Our current hypothesis is that the four IRX10 genes present in the mucilagenous tissues have evolved to function without the GT43 genes. These four genes represent some of the most divergent IRX10 genes identified to date. Conversely, those present in the psyllium stem are very similar to those in other eudicots. This suggests these genes are under selective pressure, likely due to the synthesis of the various xylan structures present in mucilage that has a different biochemical role than that present in secondary walls. The numerous GT61 family members also show a wide sequence diversity and may be responsible for the larger number of side chain structures present in the psyllium mucilage.

## Introduction

A number of plants have seeds that produce mucilage that aids in hydration, dispersal and germination. The composition of mucilage varies considerably across species. As examples, *Arabidopsis thaliana* uses primarily pectin (Goto, 1985; Western et al., 2000) while flax utilizes a mixture of both pectin and arabinoxylan (Naran et al., 2008). Psyllium (*Plantago ovata* Forsk) mucilage is composed predominantly of complex heteroxylan (Edwards et al., 2003; Fischer et al., 2004; Guo et al., 2008) and, as such, presents an opportunity to discover genes involved in xylan production. The mucilage of psyllium is produced in a single cell tissue layer that is relatively easy to dissect from the developing seed. The mucilage produced by this tissue forms a large part of the tissue's dry mass and the ratio of xylan to cellulose is much higher than that found in secondary cell walls and thus represents an opportunity to distinguish genes involved in xylan formation from those involved in secondary cell wall biosynthesis. We have investigated this tissue, using transcriptional profiling, to determine which genes are highly expressed during mucilage formation. Using this approach we identified a previously uncharacterized component of the xylan synthases, IRX15 (Jensen et al., 2011).

Currently, a number of genes that affect xylan biosynthesis have been identified. In a few cases, the biochemical activities of these genes have been demonstrated; specifically, the addition of glucuronic acid side chain (GUX1, GUX2, GUX4; Lee et al., 2012a; Rennie et al., 2012) and the o-methylation of the glucuronic acid (GXMT1; Lee et al., 2012b; Urbanowicz et al., 2012). Three complementation groups of putative glycosyltransferase (GT) genes have been implicated in the synthesis of the β-(1,4)-linked xylose backbone of xylan. Each of these three complementation groups consist of two genes, one gene with secondary cell wall expression pattern, named *IREGULAR XYLEM (IRX) 9, IRX10* and *IRX14*, respectively, and one gene with much lower expression level and a more general expression pattern, named as their redundant homolog but with the suffix “*LIKE”* abbreviated *L*, e.g., *IRX9-L*. The four genes *IRX9(-L)* and *IRX14(-L)* are members of the GT family 43 (GT43) while the *IRX10(-L)*genes are members of the GT47 family (Brown et al., 2005, 2009; Persson et al., 2005; Peña et al., 2007; Wu et al., 2009, 2010; Lee et al., 2010). Our finding that IRX15, and its redundant homolog IRX15-L, also affects xylan chain length indicates further complexity of the xylan synthase (Brown et al., 2011; Jensen et al., 2011). Recently, a study performed in wheat endosperm has shown that, in contrast to Arabidopsis and psyllium, *IRX15* is not expressed at high levels in the endosperm tissue, but homologs of *IRX9, IRX14* and *IRX10* are highly expressed (Pellny et al., 2012). This result indicates that variation is possible in the makeup of the xylan synthase. It would appear that the synthesis of xylan in wheat endosperm does not require IRX15. Our previous results demonstrate that the xylan synthase responsible for complex heteroxylan biosynthesis in psyllium does not require IRX9 or IRX14, as these were found to be expressed at very low levels in this tissue. A homolog of IRX10 was, on the other hand, found to be abundantly expressed (Jensen et al., 2011). These indications of diversity in the xylan synthase seem to suggest that the one constant in xylan synthesis is IRX10. If IRX10 is primarily responsible for the synthesis of the xylan backbone it would be expected that the xylan synthase from the psyllium mucilaginous layer (ML) would express an IRX10 gene with different properties than found in tissues containing both GT47 and GT43 family members. Additionally, one would expect to find GTs responsible for the larger variety of xylan side chains found in the psyllium mucilage. We present in this study an examination of the *IRX10* genes present in the ML, as well as stem tissue, and we examine other highly abundant transcripts in the ML encoding proteins likely involved in xylan biosynthesis.

## Materials and methods

### Plant growth, cell wall analysis, and RNA-SEQ

Psyllium (Indian, Plantago ovata, Sand Mountain Herbs, AL, USA) and Arabidopsis (*Col-0*) plants were grown as previously described (Jensen et al., 2011).

Toluidine blue staining of psyllium inflorescence, stem top half and stem bottom half was performed on free-hand sections of fresh material. Sequential extraction of cell wall material from leaves, inflorescence, stem top half and stem bottom half and subsequent neutral monosaccharide analysis of the 1 M KOH fraction was performed as described in Jensen et al. (2011).

Whole stems from 3-month-old psyllium plants were used for the preparation of total RNA extraction using Trizol reagent (15596-026; Invitrogen, http://www.invitrogen.com/). Of the crude RNA preparation 20 μg was subjected to additional purification using the RNeasy Micro Kit (74004; Qiagen, http://www.qiagen.com/) with DNase treatment (79254; Qiagen, http://www.qiagen.com/) as per manufacture's protocol. The subsequent cDNA library and high-throughput cDNA sequencing (RNA-Seq) was performed as described in Jensen et al. (2011). The RNA-Seq datasets were deposited at NCBI Sequence Read Archive (http://www.ncbi.nlm.nih.gov/sra) with the following accessions: 6 DPA, SRX096079; 8 DPA, SRX027102; 10 DPA, SRX096080; 12 DPA, SRX027103; stems 10 weeks, SRX027101.

### Assembly of 454 ESTs and database construction

The five datasets of 454 ESTs were assembled collectively using the CLC Genomics Workbench version 4.7.2 (CLC bio, Cambridge, MA, USA) and the De-novo assembly algorithm (Parameters: Similarity 0.8; Length fraction 0.5; Insertion cost 3; Deletion cost 3; Mismatch cost 2). Unique counts were generated by aligning ESTs to the assembled contigs using the RNA-Seq Analysis algorithm for non-annotated sequences. (Parameters: Similarity 0.8; Length fraction 0.9). The assembled sequence contigs were annotated using TBLASTN (Altschul et al., 1997) against the TAIR 9 annotation of the Arabidopsis genome. The annotations were subsequently expanded with the following information: Arabidopsis gene family assignments from the Carbohydrate Active enZyme (CAZy) database (Cantarel et al., 2009; http://www.cazy.org; update 2012-05-31) were labeled e.g., “Glycosyltransferase Family 47″ or “Glycoside Hydrolase Family 19″; Arabidopsis proteins not included in CAZy but recently proposed to also encode GTs (Nikolovski et al., 2012) were labeled GT and the respective family name, eg. “Glycosyltransferase Family GT14R”; members of the nucleotide sugar transporter/triose phosphate translocators family in Arabidopsis (Ward, 2001) were added the label “NST/TPT family”; and transcription factors in the Database of Arabidopsis Transcription Factors (DATF; Guo et al., 2005; http://datf.cbi.pku.edu.cn/) were added the label “Transcription Factor”; genes co-expressed with IRX10 (*r* > 0.5; 184 genes) and with secondary cell wall CESA4, CESA7 and CESA8 (*r* > 0.5; 227 genes) (GeneCAT database; http://genecat.mpg.de/cgi-bin/Ainitiator.py; Mutwil et al., 2008) were added the label “AtIRX10 Co-expression” and “At SCW CESA Co-expression,” respectively. Contig name, DNA sequence, annotation and expression information were stored in an Oracle relational database that is located at http://glbrc.bch.msu.edu/psyllium. The database can be queried using keywords that search contig annotation, including the added annotations mentioned above, while the contig sequence information can be analyzed using BLAST (Altschul et al., 1997) and query sequences, either DNA or protein, provided by the user. Information about each contig, such as DNA sequence, EST coverage and BLAST report against TAIR9, can be retrieved by clicking on the contig ID numbers and the “T” icon associated with each contig. Access to the individual contig data facilitates manual analysis for artifact assembly, such as ESTs from different genes grouped into the same contig or the identification of multiple contigs originating from the same transcript. Finally, a micro array viewer based on a gene expression map of Arabidopsis development (Schmid et al., 2005) is provided for each contig by clicking on the associated AGI.

### Identifying genes of interest

Because of sequencing errors, ESTs from one gene were in some cases assembled into two or more individual contigs. In the cases of *PoIRX10_1* to *_4* and *PoGT61_1* to *_7* the complete cDNA sequences were determined by cDNA cloning and Sanger sequencing. Four independent clones were sequenced in each case. *PoIRX10_2* is not full length. The verified cDNA sequences were deposited at NCBI GenBank (http://www.ncbi.nlm.nih.gov/genbank) with the following accessions KC832826 to KC832829 (PoIRX10_1 to _4) and KC894060 to KC894066 (PoGT61_1 to _7).

### Phylogenetic analysis

Phylogenetic trees were calculated by the use of MEGA 5.05 (Tamura et al., 2011), using the built-in ClustalW (Larkin et al., 2007) sequence alignment program, the Maximum Likelihood algorithm (Nei and Kumar, 2000), using the Poisson substitution model and bootstrapping based on 500 trees (Felsenstein, 1985). The phylogenetic analysis of GT61 members was based on protein sequences only. The phylogenetic analysis of GT47 members was based on cDNA sequences. First cDNA sequences were loaded in the MEGA program, then translated into protein sequences and aligned using the built-in ClustalW function (File S2; Larkin et al., 2007). The resulting codon based cDNA alignment was then used for phylogenetic analysis. Codon positions included were first, second, third, and non-coding.

Protein sequences were obtained from the Phytozome v8.0 database (Goodstein et al., 2012; http://www.phytozome.net/). For poplar (*Populus trichocarpa*, annotation v3.0) the genes Potri015G107200 and Potri015G116700 were not included in the analysis as these represent partial sequences. GT family 61 proteins from Arabidopsis and rice (*Oryza sativa* Japonica Group) were obtained from the CAZy database. In *Brachypodium distachyon*, all proteins annotated as GT family 61 proteins based on the recent genome annotation (International Brachypodium Initiative, 2010) were included.

### Determining degree of cell wall acetylation

Ground plant material of Arabidopsis lower stem, dissected mucilaginous layers (8–10 DPA), psyllium husk (Now Foods, www.nowfoods.com), and whole psyllium seeds were washed three times with 70% ethanol, three times with 1:1 methanol-chloroform, and two times with acetone to obtain alcohol insoluble residue (AIR). Acetyl groups from the alcohol insoluble residue were then released by alkaline hydrolysis by treating with 1 M KOH at room temperature for 5 min and then neutralized with an equal amount of HCl. The amount of freed acetic acid in solution was then subsequently determined using the K-ACETRM acetic acid quantification kit from Megazyme (www.megazyme.com).

## Results and discussion

### Transcript profiling of psyllium stem tissue, assembly of ESTs and assignment of functional annotation

**Figure 1:**
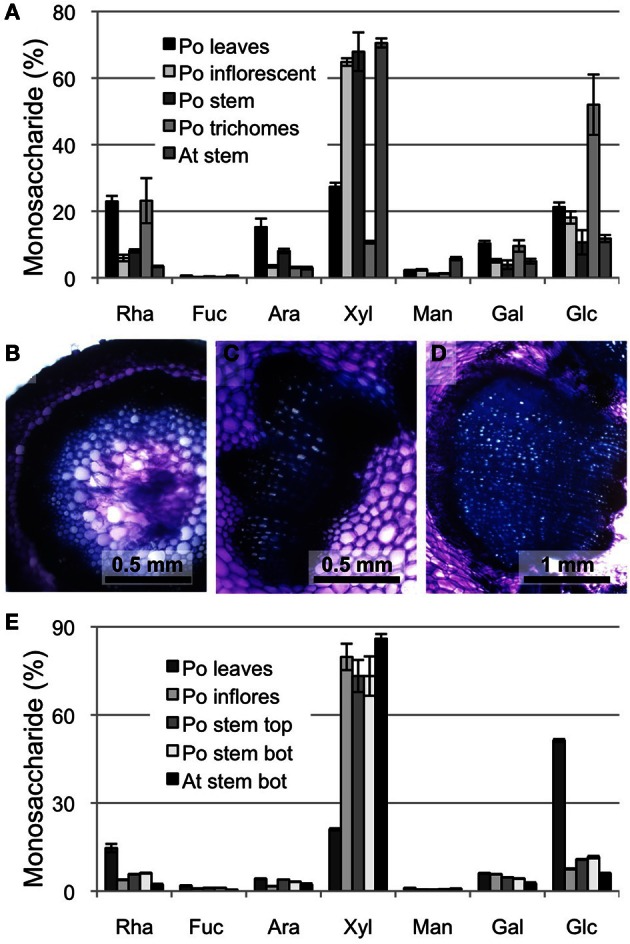
**Cell wall analysis of psyllium aerial tissues. (A)** Neutral monosaccharide composition of total cell walls from various tissues from psyllium (Po) leaves, inflorescent, stem and trichomes and Arabidopsis (At) stem. **(B–D)** Toluidine blue staining of free-hand sections of psyllium inflorescence **(B)**, stem top **(C)** and stem bottom **(D)**. **(E)** Neutral monosaccharide composition of 1 M KOH extractions of various tissues from psyllium leaves, inflorescent, stem top and stem bottom, and Arabidopsis stems bottom. The selected tissues were subjected to sequential extractions with CDTA, Na_2_CO_3_ and 1 M KOH.

In order to compare xylan biosynthesis in the ML with xylan formation in other tissues of psyllium we first determined the neutral monosaccharide composition for different aerial parts of the plant (Figure 1A). The psyllium stem and inflorescence yielded the highest levels of xylose, which were at levels comparable to Arabidopsis stem. Given glucose levels are low in these tissues, the high levels of xylose likely result from xylan as opposed to xyloglucan. Anatomical investigation by hand sectioning and toluidine blue staining verified the presence of secondary cell wall formation in both inflorescence and stem (Figures 1B–D). Subsequently, a series of sequential extractions, using CDTA, Na_2_CO_3_ and KOH, were performed and the xylan enriched 1 M KOH fraction was subjected to neutral monosaccharide composition analysis (Figure 1E). Only minor differences were found in the monosaccharide profiles between Arabidopsis lower stem, psyllium inflorescence and psyllium stem samples. Based on these analyses we chose to profile the transcriptome of psyllium stem.

The sequence data from the psyllium stem RNA-Seq experiment was added to four previous RNA-Seq datasets from psyllium ML (Jensen et al., 2011). This dataset of approximately 1 million ESTs was assembled into transcript models (contigs; Table S1 in Supplementary Material), annotated and stored in an Oracle relational database that is located at http://glbrc.bch.msu.edu/psyllium.

### Overview of glycosyltransferases highly expressed in psyllium mucilaginous layers

Assembly and annotation of the five RNA-Seq datasets from psyllium resulted in identification of 634 contigs encoding putative GTs. The top 50 transcripts from this set are listed in Table 1 ranked by expression in the ML at 10 days post anthesis (DPA) stage. The most abundant transcripts encoding putative GTs (1000 ppm or higher in at least one of the four ML stages) are homologs of IRX10(-L) (GT47), GUX5 (GT8; Mortimer et al., 2010), RGP1/UAM (GT75; Konishi et al., 2007), and AT3G18170/AT3G18180 (GT61), and are likely involved in complex heteroxylan biosynthesis. Most of these highly abundant ML transcripts are not found in the stem transcriptome (Table 1). Multiple homologous genes related to AT3G18170/AT3G18180 and IRX10(-L) are present in psyllium. These two gene families were investigated in further detail.

**Table 1:** **The 50 most abundant transcripts expressed in psyllium mucilaginous layers encoding putative glycosyltransferases**.

**Contig**	**AGI**	**Gene name**	**GT family**	**6 DPA^a^^,^^b^**	**8 DPA**	**10 DPA^c^**	**12 DPA**	**Stem**
M01000012733	AT5G61840	*IRX10L*	CAZy GT47	104	508	4919	8557	0
M01000017653	AT3G18180		CAZy GT61	2958	3787	3620	1093	0
M01000032237	AT3G02230	*RGP1, UAM*	CAZy GT75	5063	7559	3561	4548	142
M01000025200	AT3G18170		CAZy GT61	2001	3189	3433	926	0
M01000012668	AT3G18180		CAZy GT61	153	762	3178	2195	0
M01000021834	AT3G18170		CAZy GT61	153	541	1889	2009	8
M01000033105	AT4G32290		GT14R	509	590	1702	2828	0
M01000025204	AT3G18170		CAZy GT61	1694	2058	1702	647	0
M01000017654	AT3G18180		CAZy GT61	1479	1361	1672	304	2
M01000025153	AT2G32750		CAZy GT47	18	180	1200	588	0
M01000026523	AT3G18170		CAZy GT61	6	197	1092	1759	0
M01000007355	AT1G54940	*GUX5*	CAZy GT8	350	467	1072	314	0
M01000007434	AT5G05170	*CESA3*	CAZy GT2	270	377	944	221	1217
M01000021804	AT4G32410	*CESA1*	CAZy GT2	288	410	817	475	1125
M01000031196	AT5G44820		CAZy GT77	6	25	797	828	0
M01000007257	AT1G27440	*IRX10*	CAZy GT47	423	279	639	255	14
M01000007407	AT5G22740	*CSLA2*	CAZy GT2	141	385	630	299	350
M01000026539	AT2G21770	*CESA9*	CAZy GT2	246	205	610	270	289
M01000022490	AT3G18180		CAZy GT61	865	697	600	284	0
M01000008210	AT4G37690	*GMGT*	CAZy GT34	331	295	580	289	61
M01000007300	AT3G18170		CAZy GT61	203	303	580	240	0
M01000025226	AT5G12460		CAZy GT31	98	107	551	750	0
M01000031203	AT4G38040		CAZy GT47	288	254	521	162	0
M01000007383	AT5G15650	*RGP2*	CAZy GT75	460	336	512	490	191
M01000021884	AT1G22380	*UGT85A3*	CAZy GT1	190	221	482	417	4
M01000025271	AT1G51630	*MSR2*	GT65R	246	344	413	191	246
M01000025210	AT4G18780	*CESA8, IRX1*	CAZy GT2	92	82	384	196	43
M01000032248	AT3G11420		CAZy GT31	215	254	374	299	12
M01000031122	AT3G18170		CAZy GT61	147	221	334	132	0
M01000007513	AT5G62220	*XLT2*	CAZy GT47	31	66	334	235	10
M01000011893	AT5G07720	*XXT3*	CAZy GT34	92	115	334	29	41
M01000022396	AT5G61840	*IRX10L*	CAZy GT47	601	664	325	54	0
M01000013504	AT5G12460		CAZy GT31	184	148	315	240	0
M01000029398	AT1G76270		GT65R	86	164	285	64	4
M01000031329	AT1G08280		CAZy GT29	350	394	285	221	0
M01000031118	AT4G08810	*SUB1*	GT68R-A	129	41	275	29	140
M01000031298	AT3G04240		CAZy GT41	227	221	266	137	468
M01000011952	AT4G18780	*CESA8, IRX1*	CAZy GT2	166	131	266	191	57
M01000025335	AT5G64740	*CESA6*	CAZy GT2	80	139	246	59	458
M01000012928	AT3G18170		CAZy GT61	252	361	246	88	0
M01000031220	AT3G29320		CAZy GT35	141	90	236	83	45
M01000012711	AT1G67850		GT27R	98	115	177	216	4
M01000007517	AT5G44030	*CESA4, IRX5*	CAZy GT2	203	139	177	20	151
M01000014584	AT2G37980		GT65R	12	25	157	59	4
M01000017066	AT3G18180		CAZy GT61	325	631	157	34	0
M01000025502	AT3G28180	*CSLC04*	CAZy GT2	37	90	157	20	122
M01000007773	AT3G11340		CAZy GT1	18	49	157	39	26
M01000007351	AT5G13000	*GSL12*	CAZy GT48	86	148	148	83	161
M01000025159	AT2G45830		CAZy GT90	49	25	148	250	0
M01000017747	AT3G25140	*GAUT8, QUA1*	CAZy GT8	123	107	148	44	22

a Days post anthesis, DPA.

b Expression data is in parts per million (ppm).

c Transcripts are ranked by expression in the mucilaginous layers at the 10 DPA stage.

A significant level of primary cell wall biosynthesis is evident in the ML. Homologs of CESA1 and CESA3 (Arioli et al., 1998; Desprez et al., 2007; Persson et al., 2007) are found expressed in the range of 200 to 1000 ppm, while expression of putative xyloglucan GTs are found in the range of 50 to 350 ppm; e.g., homologs of CSLC4 (Cocuron et al., 2007), XLT2 (Jensen et al., 2012) and XXT3 (Vuttipongchaikij et al., 2012) (Table 1). A homolog of GAUT1 (Sterling et al., 2006) is found to be expressed at 79 ppm at 10 DPA, providing evidence for homogalacturonan synthesis. A homolog of the callose synthase, GSL12, is most abundant at 8 to 10 DAP (148 ppm) in the ML, indicating that cell division is taking place (Chen et al., 2009). Some level of secondary cell wall biosynthesis also appears to be present. Transcripts with homology to secondary cell wall CESA8 (IRX1) and CESA4 (IRX5) (Turner and Somerville, 1997; Persson et al., 2005) are found at a similar abundance as the GTs involved in xyloglucan biosynthesis. Transcripts with homology to CESA2, CESA5 and CESA9 are present in the ML transcriptome, especially abundant are transcripts with homology to CESA9. These three CESA proteins have been found to play important roles in Arabidopsis seed coat development, namely in mucilage attachment (CESA5) and formation of a secondary cell wall that reinforces the columella and radial wall (Mendu et al., 2011).

Evidence of mannan biosynthesis is indicated by the presence of CSLA2 (Dhugga et al., 2004; Goubet et al., 2009), MSR2 (Wang et al., 2012) and galactomannan galactosyltransferase (GMGT) (Edwards et al., 1999) homologs that have expression levels as high as 630 ppm (CSLA2 homolog, 10 DPA; Table 1). This finding is likely a result of endosperm tissue contamination in the dissected ML. The endosperm stores large amounts of mannan (Jensen et al., 2011) and given the attachment of the endosperm to the ML it is difficult to obtain ML tissue completely devoid of endosperm.

Out of the 50 most abundant transcripts shown in Table 1 there are 14 putative GT transcripts that cannot readily be assigned a function or to a pathway. Notably, many of these abundant transcripts have no expression in the stem transcriptome, as is seen for transcripts likely involved in heteroxylan biosynthesis (GT8, GT47, GT61, and GT75). This is in contrast to GTs involved in primary and secondary cell wall biosynthesis which reach expression levels in the stem of approximately 50 ppm or higher. The ML specific GTs without an assigned function therefore represent GTs possibly involved in complex heteroxylan synthesis in the psyllium ML, though involvement in other pathways unrelated to xylan syntheis is also possible.

### Psyllium stem xylan biosynthesis is similar to Arabidopsis

All the transcripts identified encoding proteins homologous to IRX9(-L), IRX10(-L), IRX14(-L) and IRX15(-L) are listed in Table 2. This group of transcripts, with the exception of some IRX10(-L) and IRX15(-L) transcripts, had low expression or were not found in the ML. In the stem, the expression of these xylan specific genes was found to be unexpectedly low (100 ppm or lower). It appears, however, that this tissue is principally engaged in primary rather than secondary cell wall biosynthesis. When examining the expression of both the primary and secondary cell wall CESAs in the stem, the primary CESAs were found at levels as high as 1217 ppm (CESA3; Table 1) while the secondary CESAs were found at 10 fold lower levels. The expression of IRX9(-L), IRX10(-L), IRX14(-L), and IRX15(-L) in the stem therefore matches the level of secondary cell wall formation in this tissue. Therefore, it appears that psyllium has a similar complement of GTs found to be responsible for xylan synthesis as in Arabidopsis and that these genes are expressed at comparable levels in the psyllium stem.

**Table 2:** **All transcripts from psyllium stem and mucilaginous layers encoding proteins homologous to Arabidopsis IRX9(-L), IRX10(-L), IRX14(-L), and IRX15(-L)**.

**Gene**	**Contig**	**6 DPA^a^^,^^b^**	**8 DPA**	**10 DPA^c^**	**12 DPA**	**Stem**
***IRX9(-L)***						
AT1G27600	M01000026144	12	8	10	0	33
AT1G27600	M01000031822	6	8	10	0	28
AT2G37090	M01000017727	0	25	0	15	4
AT2G37090	M01000026536	0	8	10	0	4
***IRX10(-L)***						
AT5G61840	M01000012733	104	508	4919	8557	0
AT1G27440	M01000007257	423	279	639	255	14
AT5G61840	M01000022396	601	664	325	54	0
AT5G61840	M01000012809	325	156	128	25	0
AT5G61840	M01000013318	190	98	79	5	2
AT5G61840	M01000026636	117	16	118	34	8
AT5G61840	M01000010529	6	16	10	0	63
AT1G27440	M01000011294	0	0	0	0	41
AT1G27440	M01000004742	0	0	0	0	8
***IRX14(-L)***						
AT5G67230	M01000007747	68	90	69	59	108
***IRX15(-L)***						
AT5G67210	M01000007937	1178	926	994	887	10
AT5G67210	M01000025441	6	0	20	0	8
AT5G67210	M01000030764	0	0	0	0	8
AT3G50220	M01000004819	0	0	0	0	4

a Days post anthesis, DPA.

b Expression data is in parts per million (ppm).

c Transcripts are ranked by expression in the mucilaginous layers at the 10 DPA stage.

### Four homologs of Arabidopsis IRX10 are highly expressed in psyllium mucilaginous layers

Transcripts encoding proteins homologous to IRX10(-L) show tissue specific distributions (Table 2), with transcripts present at high levels in the ML showing little or no expression in the stem, and *vice versa*. The presence of these two categories of IRX10(-L) transcripts led us to consider that at least two different genes with homology to *IRX10(-L)* are present in psyllium. We therefore manually examined a total of 12 IRX10(-L) contigs and found evidence of six unique IRX10(-L) genes in psyllium, named *Plantago ovata IRX10 1* to *6* (*PoIRX10_1* to _*6*). Four of these, those showing abundant expression in the ML (*PoIRX10_1* to *_4*), were cloned from cDNA and sequenced. Analysis of the deduced amino acid sequence of *PoIRX10_1, PoIRX10_3*, and *PoIRX10_4* for transmembrane domains as predicted by the TMHMM Server v. 2.0 (Krogh et al., 2001; http://www.cbs.dtu.dk/services/TMHMM/) resulted in a high score for a single N-terminal transmembrane domain for PoIRX10_1, an intermediate score for PoIRX10_4, and a very low score for PoIRX10_3 (File S1). The *PoIRX10_2* cDNA sequence is missing the 5' end and was not analyzed.

**Figure 2:**
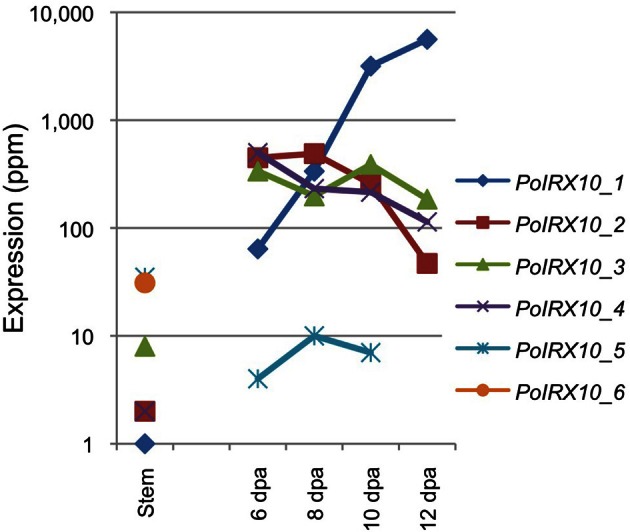
**Expression levels of IRX10 homologs in psyllium in stem and mucilaginous layers**.

The expression of *PoIRX10_1* to *_6* is shown in Figure 2. The expression profiles for *PoIRX10_1* to *_4* were generated by mapping the RNA-Seq data to the sequences obtained from the cDNA clones. The expression profile for *PoIRX10_1* shows strong induction in the ML and reached maximum levels at 12 DPA, while *PoIRX10_2* to *_4* show a flat or a decreasing expression pattern over the four ML stages. *PoIRX10_6* is not detected in the ML but is present in stem together with *PoIRX10_5*. The *PoIRX10_5* is found in the ML but at a 10 fold lower level than *PoIRX10_1 to *_4*.*

### PoIRX10_1, _2 and _4 represent some of the most divergent IRX10 proteins yet identified

An examination of homologs of IRX10 from various higher plants showed a high degree of sequence conservation among these proteins. To obtain a broader view of this, we collected all IRX10 homologs from six different plant species with extensive phylogenetic diversity, all with fully sequenced and annotated genomes. This resulted in 18 IRX10 homologs from *Physcomitrella patens* (1), *Selaginella moellendorffii* (2), *Arabidopsis thaliana* (3), *Populus trichocarpa* (4), *Brachypodium distachyon* (5) and *Oryza sativa* (6). Table 3 shows the pair-wise amino acid maximum identity scores using the BLAST algorithm (Altschul et al., 1997; http://blast.ncbi.nlm.nih.gov/Blast.cgi) for these 18 IRX10 proteins compared against Arabidopsis IRX10 (AtIRX10) and the six PoIRX10. Arabidopsis FRA8 and XGD1 were included for comparison of more distantly related genes. FRA8 is the closest homolog to the IRX10(-L) genes in Arabidopsis (Zhong et al., 2005) and XGD1 is a xylosyltransferase from GT47 subgroup D (Jensen et al., 2008). The remaining of the pair-wise matrix is shown Table S2 in Supplementary Material. Eudicot sequences, including PoIRX10_3, _5 and _6, share 81–91% identity with AtIRX10, while monocot sequences show 76–87% identity with AtIRX10. Remarkably, the evolutionarily more distant SmIRX10 and PpIRX10 follow a similar trend with 86% and 77% identity, respectively, to AtIRX10. This conservation is also observed when comparing SmIRX10 and PpIRX10 to the remaining sequences from poplar, *B. distachyon* and rice; here SmIRX10 shows 77–88% identity, while PpIRX10 shows 68 to 80% identity (Table S2 in Supplementary material). The difference in identity between PoIRX10_1, _2 and _4 and mono- and dicot IRX10s is similar or lower than the difference in identity between higher plants and PpIRX10. Thus, IRX10 proteins show a high degree of conservation over the phylogenetic distance from *P. patens* to higher plants, while three of the four ML PoIRX10 proteins show notably less conservation, with PoIRX10_4 being the most divergent.

**Table 3:**
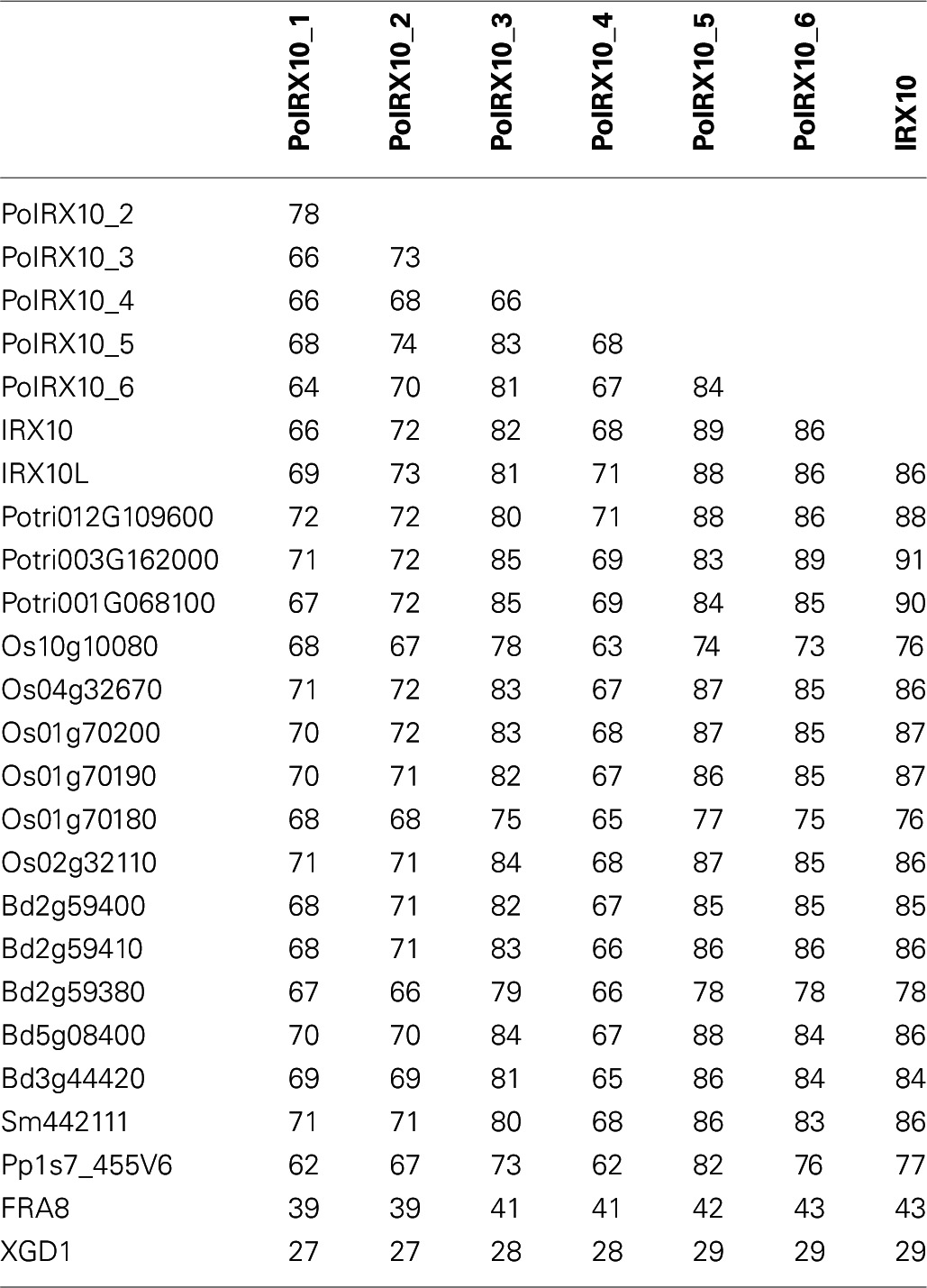
**Pairwise amino acid maximum identity scores using BLAST^a^**.

^a^ (Altschul et al., 1997); http://blast.ncbi.nlm.nih.gov/Blast.cgi

**Figure 3:**
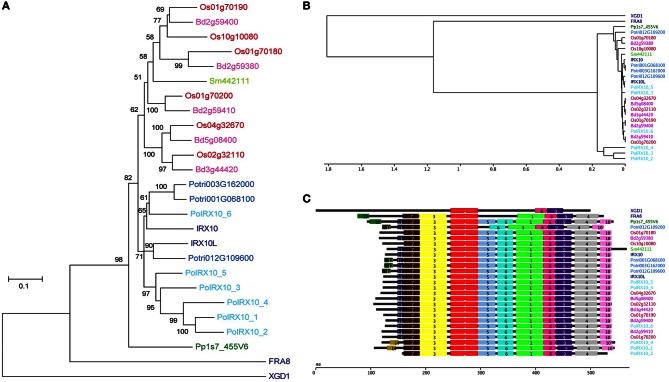
**Phylogenetic and motif analysis of IRX10 homologs in psyllium. (A)** Phylogenetic analysis of IRX10 homologs in psyllium (light blue) and six other plants; *Brachypodium distachyon* (pink), rice (red), Arabidopsis (dark blue), poplar (blue), *Selaginella moellendorffii* (light green) and *Physcomitrella patens* (green). **(B, C)** Hierarchical clustering **(B)** of motif analysis **(C)** generated using the interactive feature in the SALAD database (http://salad.dna.affrc.go.jp/CGViewer/en/cgv_upload.html). Both graphs are provided in File S3 including bootstrap values from the hierarchical clustering.

A phylogenetic tree of the 24 IRX10 proteins, FRA8 and XGD1 is shown in Figure 3A. The phylogenetic analysis was performed on a codon based cDNA sequence alignment. This approach is beneficial when performing phylogenetic analysis of conserved proteins with many synonymous mutations. The tree identifies two major clades rooted by PpIRX10. Eudicot IRX10 sequences make up one of the major clades, while the other clade contains monocot IRX10 sequences and SmIRX10. Of the six psyllium proteins, PoIRX10_6 is grouped with AtIRX10 and two of the three poplar IRX10 proteins, while PoIRX10_1 to _5 form a separate group. The phylogenetic analysis therefore suggests that the expansion of PoIRX10 proteins has taken place after the separation of monocots and dicots.

Evaluation of evolutionarily conserved protein domains are a powerful method for predicting protein function and are collected in a number of searchable databases, e.g., Pfam (Punta et al., 2012) and InterPro (Hunter et al., 2009). The algorithm behind the SALAD database uses patterns of evolutionarily conserved motifs to determine relatedness (Mihara et al., 2010; http://salad.dna.affrc.go.jp/salad/en/). As with other protein domain predicting methods, this approach emphasizes conserved protein function rather than phylogenetic relationships. In Figure 3B the 26 proteins from Figure 3A are depicted in a SALAD dendrogram. It shows that IRX10 proteins ranging in phylogenetic distance from *P. patens* to Arabidopsis are tightly clustered while PoIRX10_1, _2 and _4 form a distinct group. Notably, this psyllium specific clade consists of PoIRX10 proteins exclusively expressed in the ML. The SALAD motif structure (Figure 3C), used to construct the dendrogram, is conserved across the majority of IRX10 proteins. A few exceptions exist such as motif 5 is absent in the poplar gene Potri012G109200, motif 10 is absent in PoIRX10_2 and there is some motif variation in the N-terminus involving motif 11, 12, 14, and 15. In FRA8 motif 5, 6, and 10 are absent; while in XGD1 most of the motifs found in the IRX10 proteins are absent. This indicates that PoIRX10_1, _2 and _4 have conserved the motif structure despite their more divergent protein sequences and suggests they have conserved protein function with the IRX10 proteins found in the other plant species.

### Similarities in xylan side chain decorations between psyllium and grasses are likely the result of convergent evolution

The psyllium database contains 18 contigs encoding proteins with close homology to AT3G18170 and AT3G18180. Many of these contigs represented partial transcripts and were assembled into full transcripts by manual inspection. These efforts yielded evidence for the presence of nine unique GT61 genes in psyllium, seven of which were cloned from cDNA and named *Plantago ovata GT61 1* to *7* (*PoGT61_1* to *_7*).

**Figure 4:**
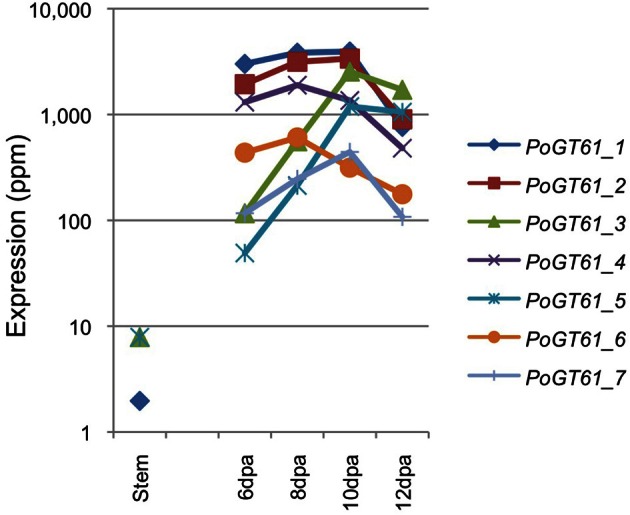
**Expression levels of glycosyltransferase family 61 genes in psyllium in stem and mucilaginous layers**.

The expression profiles of *PoGT61_1* to *_7* in psyllium stem and ML are depicted in Figure 4. These expression levels were similarly high as those of the *PoIRX10_1* to *_4* genes in the ML and show either induction or flat to decreasing levels of expression during ML development. These proteins are therefore likely candidates for GT activities that form the side chain decorations on the ML complex heteroxylan.

**Figure 5:**
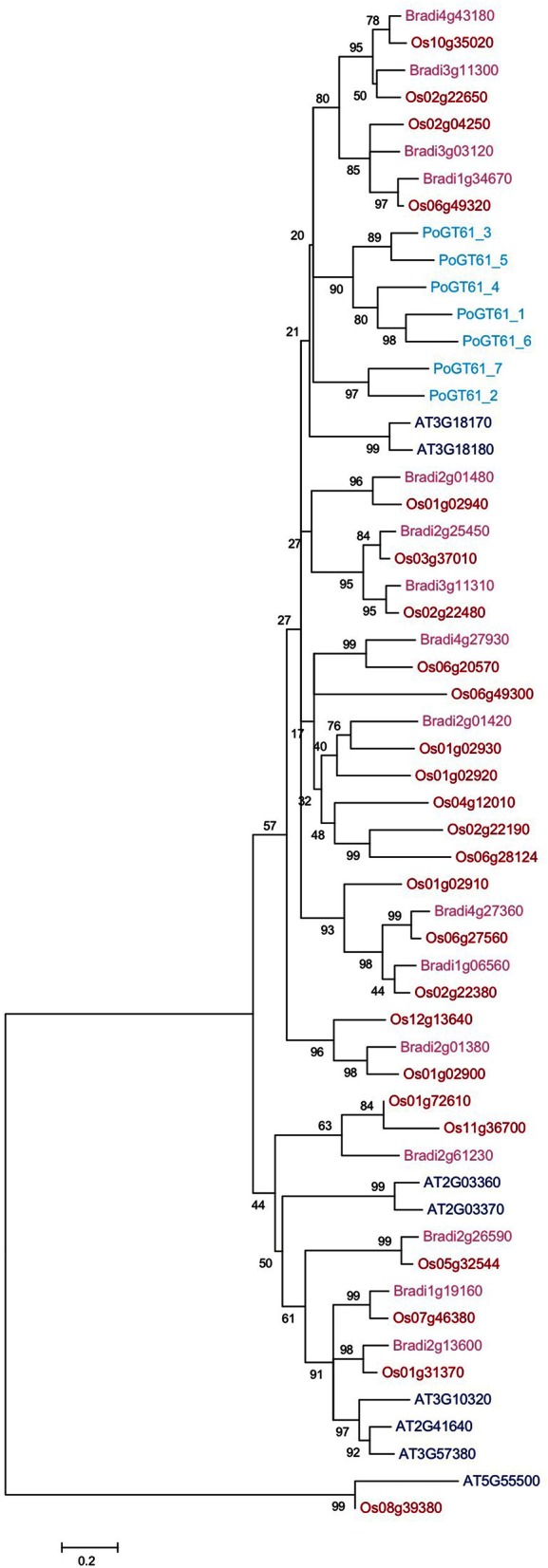
**Phylogenetic analysis of glycosyltransferase family 61 proteins from psyllium, Arabidopsis, rice and *Brachypodium distachyon***. Seven cDNAs displaying homology to At3g18170 and At3g18180 were cloned from psyllium mucilaginous layers and their full-length protein sequences deduced. A few transcripts encoding protein sequences homologous to some of the other six GT61 proteins in Arabidopsis were identified in the mucilagionous layers but these were expressed at negligible levels (<10 pm) and were not included in this analysis. The seven GT61 proteins highly expressed in psyllium mucilaginous layers (light blue) were aligned with all glycosyltransferase family 61 proteins from Arabidopsis (dark blue), rice (red) and *Brachypodium distachyon* (pink).

Figure 5 presents a phylogenetic tree of *PoGT61_1* to *_7* and all GT61 proteins identified in Arabidopsis, rice and *B. distachyon* (ClustalW alignment in File S4). The phylogenetic tree shows that the large diversification in grasses of this family is unrelated to the diversification found in psyllium. Therefore, the similar modifications of the xylan backbone found in psyllium ML and grasses are likely the results of convergent evolution.

### Possible function of the numerous putative glycosyltransferases highly expressed in psyllium mucilaginous layer

The structure of the xylan-based mucilage from the *Plantago* genus (*ovata* F., *major* L., *asiatica* L.) is highly complex (Samuelsen et al., 1999; Fischer et al., 2004; Yin et al., 2012). In the work of Guo et al. (2008) psyllium husk was fractionated using hot water and successive rounds of increasing concentrations of NaOH. This resulted in three fractions collectively accounting for 90% of the husk mass and predominantly consisting of Ara (15–25%) and Xyl (65–70%). Two of these fractions also yielded approximately 15% uronic acid. Each of the three fractions showed related but distinct glycosyl-linkage compositions providing evidence for the presence of extensively branched xylans in all three fractions. In all fractions, the branching appears to consist of single xylose residues, single arabinose residues and side chains of two to three sugars containing different combinations of xylose, arabinose, galactose, and mannose (Guo et al., 2008). An abundant side chain of α-Ara*f*-(1→3)-β-Xyl*p*-(1→3)-Ara*f* present in the non-acidic fraction has been isolated and structurally characterized by NMR (Fischer et al., 2004). It therefore appears that the mucilage of *P. ovata* F. consist of several species of complex heteroxylans that have different structural compositions and physical characteristics.

The multiple side chains found in the psyllium mucilage are consistent with finding numerous GTs highly expressed in the ML. The identification of four different and abundantly expressed PoIRX10 genes is noteworthy. This may indicate that there are several heteroxylan subspecies being produced in the tissue and that each PoIRX10 protein is involved in making separate xylans by interacting with different decorating GTs. Alternatively, the four PoIRX10 proteins could form one or more complexes necessary to form the β-(1,4)-xylan backbone. Thirdly, some of the PoIRX10 proteins may be backbone decorating GTs and not involved in backbone synthesis (Table S3 in Supplementary Material). It seems likely, however, that at least one of the ML specific PoIRX10 proteins constitutes the xylan synthase in this tissue, hence forming a xylan synthase activity different than that found in Arabidopsis and other eudicots.

Several GTs from rice and wheat have been implicated in xylan side-chain formation. TaXAT2, OsXAT2 and OsXAT3 are xylan α-(1,3)-arabinosyltransferases, transferring single arabinofuranose onto the xylan backbone at the O-3 position (Anders et al., 2012). While XAX1 from rice is likely a β-(1,2)-xylosyltransferase involved in making the side-chain β-Xyl*p*-(1→2)-α-Ara*f*-(1→3) (Chiniquy et al., 2012). These proteins all group together in the same phylogenetic subgroup as Arabidopsis AT3G18170 and AT3G18180 and PoGT61_1 to _7. Therefore, it seems likely that the seven PoGT61 proteins are arabinosyl- and xylosyltransferases involved in forming the large diversity of xylan side-chains found in psyllium heteroxylan (Table S3 in Supplementary Material).

Small amounts of rhamnose, glucose, glucuronic acid, galactose, and mannose have been identified in psyllium husk and proposed to be side chain decorations (Fischer et al., 2004; Guo et al., 2008; Yin et al., 2012). Additional GTs, from families other than GT61, may be involved in forming these side chains in psyllium heteroxylan. Two such candidates are the transcripts homologous to AT4G32290 and AT2G32750 (Table S3 in Supplementary Material). AT4G32290 is a member of the GT14R family (Nikolovski et al., 2012). None of the members in this family have been characterized apart from having Golgi localization. The homologous transcript in psyllium is highly abundant in the ML. AT2G32750 is homologous to Arabidopsis MUR3 (Madson et al., 2003) and RLXT2 (Jensen et al., 2012), both of which transfer galactose onto xylose as part of xyloglucan biosynthesis. The homologous protein in psyllium is copiously expressed in the ML and could possibly transfer galactose onto xylose in psyllium heteroxylan.

### Putative nucleotide sugar transporters are highly expressed in psyllium mucilaginous layers

Golgi transport proteins for UDP-galactose, UDP-galactose/glucose and GDP-mannose have been identified in Arabidopsis (Reyes and Orellana, 2008; Handford et al., 2012) and rice (Seino et al., 2010) and are members of the NST/TPT superfamily (Ward, 2001). Proteins transporting other UDP-sugars have been proposed to also be members of this superfamily (Ward, 2001; Reyes and Orellana, 2008). Several UDP-sugar transporters are likely to be expressed in the ML in order to supply UDP-xylose and UDP-arabino*furanose* to Golgi localized enzymes for the biosynthesis of complex heteroxylan. The UDP-arabino*pyranose* mutase (UAM) interconverts UDP-arabino*furanose* and UDP-arabino*pyranose* (Konishi et al., 2007) and is located in the cytosol (Bar-Peled and O'Neill, 2011). The synthesis of arabinoxylan occurs in the Golgi and requires UDP-arabino*furanose*, which appears to be uniquely produced by this mutase (Rautengarten et al., 2011). It is therefore necessary for UDP-arabino*furanose* to be transported across the Golgi membrane in order for it to be incorporated into cell wall carbohydrates such as heteroxylan. Approximately 40% of the neutral sugar content of the ML cell wall is arabinose, likely requiring higher amounts of UDP-arabino*furanose* import into ML Golgi. It is therefore likely that transcript levels for the UDP-arabino*furanose* transporter would be high in the ML.

The enzyme UDP-xylose epimerase 1 (UXE1/MUR4), that interconverts UDP-xylose and UDP-arabino*pyranose*, has been found to be Golgi localized in Arabidopsis (Burget et al., 2003). Two contigs in the psyllium ML (M01000013775 and M01000025234) match sequences at the N-terminal and C-terminal of UXE1 and together may represent the full-length transcript of a psyllium UXE1 homolog. This homolog shares 81% amino acid sequence identity with UXE1 in the N-terminal where both proteins have transmembrane domains as predicted by the TMHMM Server v. 2.0. The psyllium UXE1 homolog is therefore likely a Golgi localized protein. To provide UDP-xylose for UXE1, psyllium appears to express two isoforms of UDP-xylose synthase (UXS) at comparable levels in the ML (1000–6000 ppm) with just one of the two having a predicted transmembrane domain. Psyllium therefore appears to have the capacity to produce UDP-xylose in the cytosol, as well as in the Golgi. Finally, the substrate for UXS, UDP-glucuronic acid, is usually synthesized by the enzyme UDP-glucose 6-dehydrogenase (UGD) from UDP-glucose. The subcellular localization of this enzyme in psyllium ML could not be inferred as the contig lacks the N-terminal sequences, which would contain the transmembrane domain. The putative subcellular localization of these UDP-sugar interconverting activities in psyllium ML present several possible routes for the supply of UDP-xylose needed for xylan biosynthesis. The UDP-sugars imported into Golgi may be UDP-glucose, UDP-glucuronic acid or UDP-xylose. Furthermore, as the psyllium ML UXE activity appears to be exclusively Golgi localized, UDP-arabino*pyranose* needs to be exported from Golgi to the cytosol in order to be converted to UDP-arabino*furanose* by UMA. Hence, psyllium transporters of these UDP-sugars, as well as UDP-arabino*furanose* as mentioned above, are likely to be expressed at elevated levels in the ML.

The psyllium database holds a total of 50 contigs encoding proteins with close homology to the Arabidopsis NST/TPT Family. Homologs of characterized proteins such as ATUTR3 (Reyes et al., 2010) and GONST1 (Baldwin et al., 2001) are found at nearly undetectable levels in the ML, while the most abundant transcripts reach expression levels as high as 2000 ppm. When ranked by abundance in the ML 10 DPA stage, three of the top four transcripts show homology to Arabidopsis NST proteins AT5G25400, AT1G21070 and AT4G32390, all of which are in an uncharacterized branch of the NST/TPT superfamily. Several members of this branch, including AT4G32390, have been found to be localized in the Golgi (Nikolovski et al., 2012). The second most abundant transcript has closest homology to AT1G06890. This protein has also been found in Golgi (Nikolovski et al., 2012) and is related to GONST4 and GONST5 (Handford et al., 2004). These highly abundant NST transcripts might therefore be candidates for encoding UDP-glucose, UDP-glucuronic acid, UDP-xylose, UDP-arabino*pyranose*, or UDP-arabino*furanose* transport proteins.

### Additional genes possibly involved in xylan biosynthesis in psyllium mucilaginous layers

Identifying an Arabidopsis gene with secondary cell wall expression and with a close homolog highly expressed in psyllium ML may indicate that such a gene is involved in xylan biosynthesis in Arabidopsis and psyllium ML, as has proved to be the case for the Arabidopsis IRX15(-L) proteins (Jensen et al., 2011). Table 4 shows the 12 most abundant transcripts in psyllium that show a similar expression pattern. These genes are likely involved in complex heteroxylan biosynthesis or in secondary cell wall formation associated with the psyllium ML.

**Table 4:** **The 12 most abundant transcripts expressed in psyllium mucilaginous layers where the closest homolog in Arabidopsis is co-expressed with IRX10 or CESA4, CESA7 and CESA8**.

**Contig**	**AGI**	**Gene name**	**6 DPA^a^^,^^b^**	**8 DPA**	**10 DPA^c^**	**12 DPA**	**Stem**
M01000012773	AT1G29050	Trichome birefringence-like 38	2252	2164	2784	1990	43
M01000017716	AT2G28760	UXS6	6524	3787	1200	613	203
M01000007254	AT1G75680	Glycosyl hydrolase 9B7	559	623	1092	549	244
M01000007937	AT5G67210	IRX15-L	1178	926	994	887	10
M01000025167	AT5G47635	Pollen Ole e 1 allergen and extensin family protein	2105	1320	817	353	6
M01000007306	AT2G03200	Eukaryotic aspartyl protease family protein	632	680	698	1093	14
M01000032225	AT2G28250	Protein kinase superfamily protein	80	107	689	480	47
M01000021799	AT1G19835	DUF869	153	148	679	98	98
M01000007966	AT2G12400	Unknown protein	466	607	669	720	35
M01000007257	AT1G27440	IRX10	423	279	639	255	14
M01000012823	AT3G15050	IQ-domain 10	147	189	541	294	83
M01000025210	AT4G18780	CESA8	92	82	384	196	43

a Days post anthesis, DPA.

b Expression data is in parts per million (ppm).

c Transcripts are ranked by expression in the mucilaginous layers at the 10 DPA stage.

**Figure 6:**
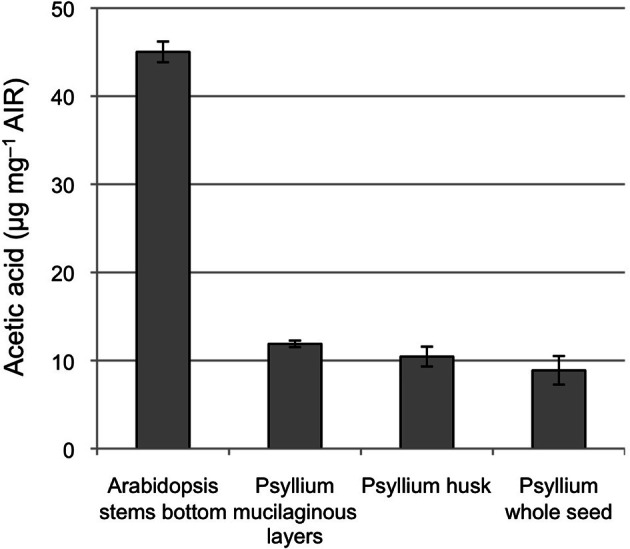
**Acetylation levels in alcohol insoluble residue of psyllium seed tissues**.

The top member is homologous to Arabidopsis TBR38 and contains a domain of unknown function (DUF) 231. The DUF231 proteins constitute a 46-member protein family in Arabidopsis (Bischoff et al., 2010) in which the genes AXY4 (TBL27) and AXY4-Like (TBL22) have been shown to be involved in acetylation of xyloglucan (Gille et al., 2011) and ESK1 (TBL29) have been shown to be involved in acetylation of secondary cell wall xylan (Xiong et al., 2013). The other members of this family have been proposed to also be acetyltransferases specific for xyloglucan or other cell wall polymers, e.g., pectins and xylan (Oikawa et al., 2010; Gille and Pauly, 2012). TBR38 is part of an uncharacterized subclade of the TBR protein family. Given that the psyllium homolog has a much higher expression in the ML than the secondary cell wall CESA proteins it is likely involved in complex heteroxylan biosynthesis rather than secondary cell wall formation in this tissue. The level of cell wall acetylation in dissected psyllium ML is 12 μg acetic acid per milligram alcohol insoluble residue, approximately 4 fold lower that found in the alcohol insoluble residue of Arabidopsis lower stem (Figure 6). The acetic acid content in the psyllium ML corresponds to one acetic acid group for every 25 pentose sugars, assuming the cell wall material from the ML consist of 100% pentose sugars. Glucuronoxylan from aspen wood has been found to have an average degree of xylose backbone acetylation of approximately 60% (Teleman et al., 2000), while a degree of acetylation of approximately 50% has been found for arabinoxylan from corncobs and corn stover (Dongen et al., 2011). These findings may indicate that TBR38 and its psyllium homolog could function as xylan specific acetyltransferases.

Another candidate gene possibly involved in xylan formation in psyllium ML is a homolog of At5g47635 encoding a Pollen Ole e 1 allergen and extensin family protein. The work of Tan et al. (2013) identified and characterized two isoforms of a highly glycosylated AGP, named ARABINOXYLAN PECTIN ARABINOGALACTAN PROTEIN1 (APAP1). The authors identified two individual xylan oligomers attached as separate side chains of the APAP1 carbohydrate branch structure and so provide a link between AGP and xylan. Though other possibilities exist, the high expression of an extensin protein in psyllium ML and the secondary cell wall expression pattern of the closest Arabidopsis homolog may suggest that this extensin homolog functions by cross-linking mucilaginous heteroxylan into a bigger covalent network in the mucilage wall.

### Homologs of several secondary cell wall transcription factors are highly expressed in psyllium mucilaginous layers

The most abundant psyllium transcripts encoding putative transcription factors reach levels of 1000 ppm in the ML (Table 5). Many of these transcripts show closest homology to Arabidopsis genes that are highly expressed throughout the Arabidopsis plant, including seed development, while another set of these transcripts have close homology to Arabidopsis genes that are specifically expressed during seed development, such as MYB61, NARS1, AT3G51880 and AT5G67480. Both MYB61 and NARS1 have been shown to play roles in seed coat development in Arabidopsis. Knockout mutants of *MYB61* have reduced mucilage deposition and extrusion (Penfield et al., 2001), while NARS1 is expressed in the outer integument of the Arabidopsis seed where it regulates the degeneration of this tissue (Kunieda et al., 2008).

**Table 5:** **The 20 most abundant transcripts expressed in psyllium mucilaginous layers encoding putative transcription factors**.

**Contig**	**AGI**	**Gene name**	**6 DPA^a^^,^^b^**	**8 DPA**	**10 DPA^c^**	**12 DPA**	**Stem**
M01000026602	AT1G06760	Winged-helix DNA-binding transcription factor family	479	877	1358	985	303
M01000012738	AT3G15510	NARS1	246	312	1102	774	6
M01000012705	AT1G24260	SEP3, AGL9, K-box region and MADS-box transcription factor	810	853	1003	1603	0
M01000032308	AT3G14230	Related to AP2 2	454	574	728	1431	299
M01000017075	AT3G58680	Multiprotein bridging factor 1B	436	295	571	436	71
M01000007740	AT2G28350	Auxin response factor 10	203	189	531	470	26
M01000012861	AT1G78080	Related to AP2 4	86	74	374	402	159
M01000032317	AT3G51880	High mobility group B1	423	402	374	363	110
M01000026318	AT3G62420	Basic region/leucine zipper motif 53	172	180	354	211	138
M01000011973	AT2G46770	NST1, ANAC043	344	467	334	64	6
M01000025410	AT4G28500	SND2, ANAC073	485	303	334	25	12
M01000009134	AT5G65670	IAA9	288	246	325	230	385
M01000027924	AT1G20696	High mobility group B3	239	434	305	333	65
M01000008289	AT2G01570	GRAS family transcription factor	184	90	285	353	242
M01000007859	AT1G62990	KNAT7	153	246	266	255	49
M01000013016	AT5G67480	BTB and TAZ domain protein 4	68	98	266	49	14
M01000007647	AT1G09540	MYB61	196	172	246	59	4
M01000032322	AT3G02790	Zinc finger (C2H2 type) family protein	282	320	236	490	57
M01000032047	AT1G05380	Acyl-CoA N-acyltransferase with RING/FYVE/PHD-type zinc finger protein	25	25	226	20	67
M01000008230	AT1G11950	Transcription factor jmjC domain-containing protein	135	98	216	10	185

a Days post anthesis, DPA.

b Expression data is in parts per million (ppm).

c Transcripts are ranked by expression in the mucilaginous layers at the 10 DPA stage.

A third category of transcripts consists of ones with closest homology to Arabidopsis transcription factors involved in secondary cell wall formation, namely NST1, SND2, and KNAT7. All three transcription factors are potent regulators of secondary cell wall formation in Arabidopsis. NST1 was identified as a regulator of secondary wall thickening in anther endothecium (Mitsuda et al., 2005) and was later found to act redundantly with SND1 as a master regulator of secondary wall synthesis in fiber cells of Arabidopsis stem (Zhong et al., 2007). Furthermore, in protoplast transactivation assays, NST1 directly activates *MYB46, SND3, MYB103*, and *KNAT7* (Zhong et al., 2008, 2010). Overexpression of SND2 also leads to increased secondary wall thickening in Arabidopsis stem fiber cells (Zhong et al., 2008) and upregulation of, among other genes, *MYB103* and *SND1* (Hussey et al., 2011). *KNAT7* loss-of-function mutants display *IRX* phenotypes (Brown et al., 2005) and Arabidopsis plants transformed with dominant repression constructs of *KNAT7* lead to a moderate decrease of secondary cell wall thickening in Arabidopsis stems (Zhong et al., 2008). The KNAT7 protein has been shown to interact with OFP4 and both act as repressor proteins in protoplast transactivation assays and *in planta* (Li et al., 2011, 2012). The homologs of NST1, SND2, and KNAT7 found in psyllium ML may or may not be true orthologs to the Arabidopsis genes. However, it is striking to find several abundantly expressed homologs of transcription factors that have been implicated in secondary cell wall formation, a process involving extensive biosynthesis of xylan. The highly elevated expression levels of homologs NST1, SND2 and KNAT7 in the psyllium ML may therefore suggest that they are involved in regulating xylan biosynthesis in this tissue. Such regulatory circuit(s) in the psyllium ML may have evolved from the secondary cell wall regulatory cascade. It should be noted that this it is only a partial set of the transcriptional regulatory network controlling secondary cell wall formation in Arabidopsis (Demura and Ye, 2010) that may be detected in the psyllium ML. Of the proven downstream targets for NST1 and SND2, it is only a homolog of KNAT7 that is found highly expressed in the psyllium ML. Homologs of proven targets of NST1 in Arabidopsis, such as SND1, MYB46, and MYB103, are not detected, while a homolog of SND3 is detected but at low levels of approximately 50 ppm. When over-expressed, NST1 will induce abundant secondary cell wall formation in Arabidopsis mesophyll leaf cells (Zhong et al., 2007). If the psyllium homolog of NST1 found in the ML is functionally othologous to Arabidopsis NST1, it appears that branches of the NST1 transcriptional cascade that leads to cellulose and lignin deposition, rather than xylan formation, has been specifically suppressed in the psyllium ML.

### Lessons learned from xylan biosynthesis in psyllium mucilaginous layers may prove valuable for biofuels research and biotechnology

The study of tissues having cell walls with unusual composition may provide valuable insights into manipulating plant cell walls for improved characteristics as biofuel feedstocks, such as improved digestibility, higher biomass, and altered composition of lignin, cellulose, and hemicellulose. It seems plausible that the diverse cell walls found in many highly specialized tissues, for instance in many seeds, are derived from existing cell wall biosynthetic pathways and so provide examples of cell wall alterations which provide new characteristics.

This study provides evidence for biosynthetic enzymes, sugar-nucleotide transporters and transcription factors as likely candidates involved in xylan biosynthesis. These new targets may serve as novel entry points to manipulate xylan deposition and structure. To date, it has not been possible to reconstitute the xylan synthase activity from known components. This has limited our ability to assign roles for the genes shown to be components of the synthase by genetic methods. The four cloned PoIRX10 from the ML may constitute a simpler xylan synthase, as it has a reduced set of components, suggesting that it may be more tractable than xylan synthases from systems such as Arabidopsis. If so, the psyllium *IRX10* genes would offer a tool for future research in understanding and manipulating xylan formation.

The seven cloned PoGT61 sequences may prove useful in altering xylan branch structures in cell walls of both monocot and eudicot crops for improved biofuel traits such as digestibility. Finally, identification of direct transcriptional regulators of xylan biosynthetic genes, such as *IRX10*, is likely to identify more genes involved in xylan biosynthesis which could constitute key points of regulating xylan biosynthesis.

Full access has been provided to the RNA-Seq data from psyllium through a user-friendly web interface. The database features several custom made tools facilitating further analysis and may provide a valuable resource for the research community in other areas than xylan biosynthesis, such as mucilage development.

### Conflict of interest statement

The authors declare that the research was conducted in the absence of any commercial or financial relationships that could be construed as a potential conflict of interest.

## Supplementary material

File S1 **Transmembrane domain predictions for PoIRX10_1, PoIRX10_3, and PoIRX10_4**.

File S2 **Protein sequence alignment of IRX10 proteins**.

File S3 **SALAD dendrogram and motif alignment of IRX10 proteins**.

File S4 **Protein sequence alignment of GT61 proteins**.

Table S1 **454 FLX sequencing and assembly of sequence reads**.

Table S2 **Pairwise amino acid maximum identity scores using BLAST**.

Table S3 **Glycosyl linkages identified in psyllium mucilaginous heteroxylan and putative glycosyltransferases identified in this study that are hypothesized to form these linkages**.

## Abbreviations

GT: glycosyltransferase
DPA: days post anthesis
ML: mucilaginous layer.

## References

[B1] Altschul S. F. Madden T. L. Schäffer A. A. Zhang J. Zhang Z. Miller W. (1997). Gapped BLAST and PSI-BLAST: a new generation of protein database search programs. Nucleic Acids Res. 25, 3389–3402 10.1093/nar/25.17.3389 9254694 PMC146917

[B2] Anders N. Wilkinson M. D. Lovegrove A. Freeman J. Tryfona T. Pellny T. K. (2012). Glycosyl transferases in family 61 mediate arabinofuranosyl transfer onto xylan in grasses. Proc. Natl. Acad. Sci. U.S.A. 109, 989–993 10.1073/pnas.1115858109 22215597 PMC3271882

[B3] Arioli T. Peng L. Betzner A. S. Burn J. Wittke W. Herth W. (1998). Molecular analysis of cellulose biosynthesis in Arabidopsis. Science 279, 717–720 10.1126/science.279.5351.717 9445479

[B4] Baldwin T. C. Handford M. G. Yuseff M. I. Orellana A. Dupree P. (2001). Identification and characterization of GONST1, a golgi-localized GDP-mannose transporter in Arabidopsis. Plant Cell 13, 2283–2295 10.1105/tpc.010247 11595802 PMC139159

[B5] Bar-Peled M. O'Neill M. A. (2011). Plant nucleotide sugar formation, interconversion, and salvage by sugar recycling. Annu. Rev. Plant Biol. 62, 127–155 10.1146/annurev-arplant-042110-103918 21370975

[B6] Bischoff V. Nita S. Neumetzler L. Schindelasch D. Urbain A. Eshed R. (2010). TRICHOME BIREFRINGENCE and Its homolog AT5G01360 encode plant-specific DUF231 proteins required for cellulose biosynthesis in Arabidopsis. Plant Physiol. 153, 590–602 10.1104/pp.110.153320 20388664 PMC2879772

[B7] Brown D. Wightman R. Zhang Z. Gomez L. D. Atanassov I. Bukowski J.-P. (2011). Arabidopsis genes IRREGULAR XYLEM (IRX15) and IRX15L encode DUF579-containing proteins that are essential for normal xylan deposition in the secondary cell wall. Plant J. 66, 401–413 10.1111/j.1365-313X.2011.04501.x 21251108

[B8] Brown D. M. Zeef L. A. H. Ellis J. Goodacre R. Turner S. R. (2005). Identification of novel genes in Arabidopsis involved in secondary cell wall formation using expression profiling and reverse genetics. Plant Cell 17, 2281–2295 10.1105/tpc.105.031542 15980264 PMC1182489

[B9] Brown D. M. Zhang Z. Stephens E. Dupree P. Turner S. R. (2009). Characterization of IRX10 and IRX10-like reveals an essential role in glucuronoxylan biosynthesis in Arabidopsis. Plant J. 57, 732–746 10.1111/j.1365-313X.2008.03729.x 18980662

[B10] Burget E. G. Verma R. Mølhøj M. Reiter W.-D. (2003). The biosynthesis of L-arabinose in plants: molecular cloning and characterization of a Golgi-localized UDP-D-xylose 4-epimerase encoded by the MUR4 gene of Arabidopsis. Plant Cell 15, 523–531 10.1105/tpc.008425 12566589 PMC141218

[B11] Cantarel B. L. Coutinho P. M. Rancurel C. Bernard T. Lombard V. Henrissat B. (2009). The Carbohydrate-Active EnZymes database (CAZy): an expert resource for glycogenomics. Nucleic Acids Res. 37, D233–D238 10.1093/nar/gkn663 18838391 PMC2686590

[B12] Chen X.-Y. Liu L. Lee E. Han X. Rim Y. Chu H. (2009). The Arabidopsis callose synthase gene GSL8 is required for cytokinesis and cell patterning. Plant Physiol. 150, 105–113 10.1104/pp.108.133918 19286936 PMC2675722

[B13] Chiniquy D. Sharma V. Schultink A. Baidoo E. E. Rautengarten C. Cheng K. (2012). XAX1 from glycosyltransferase family 61 mediates xylosyltransfer to rice xylan. Proc. Natl. Acad. Sci. U.S.A. 109, 17117–17122 10.1073/pnas.1202079109 23027943 PMC3479505

[B14] Cocuron J.-C. Lerouxel O. Drakakaki G. Alonso A. P. Liepman A. H. Keegstra K. (2007). A gene from the cellulose synthase-like C family encodes a beta-1, 4 glucan synthase. Proc. Natl. Acad. Sci. U.S.A. 104, 8550–8555 10.1073/pnas.0703133104 17488821 PMC1895987

[B15] Demura T. Ye Z.-H. (2010). Regulation of plant biomass production. Curr. Opin. Plant Biol. 13, 299–304 10.1016/j.pbi.2010.03.002 20381410

[B16] Desprez T. Juraniec M. Crowell E. F. Jouy H. Pochylova Z. Parcy F. (2007). Organization of cellulose synthase complexes involved in primary cell wall synthesis in *Arabidopsis thaliana*. Proc. Natl. Acad. Sci. U.S.A. 104, 15572–15577 10.1073/pnas.0706569104 17878303 PMC2000492

[B17] Dhugga K. Barreiro R. Whitten B. Stecca K. Hazebroek J. Randhawa G. (2004). Guar seed beta-mannan synthase is a member of the cellulose synthase super gene family. Science 303, 363–366 10.1126/science.1090908 14726589

[B18] Dongen F. E. M. V. Eylen D. V. Kabel M. A. (2011). Characterization of substituents in xylans from corn cobs and stover. Carbohydr. Polym. 86, 722–731 10.1016/j.carbpol.2011.05.007

[B19] Edwards M. E. Dickson C. A. Chengappa S. Sidebottom C. Gidley M. J. Reid J. S. (1999). Molecular characterisation of a membrane-bound galactosyltransferase of plant cell wall matrix polysaccharide biosynthesis. Plant J. 19, 691–697 10.1046/j.1365-313x.1999.00566.x 10571854

[B20] Edwards S. Chaplin M. F. Blackwood A. D. Dettmar P. W. (2003). Primary structure of arabinoxylans of ispaghula husk and wheat bran. Proc. Nutr. Soc. 62, 217–222 10.1079/PNS2003202 12756970

[B21] Felsenstein J. (1985). Confidence limits on phylogenies: an approach using the bootstrap. Evolution 39, 783–791 10.2307/2408678 28561359

[B22] Fischer M. H. Yu N. Gray G. R. Ralph J. Anderson L. Marlett J. A. (2004). The gel-forming polysaccharide of psyllium husk (Plantago ovata Forsk). Carbohydr. Res. 339, 2009–2017 10.1016/j.carres.2004.05.023 15261594

[B23] Gille S. De Souza A. Xiong G. Benz M. Cheng K. Schultink A. (2011). O-acetylation of Arabidopsis hemicellulose xyloglucan requires AXY4 or AXY4L, proteins with a TBL and DUF231 domain. Plant Cell 23, 4041–4053 10.1105/tpc.111.091728 22086088 PMC3246330

[B24] Gille S. Pauly M. (2012). O-acetylation of plant cell wall polysaccharides. Front. Plant Sci. 3:12 10.3389/fpls.2012.00012 22639638 PMC3355586

[B25] Goodstein D. M. Shu S. Howson R. Neupane R. Hayes R. D. Fazo J. (2012). Phytozome: a comparative platform for green plant genomics. Nucleic Acids Res. 40, D1178–D1186 10.1093/nar/gkr944 22110026 PMC3245001

[B26] Goto N. (1985). A mucilage polysaccharide secreted from testa of *Arabidopsis thaliana*. Arab. Inf. Ser. 22, 143

[B27] Goubet F. Barton C. J. Mortimer J. C. Yu X. Zhang Z. Miles G. P. (2009). Cell wall glucomannan in Arabidopsis is synthesised by CSLA glycosyltransferases, and influences the progression of embryogenesis. Plant J. 60, 527–538 10.1111/j.1365-313X.2009.03977.x 19619156

[B28] Guo A. He K. Liu D. Bai S. Gu X. Wei L. (2005). DATF: a database of Arabidopsis transcription factors. Bioinformatics 21, 2568–2569 10.1093/bioinformatics/bti334 15731212

[B29] Guo Q. Cui S. W. Wang Q. Young J. C. (2008). Fractionation and physicochemical characterization of psyllium gum. Carbohydr. Polym. 73, 35–43 10.1016/j.carbpol.2007.11.001

[B30] Handford M. Rodríguez-Furlán C. Marchant L. Segura M. Gómez D. Alvarez-Buylla E. (2012). *Arabidopsis thaliana* AtUTr7 encodes a golgi-localized UDP-glucose/UDP-galactose transporter that affects lateral root emergence. Mol. Plant 5, 1263–1280 10.1093/mp/sss074 22933714

[B31] Handford M. G. Sicilia F. Brandizzi F. Chung J. H. Dupree P. (2004). *Arabidopsis thaliana* expresses multiple Golgi-localised nucleotide-sugar transporters related to GONST1. Mol. Genet. Genom. 272, 397–410 10.1007/s00438-004-1071-z 15480787

[B32] Hunter S. Apweiler R. Attwood T. K. Bairoch A. Bateman A. Binns D. (2009). InterPro: the integrative protein signature database. Nucleic Acids Res. 37, D211–D215 10.1093/nar/gkn785 18940856 PMC2686546

[B33] Hussey S. G. Mizrachi E. Spokevicius A. V. Bossinger G. Berger D. K. Myburg A. A. (2011). SND2, a NAC transcription factor gene, regulates genes involved in secondary cell wall development in Arabidopsis fibres and increases fibre cell area in Eucalyptus. BMC Plant Biol. 11:173 10.1186/1471-2229-11-173 22133261 PMC3289092

[B34] International Brachypodium Initiative. (2010). Genome sequencing and analysis of the model grass *Brachypodium distachyon*. Nature 463, 763–768 10.1038/nature08747 20148030

[B35] Jensen J. K. Schultink A. Keegstra K. Wilkerson C. G. Pauly M. (2012). RNA-Seq analysis of developing nasturtium seeds (*Tropaeolum majus*): identification and characterization of an additional galactosyltransferase involved in xyloglucan biosynthesis. Mol. Plant 5, 984–992 10.1093/mp/sss032 22474179 PMC3440008

[B36] Jensen J. K. Kim H. Cocuron J.-C. Orler R. Ralph J. Wilkerson C. G. (2011). The DUF579 domain containing proteins IRX15 and IRX15-L affect xylan synthesis in Arabidopsis. Plant J. 66, 387–400 10.1111/j.1365-313X.2010.04475.x 21288268

[B37] Jensen J. K. Sørensen S. O. Harholt J. Geshi N. Sakuragi Y. Møller I. (2008). Identification of a xylogalacturonan xylosyltransferase involved in pectin biosynthesis in Arabidopsis. Plant Cell 20, 1289–1302 10.1105/tpc.107.050906 18460606 PMC2438468

[B38] Konishi T. Takeda T. Miyazaki Y. Ohnishi-Kameyama M. Hayashi T. O'Neill M. A. (2007). A plant mutase that interconverts UDP-arabinofuranose and UDP-arabinopyranose. Glycobiology 17, 345–354 10.1093/glycob/cwl081 17182701

[B39] Krogh A. Larsson B. Von Heijne G. Sonnhammer E. L. (2001). Predicting transmembrane protein topology with a hidden Markov model: application to complete genomes. J. Mol. Biol. 305, 567–580 10.1006/jmbi.2000.4315 11152613

[B40] Kunieda T. Mitsuda N. Ohme-Takagi M. Takeda S. Aida M. Tasaka M. (2008). NAC family proteins NARS1/NAC2 and NARS2/NAM in the outer integument regulate embryogenesis in Arabidopsis. Plant Cell 20, 2631–2642 10.1105/tpc.108.060160 18849494 PMC2590734

[B41] Larkin M. A. Blackshields G. Brown N. P. Chenna R. Mcgettigan P. A. Mcwilliam H. (2007). Clustal W and clustal X version 2.0. Bioinformatics 23, 2947–2948 10.1093/bioinformatics/btm404 17846036

[B42] Lee C. Teng Q. Huang W. Zhong R. Ye Z.-H. (2010). The Arabidopsis family GT43 glycosyltransferases form two functionally nonredundant groups essential for the elongation of glucuronoxylan backbone. Plant Physiol. 153, 526–541 10.1104/pp.110.155309 20335400 PMC2879797

[B43] Lee C. Teng Q. Zhong R. Ye Z.-H. (2012a). Arabidopsis GUX proteins are glucuronyltransferases responsible for the addition of glucuronic acid side chains onto xylan. Plant Cell Physiol. 53, 1204–1216 10.1093/pcp/pcs064 22537759

[B44] Lee C. Teng Q. Zhong R. Yuan Y. Haghighat M. Ye Z.-H. (2012b). Three Arabidopsis DUF579 domain-containing GXM proteins are methyltransferases catalyzing 4-O-methylation of glucuronic acid on xylan. Plant Cell Physiol. 53, 1934–1949 10.1093/pcp/pcs138 23045523

[B45] Li E. Bhargava A. Qiang W. Friedmann M. C. Forneris N. Savidge R. A. (2012). The class II KNOX gene KNAT7 negatively regulates secondary wall formation in Arabidopsis and is functionally conserved in Populus. New Phytol. 194, 102–115 10.1111/j.1469-8137.2011.04016.x 22236040

[B46] Li E. Wang S. Liu Y. Chen J.-G. Douglas C. J. (2011). OVATE FAMILY PROTEIN4 (OFP4) interaction with KNAT7 regulates secondary cell wall formation in *Arabidopsis thaliana*. Plant J. 67, 328–341 10.1111/j.1365-313X.2011.04595.x 21457372

[B47] Madson M. Dunand C. Li X. Verma R. Vanzin G. F. Caplan J. (2003). The MUR3 gene of Arabidopsis encodes a xyloglucan galactosyltransferase that is evolutionarily related to animal exostosins. Plant Cell. 15, 1662–1670 10.1105/tpc.009837 12837954 PMC165408

[B48] Mendu V. Stork J. Harris D. Debolt S. (2011). Cellulose synthesis in two secondary cell wall processes in a single cell type. Plant Signal. Behav. 6, 1638–1643 10.4161/psb.6.11.17709 22057330 PMC3329324

[B49] Mihara M. Itoh T. Izawa T. (2010). SALAD database: a motif-based database of protein annotations for plant comparative genomics. Nucleic Acids Res. 38, D835–D842 10.1093/nar/gkp831 19854933 PMC2808985

[B50] Mitsuda N. Seki M. Shinozaki K. Ohme-Takagi M. (2005). The NAC transcription factors NST1 and NST2 of Arabidopsis regulate secondary wall thickenings and are required for anther dehiscence. Plant Cell 17, 2993–3006 10.1105/tpc.105.036004 16214898 PMC1276025

[B51] Mortimer J. C. Miles G. P. Brown D. M. Zhang Z. Segura M. P. Weimar T. (2010). Absence of branches from xylan in Arabidopsis gux mutants reveals potential for simplification of lignocellulosic biomass. Proc. Natl. Acad. Sci. U.S.A. 107, 17409–17414 10.1073/pnas.1005456107 20852069 PMC2951434

[B52] Mutwil M. Obro J. Willats W. G. T. Persson S. (2008). GeneCAT–novel webtools that combine BLAST and co-expression analyses. Nucleic Acids Res. 36, W320–W326 10.1093/nar/gkn292 18480120 PMC2447783

[B53] Naran R. Chen G. Carpita N. C. (2008). Novel rhamnogalacturonan I and arabinoxylan polysaccharides of flax seed mucilage. Plant Physiol. 148, 132–141 10.1104/pp.108.123513 18667723 PMC2528086

[B54] Nei M. Kumar S. (2000). Molecular Evolution and Phylogenetics. New York, NY: Oxford University Press 10.1186/1471-2148-12-219

[B55] Nikolovski N. Rubtsov D. Segura M. P. Miles G. P. Stevens T. J. Dunkley T. P. (2012). Putative glycosyltransferases and other plant Golgi apparatus proteins are revealed by LOPIT proteomics. Plant Physiol. 160, 1037–1051 10.1104/pp.112.204263 22923678 PMC3461528

[B56] Oikawa A. Joshi H. J. Rennie E. A. Ebert B. Manisseri C. Heazlewood J. L. (2010). An integrative approach to the identification of Arabidopsis and rice genes involved in xylan and secondary wall development. PLoS ONE 5:e15481 10.1371/journal.pone.0015481 21124849 PMC2990762

[B57] Peña M. J. Zhong R. Zhou G.-K. Richardson E. A. O'Neill M. A. Darvill A. G. (2007). Arabidopsis irregular xylem8 and irregular xylem9: implications for the complexity of glucuronoxylan biosynthesis. Plant Cell 19, 549–563 10.1105/tpc.106.049320 17322407 PMC1867335

[B58] Pellny T. K. Lovegrove A. Freeman J. Tosi P. Love C. G. Knox J. P. (2012). Cell walls of developing wheat starchy endosperm: comparison of composition and RNA-Seq transcriptome. Plant Physiol. 158, 612–627 10.1104/pp.111.189191 22123899 PMC3271754

[B59] Penfield S. Meissner R. C. Shoue D. A. Carpita N. C. Bevan M. W. (2001). MYB61 is required for mucilage deposition and extrusion in the Arabidopsis seed coat. Plant Cell 13, 2777–2791 10.1105/tpc.010265 11752387 PMC139488

[B60] Persson S. Paredez A. Carroll A. Palsdottir H. Doblin M. Poindexter P. (2007). Genetic evidence for three unique components in primary cell-wall cellulose synthase complexes in Arabidopsis. Proc. Natl. Acad. Sci. U.S.A. 104, 15566–15571 10.1073/pnas.0706592104 17878302 PMC2000526

[B61] Persson S. Wei H. Milne J. Page G. P. Somerville C. R. (2005). Identification of genes required for cellulose synthesis by regression analysis of public microarray data sets. Proc. Natl. Acad. Sci. U.S.A. 102, 8633–8638 10.1073/pnas.0503392102 15932943 PMC1142401

[B62] Punta M. Coggill P. C. Eberhardt R. Y. Mistry J. Tate J. Boursnell C. (2012). The Pfam protein families database. Nucleic Acids Res. 40, D290–D301 10.1093/nar/gkr1065 22127870 PMC3245129

[B63] Rautengarten C. Ebert B. Herter T. Petzold C. J. Ishii T. Mukhopadhyay A. (2011). The interconversion of UDP-Arabinopyranose and UDP-Arabinofuranose is indispensable for plant development in Arabidopsis. Plant Cell 23, 1373–1390 10.1105/tpc.111.083931 21478444 PMC3101560

[B64] Rennie E. A. Hansen S. F. Baidoo E. E. K. Hadi M. Z. Keasling J. D. Scheller H. V. (2012). Three members of the Arabidopsis glycosyltransferase family 8 are xylan glucuronosyltransferases. Plant Physiol. 159, 1408–1417 10.1104/pp.112.200964 22706449 PMC3428776

[B65] Reyes F. León G. Donoso M. Brandizzí F. Weber A. P. M. Orellana A. (2010). The nucleotide sugar transporters AtUTr1 and AtUTr3 are required for the incorporation of UDP-glucose into the endoplasmic reticulum, are essential for pollen development and are needed for embryo sac progress in *Arabidopsis thaliana*. Plant J. 61, 423–435 10.1111/j.1365-313X.2009.04066.x 19906043

[B66] Reyes F. Orellana A. (2008). Golgi transporters: opening the gate to cell wall polysaccharide biosynthesis. Curr. Opin. Plant Biol. 11, 244–251 10.1016/j.pbi.2008.03.008 18485801

[B67] Samuelsen A. B. Cohen E. H. Paulsen B. S. Brüll L. P. Thomas-Oates J. E. (1999). Structural studies of a heteroxylan from Plantago major L. seeds by partial hydrolysis, HPAEC-PAD, methylation and GC-MS, ESMS and ESMS/MS. Carbohydr. Res. 315, 312–318 10.1016/S0008-6215(99)00038-5 10399303

[B68] Schmid M. Davison T. S. Henz S. R. Pape U. J. Demar M. Vingron M. (2005). A gene expression map of *Arabidopsis thaliana* development. Nat. Genet. 37, 501–506 10.1038/ng1543 15806101

[B69] Seino J. Ishii K. Nakano T. Ishida N. Tsujimoto M. Hashimoto Y. (2010). Characterization of rice nucleotide sugar transporters capable of transporting UDP-galactose and UDP-glucose. J. Biochem. 148, 35–46 10.1093/jb/mvq031 20305274

[B70] Sterling J. D. Atmodjo M. A. Inwood S. E. Kumar Kolli V. S. Quigley H. F. Hahn M. G. (2006). Functional identification of an *Arabidopsis pectin* biosynthetic homogalacturonan galacturonosyltransferase. Proc. Natl. Acad. Sci. U.S.A. 103, 5236–5241 10.1073/pnas.0600120103 16540543 PMC1458824

[B71] Tamura K. Peterson D. Peterson N. Stecher G. Nei M. Kumar S. (2011). MEGA5: molecular evolutionary genetics analysis using maximum likelihood, evolutionary distance, and maximum parsimony methods. Mol. Biol. Evol. 28, 2731–2739 10.1093/molbev/msr121 21546353 PMC3203626

[B72] Tan L. Eberhard S. Pattathil S. Warder C. Glushka J. Yuan C. (2013). An Arabidopsis cell wall proteoglycan consists of pectin and arabinoxylan covalently linked to an arabinogalactan protein. Plant Cell 25, 270–287 10.1105/tpc.112.107334 23371948 PMC3584541

[B73] Teleman A. Lundqvist J. Tjerneld F. Stålbrand H. Dahlman O. (2000). Characterization of acetylated 4-O-methylglucuronoxylan isolated from aspen employing 1H and 13C NMR spectroscopy. Carbohydr. Res. 329, 807–815 10.1016/S0008-6215(00)00249-4 11125823

[B74] Turner S. R. Somerville C. R. (1997). Collapsed xylem phenotype of Arabidopsis identifies mutants deficient in cellulose deposition in the secondary cell wall. Plant Cell 9, 689–701 10.1105/tpc.9.5.689 9165747 PMC156949

[B75] Urbanowicz B. R. Peña M. J. Ratnaparkhe S. Avci U. Backe J. Steet H. F. (2012). 4-O-methylation of glucuronic acid in Arabidopsis glucuronoxylan is catalyzed by a domain of unknown function family 579 protein. Proc. Natl. Acad. Sci. U.S.A. 109, 14253–14258 10.1073/pnas.1208097109 22893684 PMC3435161

[B76] Vuttipongchaikij S. Brocklehurst D. Steele-King C. Ashford D. A. Gomez L. D. Mcqueen-Mason S. J. (2012). Arabidopsis GT34 family contains five xyloglucan α-1, 6-xylosyltransferases. New Phytol 195, 585–595 10.1111/j.1469-8137.2012.04196.x 22670626

[B77] Wang Y. Alonso A. P. Wilkerson C. G. Keegstra K. (2012). Deep EST profiling of developing fenugreek endosperm to investigate galactomannan biosynthesis and its regulation. Plant Mol. Biol. 79, 243–258 10.1007/s11103-012-9909-y 22527750 PMC3349874

[B78] Ward J. M. (2001). Identification of novel families of membrane proteins from the model plant *Arabidopsis thaliana*. Bioinformatics 17, 560–563 10.1093/bioinformatics/17.6.560 11395435

[B79] Western T. L. Skinner D. J. Haughn G. W. (2000). Differentiation of mucilage secretory cells of the Arabidopsis seed coat. Plant Physiol. 122, 345–356 10.1104/pp.122.2.345 10677428 PMC58872

[B80] Wu A.-M. Hornblad E. Voxeur A. Gerber L. Rihouey C. Lerouge P. (2010). Analysis of the Arabidopsis IRX9/IRX9-L and IRX14/IRX14-L pairs of glycosyltransferase genes reveals critical contributions to biosynthesis of the hemicellulose glucuronoxylan. Plant Physiol. 153, 542–554 10.1104/pp.110.154971 20424005 PMC2879767

[B81] Wu A.-M. Rihouey C. Seveno M. Hörnblad E. Singh S. K. Matsunaga T. (2009). The Arabidopsis IRX10 and IRX10-LIKE glycosyltransferases are critical for glucuronoxylan biosynthesis during secondary cell wall formation. Plant J. 57, 718–731 10.1111/j.1365-313X.2008.03724.x 18980649

[B82] Xiong G. Cheng K. Pauly M. (2013). Xylan O-acetylation impacts xylem development and enzymatic recalcitrance as indicated by the Arabidopsis mutant tbl29. Mol. Plant. [Epub ahead of print]. 10.1093/mp/sst014 23340742

[B83] Yin J.-Y. Lin H.-X. Nie S.-P. Cui S. W. Xie M.-Y. (2012). Methylation and 2D NMR analysis of arabinoxylan from the seeds of Plantago asiatica L. 10.1016/j.carbpol.2012.02.025

[B84] Zhong R. Lee C. Ye Z.-H. (2010). Global analysis of direct targets of secondary wall NAC master switches in Arabidopsis. Mol. Plant 3, 1087–1103 10.1093/mp/ssq062 20935069

[B85] Zhong R. Lee C. Zhou J. Mccarthy R. L. Ye Z.-H. (2008). A battery of transcription factors involved in the regulation of secondary cell wall biosynthesis in Arabidopsis. Plant Cell 20, 2763–2782 10.1105/tpc.108.061325 18952777 PMC2590737

[B86] Zhong R. Peña M. J. Zhou G.-K. Nairn C. J. Wood-Jones A. Richardson E. A. (2005). Arabidopsis fragile fiber8, which encodes a putative glucuronyltransferase, is essential for normal secondary wall synthesis. Plant Cell 17, 3390–3408 10.1105/tpc.105.035501 16272433 PMC1315377

[B87] Zhong R. Richardson E. A. Ye Z.-H. (2007). Two NAC domain transcription factors, SND1 and NST1, function redundantly in regulation of secondary wall synthesis in fibers of Arabidopsis. Planta 225, 1603–1611 10.1007/s00425-007-0498-y 17333250

