# Supplementary material for: Discovery of diversity in xylan biosynthetic genes by transcriptional profiling of a heteroxylan containing mucilaginous tissue
Source: Front Plant Sci. 2013 Jun 7;4:183. doi: 10.3389/fpls.2013.00183 (PMC3675317; doi:10.3389/fpls.2013.00183)
Supplement: File S1 — Transmembrane domain predictions for PoIRX10_1, PoIRX10_3, and PoIRX10_4. [file DataSheet1.PDF]

Supplemental File S1. Transmembrane domain predictions for PoIRX10_1, PoIRX10_3, and PoIRX10_4

```
# PoIRX10_1 Length: 438
# PoIRX10_1 Number of predicted TMHs:  1
# PoIRX10_1 Exp number of AAs in TMHs: 20.50015
# PoIRX10_1 Exp number, first 60 AAs:  20.49092
# PoIRX10_1 Total prob of N-in:        0.99518
# PoIRX10_1 POSSIBLE N-term signal sequence
PoIRX10_1       TMHMM2.0        inside       1    12
PoIRX10_1       TMHMM2.0        TMhelix     13    35
PoIRX10_1       TMHMM2.0        outside     36   438
```

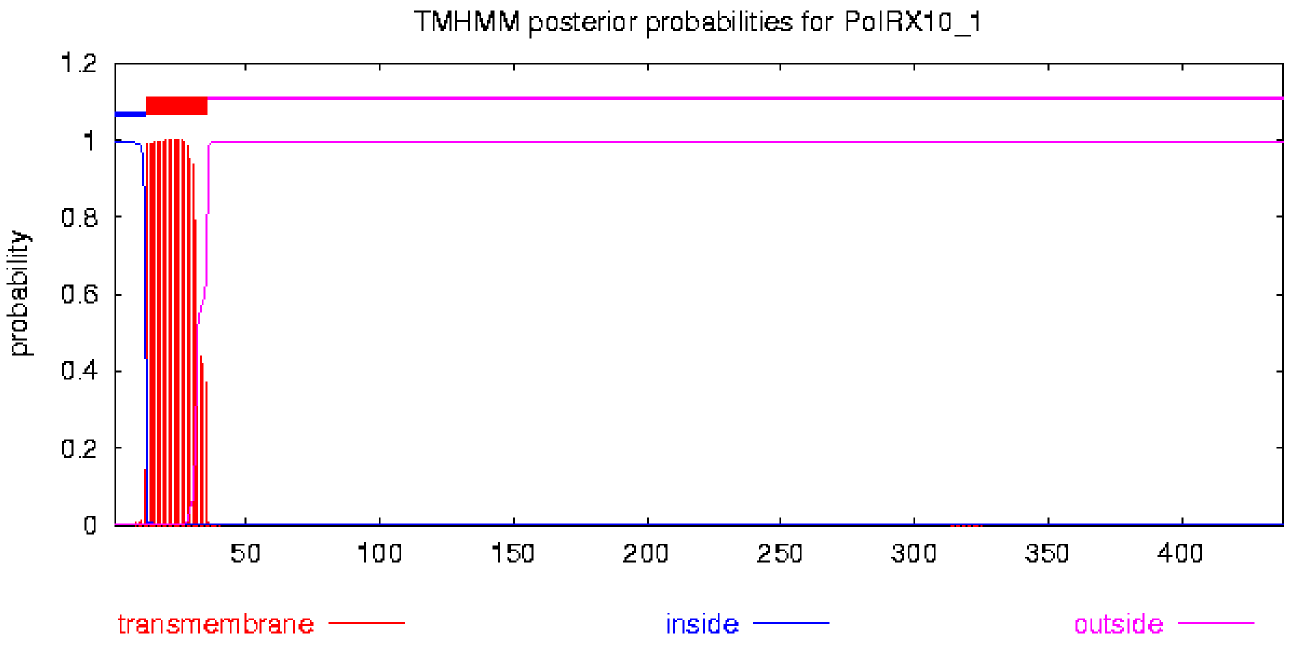

plot in postscript, script for making the plot in gnuplot, data for plot

---

```
# PoIRX10_3 Length: 416
# PoIRX10_3 Number of predicted TMHs:  0
# PoIRX10_3 Exp number of AAs in TMHs: 0.23649
# PoIRX10_3 Exp number, first 60 AAs:  0.21185
# PoIRX10_3 Total prob of N-in:        0.01805
PoIRX10_3       TMHMM2.0        outside      1   416
```

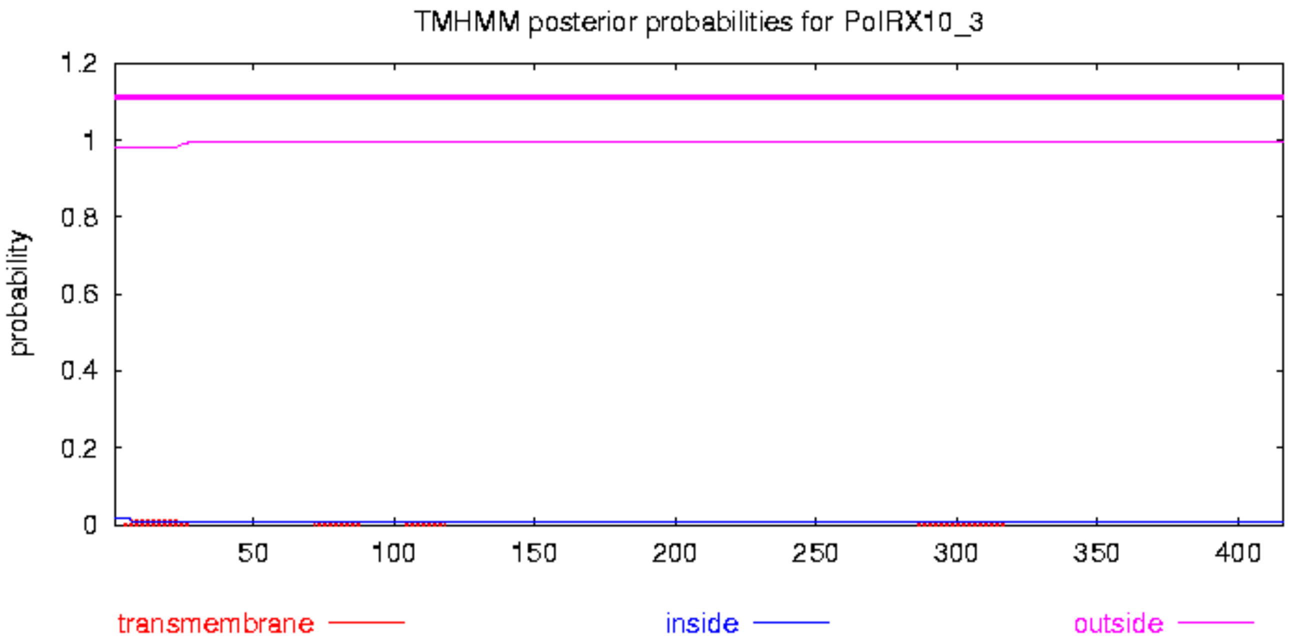

plot in postscript, script for making the plot in gnuplot, data for plot

---

```
# PoIRX10_4 Length: 425
# PoIRX10_4 Number of predicted TMHs:  0
# PoIRX10_4 Exp number of AAs in TMHs: 12.44177
# PoIRX10_4 Exp number, first 60 AAs:  12.26326
# PoIRX10_4 Total prob of N-in:        0.53197
# PoIRX10_4 POSSIBLE N-term signal sequence
PoIRX10_4       TMHMM2.0        outside      1   425
```

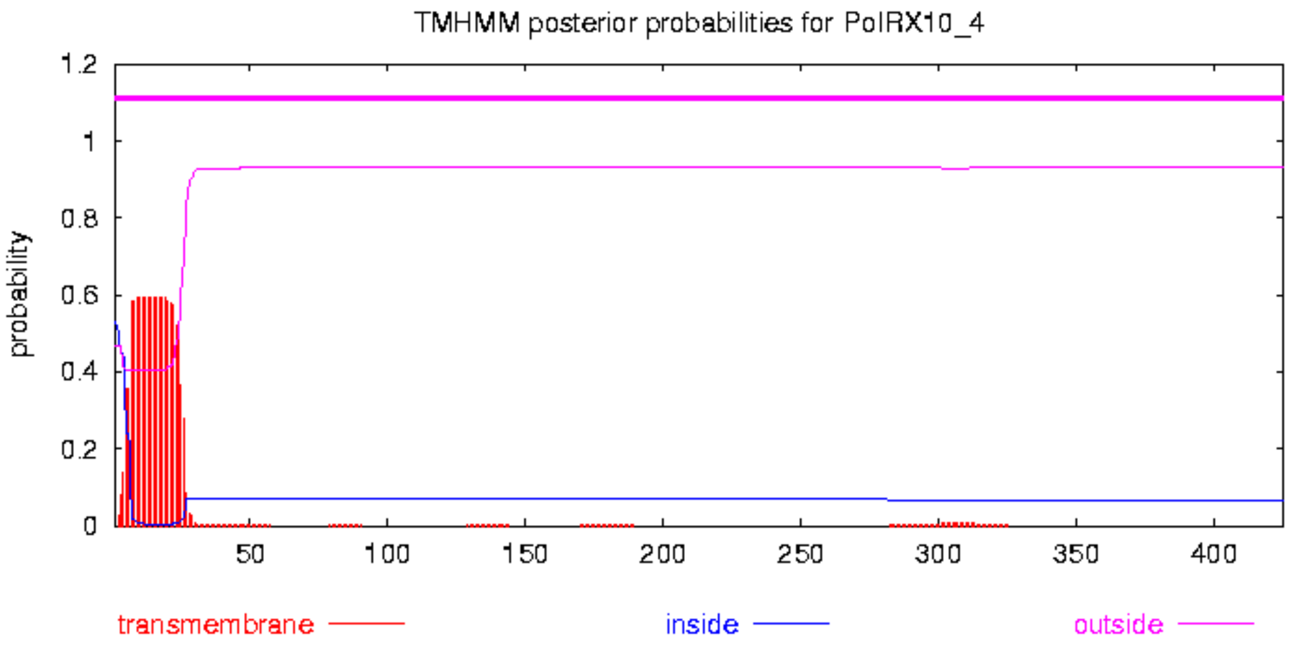

plot in postscript, script for making the plot in gnuplot, data for plot

---
