# Supplementary figures and images for: Discovery of diversity in xylan biosynthetic genes by transcriptional profiling of a heteroxylan containing mucilaginous tissue
Source: Front Plant Sci. 2013 Jun 7;4:183. doi: 10.3389/fpls.2013.00183 (PMC3675317; doi:10.3389/fpls.2013.00183)

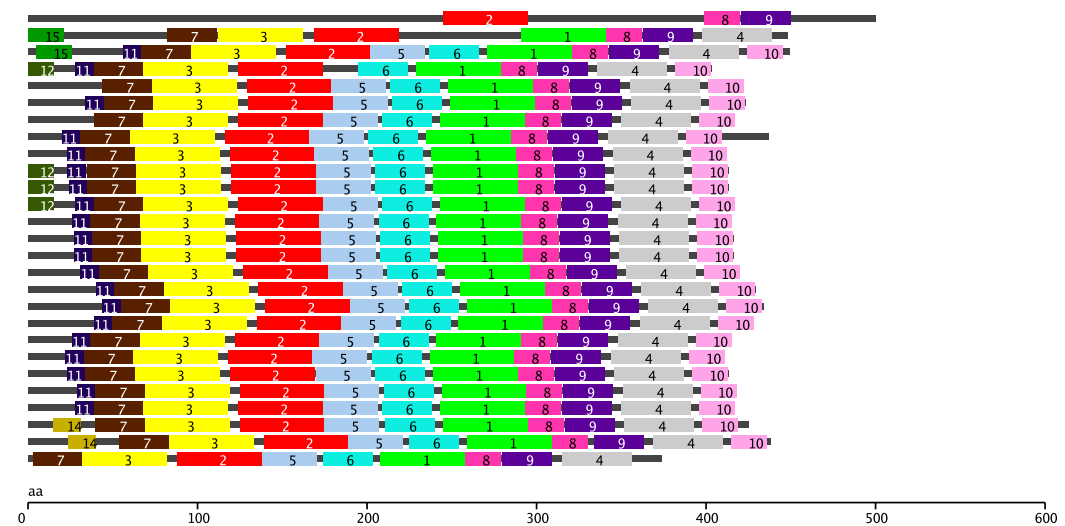

Supplement: File S3 — SALAD dendrogram and motif alignment of IRX10 proteins. [file DataSheet3.PDF]
