# Supplementary material for: Discovery of diversity in xylan biosynthetic genes by transcriptional profiling of a heteroxylan containing mucilaginous tissue
Source: Front Plant Sci. 2013 Jun 7;4:183. doi: 10.3389/fpls.2013.00183 (PMC3675317; doi:10.3389/fpls.2013.00183)
Supplement: File S4 — Protein sequence alignment of GT61 proteins. [file DataSheet4.PDF]

CLUSTAL 2.0.9 MULTIPLE SEQUENCE ALIGNMENT


```
PoGT61_7      ----------------------------------------------MDNEEANVATSKIGSIFAVRFTQ-----------------WLPGAFLLG------CIIM--LFTAVYT------------------------------------   43
PoGT61_4      ---------------------------------------------------MISVSNPVLVRSRCSRSELK------------GVSIMFWGLILC------LICSFSIWTN-LRTLSHHTSL----------------------------   52
PoGT61_3      ------------------------------------------------MYPPFVANLLKKRKDVITNLLRK------------KKDVLAQTLVLC------FIFALIIWVLSLLDGLTSVDN----------------------------   56
PoGT61_1      ----------------------------------------------MASVHKKKLKIYSGIFGGGLSRSQG------------KRLLRLCGVILC------FILLFSIWSDIFKTLLPTLSR----------------------------   58
PoGT61_6      ---------------------------------------------------MFKMDIHT-ILGG------C------------KRFLRKWRLILL------LFMSFSIWVTTVNRFMPTVSE----------------------------   46
PoGT61_2      ----------------------------------------------MVHETG-LGKLKKGHGYVRSFKKRY------------KLDKLQNGIFMG------CIVMGLIFFTVFKPYVETLLM----------------------------   57
PoGT61_5      -----------------------------------------------MYFRDVIANLLKRKKDVAT-LLKK------------KKDVIIKTLYLC------LILAFTIWVSSIPNSLTS-GN----------------------------   55
Bradi2g01420  -------------------------------------------------------------------MKGW------------AQRYLNLSFVAG------FLLVLLTYVVLSQPQRFAAISTTT-------------------------   40
Bradi2g13600  ------------------------------------------------------MVHHHR------ASLLLQHNNQQQQLRKQVDEEQQAEEQ----------------EGAGKTMKELRCRIVDYACHHRK------------------   56
Bradi2g01480  -----------------------------------------MEGGGKMGYG-GGGGQHETARLLKALSRTV------------EPRNFGIGLVAG------FLLVTCAYFSTANFDAIHIALMPS-------------------------   65
Bradi2g26590  ------------------------------------------------------MQQPQ------HHKPKPSLSGSSSWIRRSPPPSPPHKKPWG----------------AGGSRSRYACRFLP-------------------------   49
Bradi2g01380  -----------------------MKGGGVRFESQRFRLLTIVVGCFLISITFLLSSRPEAAAFDTLSPKAW------------LEEARTPAPKKP------AAIKTVVAAKSSSSSPAASLGRDFLVDITPQQQGDQSGASHGDGHGSST  109
Bradi2g61230  --------------------------MAASKLPEEKSLIGGAADNLQDGKKVGRLRWPGFVHFFFLLTVVTCAFVYAPRFFLAPSTSGVVASVDFFAPAKAAMSSSSPPPSSSSSTPQAALLPPPPSSSSNS------------------  106
Bradi2g25450  --------------MMKAGGDRPKLVRGLRQESRRFRLLVIVVGFFLVSLTFVVVSKPDAMLFGLNGKLPA------------DQTPTSILIQQK------VNAPPVSSRKTSTNTFGVDPK----------------------------   90
Bradi4g43180  ------------------------------------------------------MKFRSSARG--EPRR-F------------GNVALMALMLCS------LVALSLIRARFS--PIGSTG-----------------------------   44
Bradi4g27930  ------------------------------------------------------MKSGRNESSSKKLVSAG------------AAAWLLLPLLVLVVLKTDSLPQITLYSVDGVAPSPASDG----------------------------   56
Bradi4g27360  ---------MASTAYTRPSKPPGLAG-----------------------ERRPAARLSRELG-RIEPKKLG----------IGLVAGCCLALLTYISFARLFAIYSPVFES---TSLVMKNAPPAS------------------------   80
Bradi1g19160  ------------------------------------------------------MVQQQ----HRIYILQFDKGEPPEEEQLSCSPKQPAGATGRRIMYYYHDYGSKNGRQASAKSCSRPSRFLG-------------------------   67
Bradi1g06560  ---------MNSTAYSRPSKLPGGAGG---------------------ERR--PPRLMRGFAAKIEPKKLG----------AGLLAGCCLALLTYVSLAKLFAIYSPVFASTANTSGLLQNSPPSSSSV---------------------   87
Bradi1g34670  -----------------------------------------------------MMKAQQQGRSRQEPRR-M------------GNSAMVITMLLS------LCVLTFIKARYCSTPFPK-------------------------------   47
Bradi3g11300  ------------------------------------------------------MKSSNRNLSRGDGRR-L------------GNAALIAFMLGS------LLLLSLLRARFS--PIGKTG-----------------------------   46
Bradi3g03120  ------------------------------------------------------MKAS--LRSRQEPRR-V------------SNGVIIAAMLLS------LCVLSIVKARYCSTPFGKPDD----------------------------   47
Bradi3g11310  ---------------MKAV-ERAKLVRSLRQESRRLRLLVLVIGFFLVTLTFVVVSKPDALLFNLNGRLSV------------DQAPRSLLIRQR------VDAAT--DPQRS---AGQDPK----------------------------   83
AT2G03360     ------------------------------------------------------MVRKTT----------------------------------------------------SKSYSVA-------------------------------   13
AT2G03370     ------------------------------------------------------MVRKPS----------------------------------------------------SKIYSVAA------------------------------   14
AT2G41640     ------------------------------------------------------MVQYQR------LIIHHGRKEDKFRVSSAEESGGG----------------------GCCYSKRAKQKFR--------------------------   42
AT3G10320     ------------------------------------------------------MRQNLK------KVAQIKVDESKKLFPYVFRVKTS----------------------CGNCAKRSKPKLI--------------------------   42
AT3G18170     ------------------------------------------------------------------------------------------------------------------------------------------------------
AT3G18180     ---------------------------------------------------MTKKDILYDTVLARSFSKTD------------QKRLCCGAFIAS------LLLVLTLCTVVKPYLSPLPIV----------------------------   53
AT3G57380     ------------------------------------------------------MVQYKR------LIK----KGEKHRLSVEEGGSGASAVT----------------VSGGVYSKTAKQKLL--------------------------   44
AT5G55500     ----------------------------------------------------MSKR---------NPK------------------ILKIFLYMLLLNSLFLIIYFVFHSSSFS-PEQSQPPHIY-------------------------   45
Os08g0503800  ---------------------------------------------------MMPVRTYHHHHHHNNSNNHR-----------LRRIIPRVLLAVFAIYAVSFAAYLLRHQSPHPHPHPAADPERD-------------------------   63
Os01g0118400  ------------------------------------------------------------------------------------------------------------------------------------------------------
Os01g0118600  MLPNCHMGGHCAVAFQLPDHAAAELQERAARAFVAQQHGGGRAVAMKAAARERKPRHSNGRAAAAAAKNLSKVEPGRHLAVVRLFPACLLALLICLCVVKFFSSLSSQSQRIGTRSRMVSSWEGSAS-----------------------  127
Os01g0118700  --------------------------------------------------------MGGDHGKLMKSLKGA------------AQKYLGVGFLLG------FFLVLLTYFTVS--EQFAIAA----------------------------   46
Os01g0119000  ------------------------------------------------------------MGSEVKPAKLG------------LRRHLNAGFFAG------FLLVLLTYVIVS--QQFAMET----------------------------   42
Os01g0119100  -----------------------------------------MEGGGKAGYSYGGHHHHQDAKLLKNLSR-V------------EPRRFGLGLVAG------FLIVTCAYFSTAKFDAIHIAMISSPA-----------------------   67
Os01g0498300  ------------------------------------------------------MVQHHR------ATLLLHQ--QQRKGDATAEEGQQLVAE----------------EGAGK-MKELRRRLVDYACHHR-------------------   52
Os01g0956200  ---------------------------MASKLPD----LGGGGVEDGGGK-----KWPGFVQFFFVLSVVLCVLLYAPRFIVLTPTY---------------------------------------------------------------   51
Os02g0135500  ------------------------------------------------------MKQS--MRSRQEPRR-V------------SNGVIIAAMLLS------LCVLSIVKARYCSTPFVKPDD----------------------------   47
Os02g0327700  -------------------------------------MGSPKMAKSAMKQGIWRRRIGAPFAAVLVAAVLA------------VVVFSGQ-FAKGPNASSQFAPVQVDNTLRPTRDKPVSADQ-D-------------------------   74
Os02g0329800  ---------MTSTAYSRPSKLPGGGNG---------------------SDRRLPPRLMRGLTTKIEPKKLG----------VGLLAGCCLALLTYVSLAKLFAIYSPVFASTANTSALMQNSPPSS------------------------   86
Os02g0331200  ------------------------------------------------------MKS-NRNLTRGDGRR-L------------GNAALIAFMLGS------LLVLSLVRARFS--PIGKPG-----------------------------   45
Os04g0196600  -------------------------------------------------MRRGESKAATRGANRSSSSSSW------------KSRYIGYGLVLG------FVLVLL-YLMVN--AQFSNSPNAY-------------------------   55
Os05g0391600  ------------------------------------------------------MQQASSHQHHYQHSAKPPVSRSSSWIRRSPPPSPPHKKLWGG------------GGGGGGGRSRYVCRIVP-------------------------   59
Os06g0311000  ------------------------------------------------------MK-GRHERIKKGWSGSA------------AVWLLLVPLFVLIVLKTDFLPQVARLG-DTSFTKVADEM----------------------------   54
Os06g0470000  ---------MSSTAYTRPSKPPGPAG-----------------------ERRPP-RLAKELG-RIEPKKLG----------IGLVAGCCLALLTYISFARLFAIYSPVFES---TSLVMKNAPPAS------------------------   79
Os06g0475400  -------------------------------------MVS--QSKTPAPEPSTTKMPSAPPAVKGVAKTLA------------QHHKAVIGFLLG------FFLVLLLYTFLSG--QLVSSED-A-------------------------   65
Os06g0707000  --------------------------------------------------------MKAAVRSKKSKGSFC------------HPPLLLLIVAIQ------FLVIYSP-----TLDQYMVMLTTG-------------------------   46
Os06g0707200  ------------------------------------------------------MKHHHVARGRAEPRR-M------------GNAAMVITMLLS------LCVLTYIKARYCSTPFPK-------------------------------   46
Os07g0657400  ------------------------------------------------------MVQQQQQQQHRIYILQFRKGEQDQEVACKVS-TPPKAGTGRRVMYYYHDYG---GGGAGKNGKAAQPRAFS-------------------------   67
Os10g0492200  ------------------------------------------------------MKTRSSGRGGGEPRRRL------------RNAAVMALMLSS------LVALSLVRARFS--PIASDGG----------------------------   48
Os11g0575500  ------------------------------------------------------------------MRAALAVLVARRRHHLLRP-----------------------------------------------------------------   19
Os12g0238900  -------------------------------------------------RHAVGVGRPTAASLRSPAAADT------------LPDAGSAGRRNH------EAP----------SSPAFSSG----------------------------   45
Os02g0330200  ---------------MKPV-ERAKLVRSLRQESRRLRLLVLVIGFFLVTLTFVVISKPDALLFNLNGRLSV------------DHAPRSLLIRQR------IHADS--RRSADTFPAAEDPK----------------------------   86
Os03g0567600  ---------------MKAG-ERPKLVRGVRQESRRFRLLVIVVGFFLVSLTFVFVSKPDAILFSLNGKLPV------------EQAPTSILIQQK------VNEPSGESRKTSTDALRGDPK----------------------------   88
              1.......10........20........30........40........50........60........70........80........90.......100.......110.......120.......130.......140.......150
```

CLUSTAL 2.0.9 MULTIPLE SEQUENCE ALIGNMENT


```
PoGT61_7       -------------------------VFES----------------------------------------------------------------------YPVPLLKYSS-----------------------------------------     57
PoGT61_4       ---------IKSEDDMK-------------------------------------------------------------------------------------------------------------------------------------     60
PoGT61_3       ---------DVRTSFLT-------------------------------------------------------------------------------------------------------------------------------------     64
PoGT61_1       ---------SHFGFKMGNIAPARLLDNETSNVSELSENDNVN-----------------------------------------------------------DTKMSFQ-------PEDNS------------------------------     103
PoGT61_6       ---------AHLELEMD-IALDVRVSNERPNASQISENANVSYGNAT-----------------------------------EIKNPENNTTSTVNSHPTENANLSYGNATENENPENNTTS----------------------------     123
PoGT61_2       ---------LNRCPEMRTSTEKVSEAMESDPDSSVEDLNPIIRNISN-----------------------------------ELPDTIIEYELPDTIIEYSEPIMENVSSDPIMQNVSSSKS----------------------------     135
PoGT61_5       ---------NARTSFIT-------------------------------------------------------------------------------------------------------------------------------------     63
Bradi2g01420   -------SPNAVAKMAPRVTAAEHRKSAATEAPGETGEEKKEQMKPE---------------------------ASGNQDQDEKQQGQEEETWRTKDEDSAATVTATEEPRTKRDDRDVQ------------------------------     126
Bradi2g13600   ---------KHGHDALLLMLGGFAFFSCLLLLLPSSPFSAAMDE----------------------------------------LLQLGRNNRCDQEQARSDPPCAAV------------------------------------------     115
Bradi2g01480   ------KNGIGSPLTAFDGSGHRLGPDLGAQDPEDALSKQGSKAEVL-----------------------------DTDDEFSRKMNLPASSGPDASMGDTGKEGEAFPGAPPNATAEGG-------------GTLLPPLSSPEESTNST     167
Bradi2g26590   ----------LLALTVYSLLTVLRIPSSSLVVNTADSERVERREDVEA-------------------------------FKTHLPSNQNSLEARQETRSPASLPCSAFINGEAGG-RQS-------------------------------     126
Bradi2g01380   GGEKTTESTEWVKDTVIIQEVRGSSGGERAEPQEKRDRNAAAGAQDE------------AAAEEAVVRDDAVRSTTTTQTDVQPAGQEEVTPVGGRRMKLQGAAAEAVMTTEEQQTPASSE-----------------------------     218
Bradi2g61230   ---------SSTPQAALSPPPQPSSSSTNSTVRAGGGQDHADGGGGGG-------------------------------GDNASSSSSGDDEDLVLDNQVGSPCESMG------------------------------------------     174
Bradi2g25450   ----------VVDDEAYVKQKGLRAG-EEEDSRVLSEPDPASGMMEPKPIPNKDGKKGDKSDEEVLGG----ERKGEGEHG-HAEEKHRVTLPTVSNYTIHD-AEDNENAKQEG------------------------------------     187
Bradi4g43180   ----------AVDPKAVS--VVATEAAAAEASAVVPVEEEDKEEAFQ-----------------------------------PKVEHASDAAGDGAG-----------------------------------------------------     94
Bradi4g27930   ----------EKDVAVADSIKDSNREQQFLAVN----------------------------------------------GEVDVSSVNSDSKNEKHFLAMNGEKDASLINSDVAAPAMRSN-----------------------------     121
Bradi4g27360   --------------TAVPSTESGTVQQKIEL------EDEKDVVED-------------------------------------PKEPSFPEEERKVEEKE---EETAVTKPSGGGDAAET------------------------------     140
Bradi1g19160   ------------MRGFFGLLLICFLSVSTFLAAPGSS-SFFSGE----------------------------------------QQRAAAAAMAT-----AGAPCAAVR-----------------------------------------     118
Bradi1g06560   ---PETTDAIPAEATFVGRKNDDPAADPVDF------PEEGPSMDG-------------------------------------SQEPGLPEVVSRKEDDA---EKAIAATS-QPKPSEEDSAAAGAGEG---------------------     166
Bradi1g34670   --------------------------AAPVLEV-EVDEDYDGS------------------------------------------------RYRIDG-----------------------------------------------------     69
Bradi3g11300   ---------EAMKAEEQQ---GLRKGSAKIEET-VDDATAASTAAEE-----------------------------------EEEEIQPKATDTSTS-----------------------------------------------------     95
Bradi3g03120   ---------QLQEQMNSSIRMETDHPAATVEEEQEDEEDVEEAPATA-----------------------------------AKASTTSAAAAVVSG-----------------------------------------------------     100
Bradi3g11310   ----------VLDDD------ADQGTSVDEKRELTGEPEQGEKEQQE------------ATASELVGGGGREANKG--QEG--HQKYHKVTLPTVSNYTIHD-AEDSHNVKEEDSKTEEVQT----------------------------     172
AT2G03360      -------------LVIVMVIYVAFSSVFDRQLFRSLSFSQVSSV----------------------------------------GTLQQRWESRRTKQNPK--VMAAS------------------------------------------     66
AT2G03370      -------------AALVIVIYVVFCSVFDRQLLQSSSLYQ--------------------------------------------GTLQQPWESRRIKQAPQQPQETTS------------------------------------------     65
AT2G41640      ------------CLLFLSILSCCFVLSPYYLFG--FSTLSLLDS----------------------------------------FRREIE--GLSSYEPVITPLCSEI------------------------------------------     94
AT3G10320      ------------YLLIFSLISSCFVFAPQLLCFPYPSALFLIDS----------------------------------------SIKEIE--NRVSESNIESPKTSQK------------------------------------------     96
AT3G18170      ------------------------------------------------------------------------------------------------------------------------------------------------------
AT3G18180      ---------ELQLSVGTGLRMLSITELTTNTTISKEEVISECN-----------------------------------------------------------------------------------------------------------     87
AT3G57380      ------------LTIFLSLLSCCYIFS--------FSSFSLLGA----------------------------------------FSRESK--GFGPYELFIAPLCSGT------------------------------------------     90
AT5G55500      ----------HVSVN--------------NQSAIQKPWPILPSYLP------------------------------------WTPP-QRNLPTGSCEGYFGNGFTKRVDFLKPRIGGGGEG-----------------------------     105
Os08g0503800   ----------AVDAAGGGGGGGAVDRVRVEAPSSQKPWPRLPSFLP------------------------------------WTSASVRPPPKHSCEGYFGNGFSRLVDVLPARGGGGG-------------------------------     136
Os01g0118400   ------------------------------------------------------------------------------------------------------------------------------------------------------
Os01g0118600   ----TNVPRIPVAPLIMGRVDEDISTRSPELGSVFKNENFKNGTDS------------------------------------ENKSRSERQVAISTENDPPPGKEESLTKSPQTVSESEVPKPK--------------------------     211
Os01g0118700   --------PNAIRKTSPGHASPTIPPPVEEKRPQLPPIIEQRQAP---------------------------------KAEHEHAAVVQEKTPSAEEIEIQKETEEDHTKEKPTDDVTTT------------------------------     125
Os01g0119000   --------PTAVTSRAPRIDENES----VTKARVET-EKKREQE------------------------------------------------WQ-RPKDTSGAVSAEEFS--KRDSTNAK------------------------------     98
Os01g0119100   ------KNAAGFMNASSDGSNQQQ-LDLDR----DAMSREGSKAQVL-----------------------------DTDGD-DKISSLGPDLGHNASALEGKKKDETFAKDSGDASVSASTDEALAKDDDAIVGAVLPPLSS-EEPTNIT     175
Os01g0498300   ---------KHGHDALLRMLAGFALVSCLLLLLPGSPFSAAVDD----------------------------------------LLQMGR-TRLDDETP-PPPPCAAV------------------------------------------     109
Os01g0956200   ---------------GLDFFPQPPPNVTTSPARVVGDHNAG--------------------------------------------------EVVVLDNQLRSPCSSLA------------------------------------------     94
Os02g0135500   ---------QLQEQMNSSIRMETDEPATMAAGEQEEDEEEESSGGGG-----------------------------------AEPEVSATPAVVVTA-----------------------------------------------------     100
Os02g0327700   -------LERTVSSKLEGEDTEQIRLE-DGQSPNKEAAIEEQKPSQ-------------------------------AAAIDQDDNTLNPGLKQASGDERSAGGSDSLSKESPPQSQEGD------------------------------     155
Os02g0329800   ---PE-TGPIPPQETAAGAGNNDSTVDPVDL------PEDKSLVEAQP--------------------------QEPGFPSAESQEPGLPAALSRKEDDA---ERAAAAAASEIKQSEKKNGVAAGGD----------------------     175
Os02g0331200   ---------EAIKTEEQE---AMRKGSVKMETLEAADEAAASAAAAE-----------------------------------EEEETQPKAGDTTAG-----------------------------------------------------     95
Os04g0196600   -------LGPATTSKTESIPATTYQGNQAWQEDGSRGLEEGHREEVAS---------------------THTERSTGQRQEKDDESEKQRTEKNSIEEQLGNDRSSNYWEEGRQSEKKDT------------------------------     147
Os05g0391600   ----------LLVLTVYSIFTVVRIPSSSLVVSTADSERVERREDVEA-------------------------------FKTHLPSNQNNLEAREETRSPASLPCSALINGEAGGGQAA-------------------------------     137
Os06g0311000   ----------VQKVSSLGLDRARWQQQQTLDVAKLEDSVVGTSDELT--------------------------------GHVDANNEDSNQPNQ-QILAMSRSKDSRLINSDVAA-AKTSH-----------------------------     131
Os06g0470000   --------------TQQ---NPVLAQQQSKE------EDEKDVGED-------------------------------------ETDRKVPSFAETTEKNE---EEETVTKPSG--DEAEA------------------------------     134
Os06g0475400   -------IVRAVTQQS----TPAVHTDQDGRTTSPTSPTSTSSNTT-------------------------------QDNLEGKNTERSS--QPAVNDEASDKMEEDLIRQDIDQAGTKN------------------------------     141
Os06g0707000   --------KPGFPSMLIDG--RRSFKQVDE-----FIPEPHLRCDFR-----------------------------DNRSDVCEMEGAIRILG---------RTSEVFLVAPSLASISGG------------------------------     113
Os06g0707200   --------------------------AAEEMEVVEIDEDYDST------------------------------------------------RYKMTG-----------------------------------------------------     69
Os07g0657400   ------------MRRFFGLLLLSFVSVGTLFVAPVSFFSFVHSD----------------------------------------EGGGGAAAAARRAVEVAAAPCSGM------------------------------------------     123
Os10g0492200   ---------EALKLKADESKLVVTTNHAVVNTESGDSSSSSSSAAAE-----------------------------------EEEEVAAPAKPAAAG-----------------------------------------------------     101
Os11g0575500   -----------------------------------------------------------------------------------------------LEFQHRR------------------------------------------------     26
Os12g0238900   -----------VRETVTGVETNDDGAERGAEPAERDNAAPAAAAAIS------------GGDQEAQP------------------GTAAVDAAPGHDKSLRTAAATGTSPPRHEQ-PGETT-----------------------------     124
Os02g0330200   ----------VVDEDEGAEDATAKGTSEEEKRLLSSEPEQG-KNEEA------------ATASEVLGGGGEEDNKNGEEEG--HTQHSKVTLPTVSNYTIRD-AEDTDNGKQEDGKPNE-------------------------------     179
Os03g0567600   ----------VVDDEADAKPKGTGGGSEEEEGRVLSEPDPTSGMMEP--THNKDGN-GHKSHQETLGGGGDGESKGNDEEGEHAEQKHKVTLPTVSNYTIHDAAEDTENAKQEG------------------------------------     189
               .......160.......170.......180.......190.......200.......210.......220.......230.......240.......250.......260.......270.......280.......290.......300
```

CLUSTAL 2.0.9 MULTIPLE SEQUENCE ALIGNMENT


```
PoGT61_7      -----------------------------------------------------------------------------------------------------IGNQQ--------PIIHNFSNPRSDVYEMIGDIRLNGSSATILMVSNSE    98
PoGT61_4      ------------------------------------------------------------------------------------------------REP----------------LCRKL-PDGVDYCEIEGDIRIEANTSTIYVFTPDE    97
PoGT61_3      ---------------------------------------------------------------------------------------------SMNVSAIEV--------------TEKASCTSEHFCDLEGDIRVDAKSFTIYVMTHNE   107
PoGT61_1      --------------------------------------------------------------------------------------------ATFHEEEQ---------------LCRKVGPHYGDNCDFEGDIRVQANSATIYVFKPKQ   146
PoGT61_6      ----------------------------------------------------------------------------TINSHPTENANVSYRNATENENPENNTTSTINSHPTDLVLCRKVGPDYADYCDLDGDIRVEAITSTIYVVKPNE   197
PoGT61_2      ----------------------------------------------------------------------------DSSDQKSLRQPSKHKFRKSRSDLTDVDWRE--------PVIRNFSNKRTDVFEMIGDIRLNGSSGTILIATSNE   201
PoGT61_5      ---------------------------------------------------------------------------------------------SMNLSPIG----------------EKASCESEHFCELEGDVRVEAKSGTIYVMTHNE   104
Bradi2g01420  ----------------------------------------------------------------------------------------------LSEEA--------------KVVCNRDGPFS-DTCEVTGDVRINGSALSVTLLPSSR   167
Bradi2g13600  ---------------------------------------------------------------------------------------------------------------ADGTVCCDRTAERTDVCVMRGDIRTQAKSNSLFLLR-PA   153
Bradi2g01480  QEQGVLEDEELQVQDAMAAAAANASKKISGGSNGSSSPASVIQSDPATIPAPAQQIPTPQLEVKAPPVEQIPPTPVQQQEVKATPVQLIPPTPEVKQTGSSSSEAAAARRRPWKPLCDFASNRRIDWCELDGDVRVHGANGTVTLVD---   314
Bradi2g26590  ---------------------------------------------------------------------------------------------------------------GEGVLCCDRSHYRSDVCYLRGDARTDPSTSSVLLYNAPR   165
Bradi2g01380  -------------------------------------------------------------------------------------------TTARAADHHELQQQP-------LPTLCDFSDQRSDVCDFTGDIRMDANTSSFVLVVGQG   270
Bradi2g61230  ---------------------------------------------------------------------------------------------------------------KHGRICCDRSDFNTDVCFMSGDVRTHAASLSFLLFN---   210
Bradi2g25450  ---------------------------------------------------------------------------------------------TTNSQQGS-------------KPLCDFSNFRANVCEMRGDVRVHPRATS-ILFMEPD   230
Bradi4g43180  -------------------------------------------------------------------------------------------GGSSSS----------------SKPVCYETSRRSDTCEVAGDVRLVGRSQTIYVD----   133
Bradi4g27930  -------------------------------------------------------------------------------------------------------------------LSCNFSSVRMNICGMEGDVRIQGKASTVYVVSVSD   156
Bradi4g27360  ---------------------------------------------------------------------------------------------KIICDENG--------------VDEGFPYARPSVCELTGDIRISPREKTMFFVTPSA   183
Bradi1g19160  ---------------------------------------------------------------------------------------------------------------GNDTLCCDRTSERADMCFARGDVRMHSASSSFLLVSGNK   157
Bradi1g06560  -----------------------------------------------------------------------------------------TPPAKMTCDENG--------------VDEGFPYARPAVCELSGDIRVSPKDKTMYLVNPSG   213
Bradi1g34670  -------------------------------------------------------------------------------------------PIGEE-------------DFDPHRPTCYNTSKRSERCAAVGDIRFDGNHSKIYIN----   111
Bradi3g11300  -------------------------------------------------------------------------------------------GSSASVSTAAAVAN----HDQLPKPVCYETSRRSDTCEASGDVRVQGRTQTVHIR----   146
Bradi3g03120  -------------------------------------------------------------------------------------------AGKKSPKP--------KSKSKPKKQTCYMTSKRSERCEAAGDIRVEGNASLIHIN----   147
Bradi3g11310  ----------------------------------------------------------------------------KLEAPVDNSDGRSRQPAWDNVEWER-------------KPLCDFSNFRANVCEMRGNIKIHPNASS-VMYMEPA   232
AT2G03360     -----------------------------------------------------------------------------------------------------------------AKITCDRSHTSYDLCSINGSCILNPKTGTLTLMDRTL   103
AT2G03370     -----------------------------------------------------------------------------------------------------------------ATITCDRSHTNYDLCSINGSCNLDLKTGTLTLMD---    99
AT2G41640     ---------------------------------------------------------------------------------------------------------------SNGTICCDRTGLRSDICVMKGDVRTNSASSSIFLFTS--   131
AT3G10320     ---------------------------------------------------------------------------------------------------------------EE-SISCDRTGYRSDICFMKGDIRTHSPSSSIFLYTSND   134
AT3G18170     ------------------------------------------------------------------------------------------------MKP----------------ICT--KLARTEFCELNGDVRVHGKSATVSAAITFA    36
AT3G18180     ----------------------------------------------------------------------------------------------KMEKP----------------ICHCNTLGSKEFCDVSGDVRIHGKSATVLAAVTFA   127
AT3G57380     ---------------------------------------------------------------------------------------------------------------SNGTICCDRTGSRSDVCIMKGDVRTHSASSSVFLFTS--   127
AT5G55500     -----------------------------------------------------------------------------------------------------------------SWFRCFYSETLQSSICEGRNLRMVPDRIVMSRGGE--   140
Os08g0503800  -----------------------------------------------------------------------------------------------------------------GWFRCHHSETLRSSICEGGRVRLDPGLIAMSRGGE--   171
Os01g0118400  -------------------------------------------------------------------------------------------MNADSVDRRP---------------LCDFSDFRTDICDFSGDVRMAANTSEFVVVVDPA    44
Os01g0118600  --------------------------------------------------------------------------------------------SKISCDDKS--------------KDEGFPYARPIVCHLSGDVRVSPATSSVTLTMPLQ   255
Os01g0118700  ----------------------------------------------------------------------------------------------VEESAPA-----------KKPACDIQGPWASDVCSIDGDVRIHGAAHDVVIPPPIE   170
Os01g0119000  ----------------------------------------------------------------------------------------------PIENG--------------KVVCGSNGFYS-DTCDVDGDVRINGTALSVTLVPASR   139
Os01g0119100  QDS-VLEDEELKVQETAPATTNPSPEKSS--NNGSSP--SVVPSDPATLP--VQQIP-PTQEAKDPPAQQIPAVP----EAKVPPVQQIPTFPVVKTDS-----EAAPRRKEWKPLCDLWSNRRIDWCELDGDVRVAGANGTVSLVAPPG   308
Os01g0498300  ---------------------------------------------------------------------------------------------------------------SNGTICCDRTAMRTDVCIMRGDVRTEAASNSLFLLVPPP   148
Os01g0956200  ---------------------------------------------------------------------------------------------------------------GD-TICCDRSDFNTDVCFMAGDVRTDPSSLSLLLFPKQP   132
Os02g0135500  -------------------------------------------------------------------------------------------AAGGG-----------GGKRKP---TCRMTSKRSERCEARGDIRVEGNASTIYIG----   141
Os02g0327700  ----------------------------------------------------------------------------------------------GGTAESGAEPYIKCTAQSDIKICDLSNPRF-DICELCGDARTIGQSSTVVYVP---   207
Os02g0329800  --------------------------------------------------------------------------------------------TKIKCDENG--------------VDEGFPYARPSVCELYGDVRVSPKQKTIYVVNPSG   219
Os02g0331200  -------------------------------------------------------------------------------------------GGGGGASSAAVAAASTTATDHLTKPVCYESSRRSDTCEATGDVRVHGRSQTIHIS----   150
Os04g0196600  ----------------------------------------------------------------------------------------------IEFSEFGGGTDDFNNVANTKPICDTS-FGKYDICVLDGDTRAQGGGGAGAAVVTLV   202
Os05g0391600  ---------------------------------------------------------------------------------------------------------------AESALCCDRSHYRSDVCYLRGDVRTDPSTSSVLLYNAPR   176
Os06g0311000  -------------------------------------------------------------------------------------------------------------------LSCNFSSAHMDTCAMDGDIRIHGRSGVVYVVASSD   166
Os06g0470000  ---------------------------------------------------------------------------------------------TISCDENG--------------VDEGFPYARPPVCELTGDIRISPKEKTMFFVNPSS   177
Os06g0475400  ----------------------------------------------------------------------------------------------GTNHKPGAP---------RKPICDLLDPRY-DICEISGDARTMGTNRTILYVPPVG   187
Os06g0707000  ----------------------------------------------------------------------------------------------------------------------------------GGGVNATGVDAN--------   125
Os06g0707200  -------------------------------------------------------------------------------------------PIGEE-------------DFDPTRPTCYVTSKRSERCAAVGDIRVDGNHSKIYIN----   111
Os07g0657400  ---------------------------------------------------------------------------------------------------------------GNDSLCCDRTSTRADICFARGDVRMHSASASFQLVSSSS   162
Os10g0492200  -------------------------------------------------------------------------------------------GGG-------------------ARPVCYETSRRSDTCEAAGDVRVVGGSQTVYAD----   137
Os11g0575500  --------------------------------------------------------------------------------------------------------------------------------LLHGQRRADGP--VVPVAPAVP    46
Os12g0238900  -------------------------------------------------------------------------------------------TRADSVDQPR-------------HPLCDLSDHRTDVCDLAGDIRMDANASAFVVVVDPA   170
Os02g0330200  ----------------------------------------------------------------------------KYEFEMDADKGDNVEPETDNEEWNK-------------KPLCDFSNFRANVCEMRGNIRIHPNASS-VMYMEPA   239
Os03g0567600  ---------------------------------------------------------------------------------------------MNNVQQGS-------------KPLCDFSNFRANVCEMRGDVRIHPTATS-VLFMEPE   232
              .......310.......320.......330.......340.......350.......360.......370.......380.......390.......400.......410.......420.......430.......440.......450
```

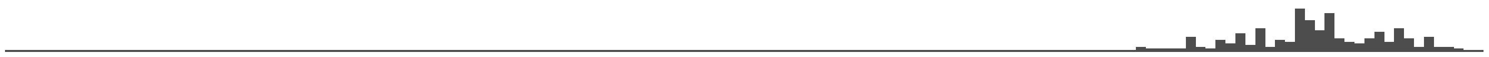

CLUSTAL 2.0.9 MULTIPLE SEQUENCE ALIGNMENT


```
PoGT61_7     ------NNMTP--VTVKPYARKADNCAMQNVREWKITAEANVAS----------------PRCSQT---ITTPAVVFSTAGYSGNHFHDFSDTLLPLYLTSRQFN--RSVLFLVTN---AQDWWISKHYTILDSLSSYN---ILDIDSQN  213
PoGT61_4     RLG--PNSTTS--WRTKPYIR---WYIQRNVKLWKVQIVRYSER------------ENYNVKCKHDKY-HTAPAILFSVGGQVGNYYHSFSDVIFPLYTTSYRFQ--RDVHFLASD---YQGWWINKFQDIFNVLTRHP---IVDIDNEC  219
PoGT61_3     MVS---HSANS--WVIRPYPR---FYLF-HT-QWIVKLMSYSEE------------EKISPKCTEK---HYSPAILFSTDEFSLNHYHWFTDVIFPLYMTSFGYT--PDIHFLVTD---YADWFVPKTHTILRRWSQYP---LIDIDNEK  224
PoGT61_1     ------NISKS--WNIKPYVR---WYAP-NVRNWTVKLIGGNDK--H---------KNLIPKCTHN---HNDPAILYSMSGYIVNYYHSFSDFLFPLYTVCFGFH--RDVHFLASD---FVWWLNGKFHEVIESLTRHQ---VIDIDNEN  262
PoGT61_6     ------IISKS--WTIKPYVR---WYAP-NIKNWTVKLMGLDDINNQ---------NNFIPACSIN---HSNPAILYSMGGFSGNIYHTFSDLLFPLYAISFGFN--REVHFLSTD---FVWWLNDKFEDIIVRLTKHQ---VVNIDEEY  315
PoGT61_2     ------QNIT-----VKPYARKEDGIAMGTIRAWEIINRTTDSM----------------PYCNQT---FSVPAVVFSIRGYGGNAFHAFSDLLIPLYLTCRQFN--GEVVFLTTD---MESWWLEKYKTFLDTVSKYD---IIDIDSQN  313
PoGT61_5     TIS---NSTDS--WIIRPYVR---WYIY-NVRHWTVKVVRYSEE------------DKNAPKCTRK---HYTPAILFSDEGFSLNHFHCYADVVFPLYMTSFGYR--PDIHLLITD---YRDWFVSKNHEILKRVSQYP---IMDIDKEN  222
Bradi2g01420 QE--TTSRRQQ--WKIRPYSRR----TMTDIREVTVTQLASADE------------ASAAPACTVTHE---VPAIVFALGGLTGNYFHDFSDALVPLFVASRRYG--GEVLFLVSN---IQPWWLAKYGAVVRRLSKYD---AVDLDKDN  286
Bradi2g13600 NSSSSGRAADE---RIRPYTRKWESSIMSTIDELRLRTVSEDS--------------SSSVPTPAR---CDV--------------QHEFNDGIIPLYITARRYN--KKVVFVMLE---YHDWWMTKYGHIVEQLSDYP---PVDFSGDN  261
Bradi2g01480 ----AAMAAEE--WRVKPYPRKADASAMRFVREITVRSTP-PNS------------ANAAPACTERHEG--VPALVFSDRGYTGNYFHAYTDVILPLFLTARQYS--GEVQFMVSD---FQMWWIGKFMPVFKSLSNYP---LIDLAADS  435
Bradi2g26590 GAAP---------EKVRPYTRKFEGSIMSTIDEVTILPVPD-GNGTSDAASRD--RDSLRRRCDVRHP-AGVPAVVFSTGGYTGNVYHEFSDGLIPLFITSQRFA--GEVVFVVLE---YHYWWLGRYGAILERLTNYK---IVDFRYDR  294
Bradi2g01380 --T--SPQ--S--HKVRPYPRKGDETCMGRVTEINVRTTTTASS--------------PPPLCTQTHD---APAVTFSIGGYTGNIFHDFSDVIVPLFNTVHKYA--GDVQLVMAN---VAPWWLIKYDKLLRSISRYA---PLDLAAAG  387
Bradi2g61230 -ANATVEE-----QRIRPYTRKWERHLMASIHEVRLRAPTA----------------SESETSKCDVV-HEAPALVMTAGGYTGNLFHAFNDGFLPAWLTSSHLR--HGVVLAVLA---YNPWWAGTFRELISELSGRRG--VVDLVHDT  330
Bradi2g25450 --G--SQRDEV--WKIKPYPRKGDEFCLSHITELTVKSSK------------------VAAECTKYHD---VPAVIFSLTGYTGNLFHDFTDVLVPLFTTASEFN--GEVQFLITD---MALWWTIKYQTVLQKLSKYP---LIDFSKD-  344
Bradi4g43180 ------VLKQE--WKTKPYCRKHDTFALSHVKEWSLK--PAGDG-------------SAVPECTSNSS---ATAFVLSTGGFTGNPFHDYTDVLIPAFISAHRFA--GEVQFLVSS---YKPWWMNKYIQIFQQMSRYE---VIDIDAD-  248
Bradi4g27930 YRP----ENST--VIIRPYPRKWEIPTME---------------------------------------------------------------------------------LLLATN---YDRKWIAKFRHVLGALSSYP---VIDLDADA  213
Bradi4g27360 AGAAALDANGE--KKIRPYARK-DTFLLPGVVEVTIKSVPSAEA---------------APACTRQHD---VPAVVFSVAGYTDNFFHDNTDVMIPLFLTTAHLR--GEVQLLITN---FKPWWVKKFTPLLKKLSNYE---VINFDKDE  304
Bradi1g19160 ESSPAPGKKEEQEERIRPYTRKWEANVMATIDEVRIRRVHPAHGGP------------SAPRCDVV---HDVPAVLLSTGGFTGNVYHEFNDGLIPMFVTAAHLR--RRVVFVILE---YHDWWITKYGDVVSRLSAFP---PIDFSADR  284
Bradi1g06560 AAA-GFDENGE--KRLRPYARK-DEFLLPAVVEVTVKSVPSASG---------------APRCTKRHR---VPAVVFSVAGYTDNFFHDNTDALIPLFLTTAHLK--GEVQLLITN---YKPWWVQKYTPVLRKLSNYD---VINFDDED  333
Bradi1g34670 ------PLDKE--WRTKPYARRHDAVAMDDVREFTLLPFDTESSNTT----------VVPPLCTRNHS---VPAFLFSSGGFAGNLYHDYTDVLVPLFTSTHHFR--GEVQFLLTD---IKDWWLDKFTPLFRQLSNYD---VIDADND-  231
Bradi3g11300 ------PLEKE--WKVKPYCRKHDAFAQSHVKEWTLRPLSSSS-----------------PHCTVNSSGSGLTAFVLSTGGFTGNLFHDYTDVLIPAFITSRRFH--GEVQFLVSS---YKSWWTSRYLQIFQQLSNYE---VLDIDAD-  262
Bradi3g03120 ------PLSKS--WKTKPYARYHDPVAMAHVREFTLKPFS---SSSS----------PPPPACTKNHS---VPGFLFSNGGFSGNLYHDYTDVLIPLFLTTRSFR--GEVRFLLSG---LKPWWVTKFTPLFRQLTNYD---VLDVDND-  264
Bradi3g11310 --G--SKRDEQ--WKIKPYPRKGDELCLSHITELTVKSSK------------------VAPECTKYHN---VPAVVFALTGYTGNLFHDFTDVLVPLFTTASEFN--GEVQFLITD---MAIWWTRKYHVVFEKLSKYP---LIDFNKD-  346
AT2G03360    TTSAPLV------EKIRPYPRKSENWIMPRIRELKLTSGPS----------------DLTRSCDIT---HDSPAIVFSAGGYTGSIYHDFIDGFIPLFITANSVYPDRDFILVVVN---PKEWWMPKYIDILGTFSKHK-TILLDKENAS  224
AT2G03370    PTSAPLV------EKIRPYPKKADNWIMPRIRELTLTSGPL----------------GLPRSCDIT---HDLPAIVFSAGGYTGSIYHDLMDGFIPLFITANSVYPDRDFIPVVVN---AKEWWMPKYIDILGTFSKHKPILLLDKESVA  221
AT2G41640    STNNNTK--PE---KIKPYTRKWETSVMDTVQELNLITKDSNK--------------SSDRVCDVY---HDVPAVFFSTGGYTGNVYHEFNDGIIPLFITSQHYN--KKVVFVIVE---YHDWWEMKYGDVVSQLSDYP---LVDFNGDT  251
AT3G10320    LTTDQVL--QE---KIKPYTRKWETSIMETIPELKLVTKDMKLFG-------------DKRKCEVI---HEVPAVLFSTGGYTGNLYHEFNDGLIPLYITSKRFN--KKVVFVIAE---YHKWWEMKYGDVLSQLSDYS---LIDFNKDK  255
AT3G18170    FSG-----NST--WHIRPYARKGDTVAMKRVREWTVKLEQNADQL-E---------NANFSRCVRN---HSVPAMIFSLGGYSMNNFHDFTDIVIPLYTTARRFN--GEVQFLVTN---KSPSWINKFKELVRKLSNYE---VIYIDEED  158
AT3G18180    FSG-----NST--WYMRPYARKDQVPAMKRVREWTVKLVQ----------------NASLSRCVRN---HSVPAILFSLGGFSLNNFHDFTDIVIPLYTTARRFS--GEVQFLVTN---KNLLWINKFKELVRKLSNYE---VIYIDEED  243
AT3G57380    LKNKTKI--TK---KIKPYTRKWETSVMQTVQELNLVYRDEENNSLV--------VSSVNDICDVF---YNVPAVFFSTGGYTGNVYHEFNDGIIPLFITSHHFN--KKVVFVIVE---YHSWWIMKYGDIVSQLSDYP---PVDFNGDK  253
AT5G55500    --------------KLEEVMGRKEEEELPAFRQGAFEVAEEVSSRLGFKRHRRFGGGEGGSAVSRRLVNDEMLNEYMQEGGIDRHTMRDLVASIRAVDTNDFVCEEWVEEPTLLVT----RFEYANLFHTVTDWYSAYVSSRVTGLPNRP  272
Os08g0503800 --------------PLDQVMGRAEEEELPKYEPGALQVEAAAKR-------------------TGPLVEAGFLDAYVPTGGIGMHTMRSLLDSGRVVPPGELHCSQWVEEPTLLVT----RFEYANLFHTITDWYSAYVSSRVTDLPNRP  284
Os01g0118400 --A--AAV--S--HKVRPYPRKGDATCMGRVTEITVRATGDAAG---------------APRCTRAHD---VPAVAFSIGGYTGNIFHDFSDVIVPLYNTVQRYR--GGVQLVMVN---VASWWLVKYDKLLRELSRHA---PIDLAKAG  160
Os01g0118600 QG-----EAAA--RRIRPYARR-DDFLLPLVREVAITSAASEGD---------------APSCNVSHG---VPAVIFSIGGYTGNFFHDMADVLVPLYLTTFHFK--GKVQLFVAN---YKQWWIQKYKPVLRRLSHRA---VVDFDSDG  371
Os01g0118700 GGG-SNPNPRE--WRVVPYSRK----HMGGLKEVAVREVAS---------------AAEAPACDVRSP---VPALVFAMGGLTGNYWHDFSDVLIPLYLQARRFD--GEVQLVVEN---IQMWYVGKYKRVLDRLSRHD---IVDMDRDD  287
Os01g0119000 R----SERRRE--WKIQPYPRR----TVSGIAEVTVTRQQ---D------------RAAAPACTVTHG---VPGVVFALGGLTGNYWHDFSDVLVPLFVASRRYG--GEVQFLVSN---IQPWWLGKYEAVVRRLSRYD---AVDLDRDT  253
Os01g0119100 PADERTFRAES--WHIKPYPRKADPNAMRHVRVLTVQSLPAPAA------------SAAAPACTERHD---VPGLVFSDRGYTGNYFHAYTDVILPLFLTARQYS--GEVKLLVSD---FQMWWLGKFLPVFKAVSNYD---LINLDDDR  433
Os01g0498300 DNSTAAAGRDE---RIRPYTRKWESSIMSTIDELRLRAVPEGG--------------AAPASCDVR---HDVPAVVFSTGGYTGNVYHEFNDGIIPLYITARQYN--KKVVFVMLE---YHDWWMTKYGHIVEQLSDYA---PIDFTNDR  270
Os01g0956200 PAANATVE-----ERIRPYTRKWEALIMSRVEEVRLRMAPP----------------EEEPGHRCDVR-HDAPLLVMTAGGYTGNLFHAFSDGFVPAWLTVQHLR--RRVVLGVLS---YNPWWAGTYGEIISGLSDYH---VVDLLHDK  252
Os02g0135500 ------GIDKE--WKTKPYARYHDPVAMAVVREFTLKPVT---ESS--------------PACTRNHS---VPAFVFSNGGFSGNLYHDYTDVLVPLFLSTHQFK--GQVQFLLSG---LKPWWVNKFNLFFRQLTKYD---ILDIDND-  254
Os02g0327700 -QN-RASNGEE--WIIRAQSRK----HLPWIKKVTIKSVNS---------------SEPEPICTSKHH---IPAIVFALGGLTANVWHDFSDVLVPLFLTARQFN--RDVQLIITN---NQPWFIKKYSAIFSRLTRHE---IIDFDSDG  323
Os02g0329800 AG--GFDENGE--KRLRPYARK-DDFLLPGVVEVTIKSVPSEAA---------------APKCTKQHA---VPAVVFSVAGYTDNFFHDMTDAMIPLFLTTAHLK--GEVQILITN---YKPWWVQKYTPLLRKLSNYD---VINFDEDA  338
Os02g0331200 ------PLEQE--WKVKPYCRKHDAFALSHVKEWALRPLSTADT------------YPAVPHCTVNSS---ATAFVLSTGGFTGNPFHDYTDVLIPAFITAHRFA--GEVQFLVSS---FKSWWTNRYMQIFQRLSKYE---LVDIDND-  268
Os04g0196600 SP---RAAPRE--WKIKPYSRK----YLDGLKPVTVRSVPN---------------PEDAPPCTTRLN---VPAMVIELGGLTGNYWHDFTDVLVPLFIGARRFG--GEVQLLVVN---LLPFWVDKYRRIFSQISRHD---IVDLEKDD  317
Os05g0391600 GSAP---------EKVRPYTRKFEGSIMSTIDEVTIVPVVDAGSGSNGTTAGDTGKDSLRRRCDVRHP-PGVPAVVFSTGGYTGNVYHEFSDGLIPLFITAQRFA--GEVVFVVLE---YHYWWLGRYGAVLERLTNYK---VVDFRYDR  308
Os06g0311000 YRP----ENAT--AVIRPYPRKWEQATMERVRQITIRSTAPPGAAVADTDGGG---AIIPLRCTVARD---MPAVVFSTGGYSVNFFHTMNDILLPLYITAREHG--GRVQLLAAN---YDRRWTAKYQHALAALSMYP---VVDLDADA  296
Os06g0470000 AG--AFDGNGE--KKIRPYARK-DDFLLPGVVEVIIKSVSSPAI---------------APACTRTHN---VPAVVFSVAGYTDNFFHDNTDVMIPLFLTTSHLA--GEVQFLITN---FKPWWVHKFTPLLKKLSNYG---VINFDKDD  296
Os06g0475400 ERG-LADDSHE--WSIRDQSRK----YLEYINKVTVRSLD----------------AQAAPGCTSRHA---VPAVVFAMNGLTSNPWHDFSDVLIPLFITTRVYE--GEVQFLVSD---LQPWFVDKYRLILTNLSRYD---IVDFNQDS  303
Os06g0707000 --------ATR--WKIQPYTRKGESRVMPGITEVTVRLVT----------------ADEAPPCDEWHD---VPAIVYSNGGYCGNYYHDFNDNIIPLFITSRHLA--GEVQLLVTQ---KQRWWFGKYREIVEGLTKYE---PVDLDAEQ  238
Os06g0707200 ------PLDKE--WRTKPYARLHDAVAMDDVREFTLVPFG--GANHT----------AVPPLCTRNHS---VPAFLFSSGGFAGNLYHDYTDVLVPLFTSTNHFG--GEVQFLLSG---IKDWWLDKFTPLFRQLSRYD---VIDVDND-  229
Os07g0657400 GNATAAAVEEE---RIRPYTRKWEANVMATIDEVRLRRVP--AGG--------------AARCDVV---HDVPAVVFSTGGYTGNVYHEFNDGILPLFVTSNHLR--RRVVFVILE---YHDWWMTKYGDVVSRLSAFP---PIDFTADR  282
Os10g0492200 ------TLDRE--WKTKPYCRKHDAFALSHVKEWTLRPLPSGDGGGGG--------AAVAPRCTTNST---ATAFVLSTGGFTGNPFHDYTDVLIPAFITAHRFG--GEVQFLVSS---YKSWWMNKYIQIFQQMSRHD---VVDVDADG  260
Os11g0575500 QAAADGER-----HRGGEDTSVHAQVGGAHHEQGRGGAAPD----------------GSS-------R-HDAPLLVMTAGGYTGNLFHAFSDGFVPAWLTVQHLR--RRVVLGVLL---YNPWWAGTYGEIISGLLDYH---VVDLLHDK  159
Os12g0238900 --V--GADGPT--YKVRPYPRKGDATSMGRVTEITVRTT---------------------------------AAVVFSISGYTGNLFHDFTDVIVPLYNTAARYC--GDVQLVVTDGNAATRRWLARYGAVLRGLSRHV---PLDLAAEA  276
Os02g0330200 --S--SKREEI--WKVKPYPRKGDELCLGHITEITVKSSK------------------VAPECSKYHN---VPAVVFALTGYTGNLFHDFTDVLVPLFTTASEFN--GEVQFLITD---MAIWWTRKYKVVFDKLSKYP---LIDFNND-  353
Os03g0567600 --G--SQRDEV--WKIKPYPRKGDEFCLSHITEVTVKSSK------------------VAPECTKYHD---VPAVIFSLTGYTGNLFHDFTDVLVPLFTTASEFN--GEVQFLITD---MALWWTIKYQTVLQKLSKYP---VIDFSKD-  346
             .......460.......470.......480.......490.......500.......510.......520.......530.......540.......550.......560.......570.......580.......590.......600
```

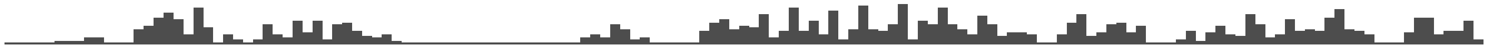

CLUSTAL 2.0.9 MULTIPLE SEQUENCE ALIGNMENT


```
                       *                                                                                              *            . :
PoGT61_7       -----EVLCFS-RVIIGLKADKDLRADHPSPP----HYSMLDFAQFLRNVYSLGRESVKDF----H----G----------------------------FRLPRMLIISRQHS--RRINNEGDVVDMARGVGFDVIAEEMG---------  306
PoGT61_4       ----WEVHCYK-KMVVGLKYHVDLVVDDRSVSEYSTGVSMQNFRHLLRDAYSLERKTSIS---RQR----G----------------------------STSPRLMIVSRKKT--RVILNQEEISQVAREVGFEVVLADDV-S-------  319
PoGT61_3       ----ERVHCFR-KLFVGLKFHGDLIVN-KSSPEYAAGVSMQKFRRFLRDTYSLERQIAIEP--RLV----N----------------------------VTRPRLMIMSRKSS--RILLNEDEISRVANEVGFNVVITDGE-Q-------  324
PoGT61_1       ----GQVHCYP-KIVAGLK-NPNLVIE-QSEQKYETGASMLNFKKLIRRAYSLPRQKAMESI-QGQ----K----------------------------PPIPHLLIISRKRT--RVLTNEGKISEAANELGFEVVTADP--D-------  361
PoGT61_6       ----GQVHCYT-KMVAGLK-HPNLVID-KSEPEYERGASMLNFKDIIREVYSLQRKMAME----------N----------------------------RSIPRLIFISRKKT--RVITNQEEISQVASELGFKVVIADP--E-------  409
PoGT61_2       -----EVLCFP-RAFVGLKANKDFSIDPLQPP----YYFMLDFTQFLRKVYSLKRDYVDDL----D----D----------------------------VRKPRMLLISRQTS--RRILNEGEASDVARAVGFDVVVDEIG---------  406
PoGT61_5       ----EQVHCYQ-KMFVGLKFFGDFLVN-KSIPEYAAGMSIDNFRQFLRDTYSLERQTAIRP--SLV----N----------------------------LTRPRLMIVSRKSS--RILLNEDEISRVAKEVGFNVISTDSG-L-------  322
Bradi2g01420   Q-----TRCFR-HVSVGLRLTKEFDIA---AGKNN-PLSMPDFTAFLRETYSLPRNSPTKISLGATGSNDD----------------------------NQKPRLMLIHRSHY--RKLLNVPEIVAAAESAGFAVTISDPR---------  387
Bradi2g13600   R-----THCFR-EAVVGLRIHDELAIDASRMMPP---GSIADFRQMLDDAHRGRVQVIIDEEAEAAKNSSSSNSK-TKKPADDEDE--------------DKPRLVIVSRNGS--RAIENEAELARAAAGAGFRVELLQPRPD-------  378
Bradi2g01480   R-----VHCFK-HVQVGLTCHADFSIDP--SRSPN-GYSMVDFTKFMRQTYKLPRDLAAPING-------------------------------------ARPRLLIIARART--RRFDNLAEIVRGAEKVGFEAVVSEG----------  527
Bradi2g26590   R-----VHCFS-EMIVGLRIHGELVVDPKLMPNG---KSIKDFQALLHQGYSKAP-----ASSSSAPVPLPLVP-LSRPCLD-HATTK-----------AAKPKMLIFIRKQN--RVILNLPHVVTACRRAGFAPHVMNLRRQ-------  408
Bradi2g01380   TRG--EVHCFR-HAVVSLRAHRELIIEKDRSLD---GLATPDFTRFLCSALSLPRDAPTNIADGSG----------------------------------RKPRLLIISRHRT--RILLNLAAVVLAAEEAGFEAVVNESDVA-------  488
Bradi2g61230   R-----THCFPAGAIVGSRFHGVLSVDPARTRDH---KSLLDFHTFLARAYEADNAALKQE---EQQGR--------------------------------RPRLGILARKGN--RVIENQGAVARLAESIGFEVSILETANG-------  428
Bradi2g25450   ----DQVHCFK-HAIVGTHAYMEFTIDSSKAPH---GLTMVDFNRFMRGAYSLGKDTAVVLGETPK----------------------------------VKPRLLIIKRHRT--RMFLNLEEIITMAEELGFEVVIDEANVS-------  443
Bradi4g43180   ----DEVRCYR-SVVVGPTFHKELGVDPS---S---GISVVDFRKMLRNAFGLERATATPSGDRWD----I----------------------------RRRPRLLIISRRASRGRAFMNERAMADMAGSLGFDVRIGDPDTG-------  348
Bradi4g27930   A-----VRCFP-SAHVGIESHMELSIDP--ALS-FHGNTMMDFRDFLRSAYSLKRSWTTPVSRNNSS-SSS----------------------------SRKPRLVILLRRHS--RAMTNEGDAMAAATEAGFEVVPAGPE---------  314
Bradi4g27360   E-----VRCFR-QGNLGLYRDRDLILSPHPTRNPR-NYTMVDYNRFLRGAFGLPRDAPAVLGEKTS----------------------------------ARPKMLMIERKGT--RKLLNLAAVVAMCEELGFAVTVAEAG---------  402
Bradi1g19160   R-----VHCFP-ELIAGLRIHGELTVDPARTPDGA--TSIADFRALLDDAYRGRLLYLDRLA--AAR-KHRRRPRRRSAINSVEIE--------------KRPRLTIVSRTGS--RVIENEEAVVSLASEIGFEVRVIRPERS-------  400
Bradi1g06560   GGA---VHCFP-DGYLGLYRDRDLIISPHPTRNPR-NYTMVDYNKFLRGALELPREKPAVLGEEPG----------------------------------MRPRMLIISRSGT--RRLLNLDEVSAAASELGFNVTVAEAGG--------  434
Bradi1g34670   ----QQVHCFR-RIIIGATFHRAMGIDPKRSPG---GETVADFKRLLRHAFHLTRPVAS----------------------------------------RDNPRLLIISRKSS--RRFLNERAMAHAAALAKFDVRIAEPD-N-------  323
Bradi3g11300   ----DEVRCYD-KAVVGPTFHKELGVDASKTPSP--GYSMVDFRAMLRQAFGLERAAAEPSGDRWD----I----------------------------RRRPRLLIISRKHT--RAFLNERAMADMAMSLGFDVRVGEPD-V-------  363
Bradi3g03120   ----GEIHCFP-RIVVGSTFHKDMGVDPSKSPG---GVSVVDFKRTLRAAFDLPRASASRAGARG----------------------------------DGKPRLLIISRKSS--RRFLNEKEMAAAGAAMGFQVRIAEPD-Q-------  362
Bradi3g11310   ----DQVHCFN-HAIVGLHAYMEFTIDSSKAPH---NYSMVDFNRFMRQTYSLPRDAVSALGEIPK----------------------------------TKPRLLIISRQRT--RMFLNLQEVVAMAEELGFEVVVEEANVS-------  445
AT2G03360      I-----THCFT-SATVGLISHGPMTIDPTQIPNS---KSLVDFHNLLDKALNPNLSIIK----------------------------------------INKPRLILVRRYGNIGRVILNEEEIREMLEDVGFEVITFRPSKT-------  318
AT2G03370      T-----THCFT-SAIVGLITHWPMTIDPTQIPNS---KSLVDFHNLLEKAFTTNISTPK----------------------------------------THKPRLMLVSRYGNIGRVILNEQEIKEMLEDVGFEVIIFRPSKT-------  315
AT2G41640      R-----THCFK-EATVGLRIHDELTVNSSLVIGN---QTIVDFRNVLDRGYSHRIQSLT-QEE--TEANVT---------ALDFK---------------KKPKLVILSRNGSS-RAILNENLLVELAEKTGFNVEVLRPQKT-------  357
AT3G10320      R-----THCFK-EAIVGLRIHGELTVDPSQMQDDG--TTINEFRNVLDRAYRPRINRLDRLEEQRFHARLA---------QRRKA---------------KRPKLALFSRTGS--RGITNEDLMVKMAQRIGFDIEVLRPDRT-------  364
AT3G18170      -----ETHCFS-SVTVGLTRHREYFKELTIDPSNSE-YSMSDFRSFLRDTYSLRNDAVATR---QI----R----------------------------RRRPRILILARGRS--RAFVNTGEIARAARQIGFKVVVAEA----------  254
AT3G18180      -----ETHCFS-SVIVGLNRHRDYDKELTTDPSNSE-YSMSDFRKFLRDTYSLRNSAVTT---------------------------------------RRKPRILILSRSRS--RAFVNAGEIARAARQIGFKVVVAEA----------  335
AT3G57380      R-----THCFK-EAIVGLKIHDELTVESSLMLGN---KTILDFRNVLDQAYWPRIHGLI-QEEELKAANKT---------EDGFK---------------K-PILVILSRNGS--REILNESLLVELAEEIGFIVHVLRPDKT-------  359
AT5G55500      HVVFVDGHCTTQLEETWTALFSGIRYAKNFTKPVCFRHAILSPLGYETALFKGLSGEIDCKGDSAHN-------------------------------LWQNPDDKRTARISEFGEMIRAAFGLPVNRHRSLEKPLSSSSSSASVYNVLF  391
Os08g0503800   NVVFVDGHCKAQLEQTWEALFSNVTYVKNFSGPVCFRHAILSPLGYETALFKGLSESFSCEGASAES-------------------------------LREKPDHQKTARLSEFGEMILASFDLLR------DDILSSKTSNG--LNVLF  395
Os01g0118400   AAG--EVHCFP-SAVVSLRAHRELIIERERSLD---GLATPDFTRFLRRALSLPRDAPTRLGDGTG----------------------------------RRPRLLVISRGRT--RLLLNLDAVVRAAEEVGFEVVVNESDVG-------  261
Os01g0118600   D-----VHCFD-HVIVGLVRDRDLILGQHPTRNPK-GYTMVDFTRFLRHAYGLRRDKPMVLGETSG----------------------------------KKPRMLIISRRRT--RKLLNLRQVAAMARELGFEVVVSEAGVGG------  472
Os01g0118700   K-----VRCFP-GAVVGIRMHKEFSIDP--ARDPT-GHSMPEFTKFLRDTFSLPRDAPVSLVDNAAA---------------------------------VRPRLMIISRRHP--RKLMNVEEVVRAAERIGFEVVIGDPP---------  384
Os01g0119000   E-----VRCFR-RVAVGLRMHKEFSVKPELAPGGQ-RLTMADFAAFLRDTYALPR-------AAAAG--------------------------------ARRPRLVVIRRAHY--RKIVNMDEVVRAAEAAGFEAAVMSPR---------  346
Os01g0119100   R-----VHCFR-HVQVGLTCHADFSIDP--SRAPN-GYSMVDFTRFMRATYRLPRDAPFPASGEQQPRRP------------------------------WRPRLLVIARART--RRFVNADEIVRGAERAGFEVVVSEG----------  532
Os01g0498300   R-----THCFP-EAVVGLRIHDELAIDAARMPGN---RTIQDFRRMLDDAYRGRIQMIIEEEEKAAAVALGTPTQGSIRKKSALKD--------------DKPRLVIVSRNGS--RAIENEAELVRAAAGAGFRVAVLQPRQD-------  388
Os01g0956200   R-----THCFP-GAIVGTRFHGILSVDPARLRDN---KTIVDFHDLLAGVYETAGDTVVVDDVTQPAPR--------------------------------RPRLGIVSRRGT--RVIENQAAVARLARSVGFDVDILETANG-------  352
Os02g0135500   ----KDVHCFP-RIVVGATFHKDMGVDPKRSPG---HVSVVDFKRALRRAFGLERVAASRGGATG----------------------------------NGKPRLLIISRKNS--RRFLNEREMAQAAAAVGFEVRIAEPD-Q-------  352
Os02g0327700   Q-----IRCYP-HVIVGLRSHRDLGIDP--SSSPQ-NYTMVDFRLFVREAYGLPAAEVDIPYKADKDDPD------------------------------KKPRIMLIDRGKS--RRFVNVAHVVQGLDWFGFEVVKADPK---------  423
Os02g0329800   G-----VHCFP-QGYLGLYRDRDLIISPHPTRNPR-NYTMVDYNRFLRDALELRRDRPSVLGEEPG----------------------------------MRPRMLIISRAGT--RKLLNLEEVAAAATELGFNVTVAEAG---------  436
Os02g0331200   ----DEVRCYR-SVVVGPTFHKELGVDASRTPS---GYSMVDFRTMLRGALGLERAVAEPSGDRWD----I----------------------------RRRPRLLIISRRNS--RAFLNERAMADMAMSLGFYVRLGEPD-I-------  368
Os04g0196600   DRG--VVRCYP-HVVVGYGSRKEFTIDPSLDDTGG-GYTMVNFTEFLRQSYSLPRDRPIKLGTNHGA----------------------------------RPRMMILERTNS--RKLMNLPEVAAAARAAGFEVTVAGGR---------  418
Os05g0391600   R-----VHCFS-EMIVGLRIHGELVVDPKLMPNG---KGIQDFQALLHQGYSRTPSATAAAAAAQPPVPLALAAPPSRPCLRPDDHAK-----------VAKPKLVIFIRKQN--RVLLNLPHIVTACRRAGFAPHVMNLRRQ-------  429
Os06g0311000   A-----VRCFP-SARVGVESHRVLGIDT--PLTGSNGYTMVGFLAFLRSAYSLPR---HAVTR------TT----------------------------PRRPRVVMVLRRKS--RALTNEAEVVAAVAEAGFEVVAAGPE---------  390
Os06g0470000   E-----VHCFR-RGHLGLYRDRDLIISPHPTRNPR-NYSMVDYNRFLRRAFGLPRDSPAVLGDKTG----------------------------------AKPKMLMIERKGT--RKLLNLRDVAALCEDLGFAVTVAEAG---------  394
Os06g0475400   D-----VRCYP-KITVGLRSHRDLGIDP--ARTQR-NYTMLDFRLYIREVYSLPPAGVDIPFKE--SSMQ------------------------------RRPRAMLINRGRT--RKFVNFQEIAAAVVAAGFEVVPVEPR---------  401
Os06g0707000   R-----VRCYR-RATVGLHSHKDLSIDP--RRAPN-NYSMVDFKRFLMWRYALPREHAIRMEEED---KS------------------------------KKPRLLVINRRSR--RRFVNLDEIVAAAEGVGFEVAAAEL----------  334
Os06g0707200   ----QEVHCFP-RIFIGATFHRAMGIDPARSPG---GVTVADFKRLLRRTFRLERAVASRTGAPR----------------------------------RDKPRLLIISRKSS--RRFLNERAMAHAAALARFDVRIAEPD-N-------  327
Os07g0657400   R-----VHCFP-EVIAGLRIHGELTVDPEKTPEGK---SIRHFRTLLDDAYRGRIQYLERLERRAARSRKRRAAAAKPTTTSIALPIMAPPAKQASPSPPDRPRLVIVSRTGS--RVIENEADVAALAADVGFDVRVVRPERT-------  414
Os10g0492200   ----DEVRCYR-SAVVGPEFHRELGVDPTKTPS---GYSVLDFRKMLRGAFGLDRATATPSGDRWD----I----------------------------RRRPRLLIISRRAARGRAFMNERAMADMAASLGFDVRVGEPD-A-------  362
Os11g0575500   R-----KHCFP-GAIIGTRFHGILSVNPARLRDN---KTIVDFHDLLADVYETAGDTVVVD-VPQPAPR--------------------------------RPRLGIVSCRGK--RVIENQAAVARLARTVGFDVDILETADG-------  258
Os12g0238900   AAGGGEVHCFG-HTVVGLRAHGELIIDRERSPD---GLGMPDFTRFLRRALSLPRDAPTRPGGGHGDATKP----------------------------QPRPRLLIISRRGT--RLLLNTDAVARAAEQVGFEAVASELDMAG------  386
Os02g0330200   ----DQVHCFK-HAIVGLHAYMEFTIDSSKAPH---NYSMVDFNRFMRRTYSLPRDFVTALGEIPK----------------------------------AKPRLLIISRQRT--RMFLNLNEIVAMAEEIGYEVVVEEANVS-------  452
Os03g0567600   ----DQVHCFK-HAIVGLHAYMEFTIDSTKAPH---NYSMADFNRFMRGAYSLGRDSVTVLGEYPK----------------------------------IKPRLLIIKRHRT--RMFLNLDEIISMAEELGFEVVIDEANVS-------  445
               .......610.......620.......630.......640.......650.......660.......670.......680.......690.......700.......710.......720.......730.......740.......750
```

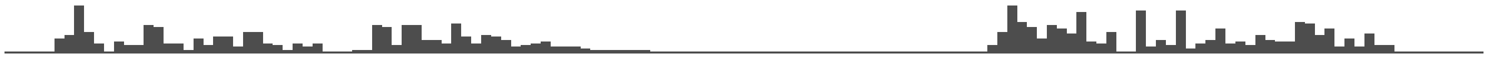

CLUSTAL 2.0.9 MULTIPLE SEQUENCE ALIGNMENT


```
                                     .  :  .
PoGT61_7       ---HNIDSVAHLVNSVDVMVGVHGAGLTNQIFLPKD--------AVVIQIMPIG--LDVLPHFYDFPPKDLKLRYIGYSVSLNESSLFGKYPMDSEVFTNP--RAIQEYEEFK-SIYLDNQDLNIDVSKFRETILKAKELLEP-------   433
PoGT61_4       --TFDVPRFANLVNSCDVLMGIHGAGLTNMLFLPDN--------AVLIQLIPFGELSVIARIDYRDPTPGMNIQYLEYEISANESTLSHQYPIDHPVLRDPGSIHKKGWYAMS-SVYLDNQNVTVDVGKLSATLVQAMKLLVRN-------   452
PoGT61_3       --SANQSRFAQLVNSCDVLMGVHGAGLTNMLFLPDN--------AVSIQMLPFGPLEWWALQQYRDPLPDMNITYLEYRISVEESSLSKQYAPDDPIIADPYSFYAKGWVTIK-RIYLTNITFTIDIERIKGTLVEAMKILQH-------   456
PoGT61_1       --TLTVSAFAQLVNSCDLMMGIHGAALTNMIFLPDN--------AVLLQILPFGEIDNFGRLYFGNPTVGMNIRYLEYKITTEESSLSQQYSTDDPVLSDPMSIHKKGWETIY-SVYLENQNVTVNLGRFKDTLVQAKNLLS--------   492
PoGT61_6       --ILTVVEIAQLVNSCDLLMGIHGAGLTNMVFLPEN--------AVLIQILPFGEIDGSGNTCYRDPTAGMGLRYLEYKITTEESSLSQQYSADDPVLTDPMSIFQKGWSVMA-SVYLENQNITVDLGRFTEVLVQAKKLLQN-------   541
PoGT61_2       ---YHVPSMAKLVNSIDVMVGIHGAGLANMVFLPKD--------AVVIQILPIG--TDLFSSYYRIPAIDMNMKYIEYNVSREESTLAEKYPADSIIFTNPLEMVYRGFIGFR-TIYLENQDVTLNLTKFRDILLKAKGLLQL-------   535
PoGT61_5       --STNQSRFAQLVNSCDVLMGIHGAGLTNMLFLPDN--------AVFIQMLPFGPLDYWTAMEFRDPTWDMSISYLDYRISIEESSLSTQYAPDDPILADPISQYAKGWDFIR-DVYLS-VNFTIDVGRFKGTLVEAMKILQH-------   453
Bradi2g01420   -FDVRISDLAKSVNSFDVLMGVHGAGLTNAAFLPPG--------GVVIQVVPYGKMEGLARTDFGEPVKDMGLEYLEYSVSAQESSLLEMLGPEHLVIKDPEAVHRSGWDKVA-EYYLGKQDVRLDVERFRPTLDKAMEYLRQR------   521
Bradi2g13600   ---TELAQMYRVLNGSDVMVGVHGAAMTHFLFMRPG--------SVFIQVVPLG-TDWAAENYYGEPARRLGLRYMPYKILPAESSLYRRYPRNDPVLTDPVAVNAKGWQVTK-KVYLDGQNVRLDMPRFRRRLREAYAHWAAQRRRQHS   515
Bradi2g01480   --DHEVAPFAELSNTCDVMLGVHGAGLTNMIFLPTG--------GAVIQVVPLGGLE-FVAGYFRGPAADMGLRYLEYRIAPAESSLSEQYPPDHPVFTDPEGVKSKGWDSLK-EAYLDKQDVKLDMRRFRPLLKKAFAHVRANKKLQ--   663
Bradi2g26590   ---TPLPVIHAALASADAMVAVHGAAVTHFLFMRPG--------TVLLQIVPVG-LDWAAESFYGKPAQQLGLEYLEYKVAPEESSLAAEYGLDSTVVRDPSVISSRGWWEMK-KVYMDRQNVTVGIKRFGELLRAAKLHLKNATACANS   545
Bradi2g01380   ---NDISEVGRLINSADVLVGVHGAGLTNMMFLPPG--------ATMVQVVPWGGLQWIARMDYGDPAEAMGLRYVQYEITVDESSLKEKYPRDHEIFTNPTGLHKKGFGFMR-QTLMDGQNITVDVARFKGVLQEALGNYLAQS-----   621
Bradi2g61230   ---APLSASYAAVSACDVLLGVHGADLTKLLFLRPSNNTNSNSTAAVLQIAPLG-VGPIARGCYAEATVSMGLRYEQYDVVAGESSLRLKYAADDVIVADPETAKKGAGWELVAKVYLGSQNVTLDLDRFGDTLRDLHSAILAAAAPGPA   574
Bradi2g25450   ---SDISGFAKLVNSVDVMMGVHGAGLTNCVFLPHN--------ATLIQIVPWGGLDWISRTDFGNPAEMMGLRYKQYAISVDESSLTDQYPKDHEIFKNPISFHKRGFEFIR-RTFMDKQNVKLDCKRFRPILLEALDNLNP-------   574
Bradi4g43180   --STDTSKFARLVNSCDVMVGVHGAGLTNMVFLPAG--------AVLVQVVPYGRLEWLARNTFAEPSAGMEILYLEYVVQLDETTLSEQYPSNHLVLKDPMAIHKQGWDALK-TTYLDKQNVRPHLGRLKNTFLQALKMLPHGRDD---   484
Bradi4g27930   -VFGDMGKFAEVVNSCDVMVGVHGAGLTNMVFLPHN--------GTVVQIIPWGGMKWPCFHALGRPVPDMGLRYVEYEASAEETTLKDVYPRDHAVFTDPLSIHRQGYGMMW-ATFLDGQNVTLDIGRFRGVMEQLYQSVTLA------   448
Bradi4g27360   ---ADVRGFAETVNAADVLLAVHGAGLTNQIFLPTG--------AVMVQIVPWGKMDWMATNFYGQPARDMQLRYVEYYVSEEETTLKDRFPRDHYVFKDPMAIHAQGWPALA-DIVMK-QDVMVNVTRFKPFLLSALDKLQE------   532
Bradi1g19160   ---TEMCKIYRELNGSDAMVGVHGAAMTHFLFMRPG--------KVFIQVVPLG-TDWAAGAYYGEPAARLGLRYVGYKIRPDESSLAREYPAGDPVLVDPAAVAKRGWDVTK-KVYLDRQNVRLDLARFREELVKAHRYLVSSGAGAA-   536
Bradi1g06560   --EADVPAFAAMVNSADVLLAVHGAGLTNQIFLPTN--------AVVLQIVPWGNMDWMATNFYGQPAREMQLRYVEYYVGEEETSLKDKYPRDHVVFRDPKALHTQGWETLA-DTIMK-QDVQVDLSRFRPFLLQAIDKLQE------   565
Bradi1g34670   --HTDMPNFARLVNSADIMMGVHGAGLTNMVFLPSR--------AVLLQVVPFGGLEWLSRVTFKDPAKDMDVNYMEYNVSLEESSLRNLYPEGHFYLKHPYDVHKKGWDAIK-TVYLDKQSVRLNLTKFVQTLELARSRLPA-------   455
Bradi3g11300   --STDVGKFARLVNSADVMVGVHGAGLTNMLFLPAG--------AVLVQIVPYGGLEWLARGTFKEPSPDMRIHYLEYMIQLDETTLSEQYAKDDPVLRDPASIHKQGWDKLK-TVYLDKQNVRPHLGRLKITFMEALKLLPHGRGQSN-   501
Bradi3g03120   --HTDMATFARLVNSADVMVGVHGAGLTNMVFLPAG--------AVLVQVVPFGGLEWLTRVTFKEPAADMEVRYMDYNVQLEESSLLDQYPRSHQVLADPYAVHKQGWDALK-TAYLDKQNVRLDLDRFRATLRDALALLPPAAAPLPA   501
Bradi3g11310   ---SDLSHFSKAVNSVDVMMGVHGAGLTNCVFLPHN--------ATLIQIVPWGGLEGVCRIDFGDPSEQMGLRYKQYSISVHESSLTDQYPLDHEIFKNPLAFHK-GFEFIR-QTFMDKQNVRLDCNRFRPILLETLDQLNQ-------   575
AT2G03360      ---TSLREAYKLIKSSHGMIGVHGAALTQLLFLRPG--------SVLVQIVPVG-LGWVSKTCFETPAKAMKLDYTEYRVNVEESSLIEKYSRDDLVLKDPIAYRGMDWNVTKMKVYLKDQDVRLDVNRFRKHMNEAYKKAKSFMDLNG-   455
AT2G03370      ---TNLKEAYKLIKSSHGMVGVHGAALTHLLFLRPG--------SIFVQIVPLG-LGWASKPCYESPAKTMKLEYLEYKVNVEESSLIEKYNRDDLVLKDPIAYRGMDWNATKMKVYLKEQDVSLDVNRFRKHMNEAYKKAKIFMDING-   452
AT2G41640      ---TEMAKIYRSLNTSDVMIGVHGAAMTHFLFLKPK--------TVFIQIIPLG-TDWAAETYYGEPAKKLGLKYVGYKIAPKESSLYEEYGKDDPVIRDPDSLNDKGWEYTK-KIYLQGQNVKLDLRRFRETLTRSYDFSIRRRFREDY   494
AT3G10320      ---TELAKIYRVLNSSKVMVGVHGAAMTHFLFMKPG--------SIFIQIIPLG-TDWAAETYYGEPAKKLGLDYNGYKILPRESSLYEKYDKDDPILKDPNSITKKGWQFTK-GIYLNDQKVRLDLHRFKKLLIDAYAKSIR-------   494
AT3G18170      --NIGIAKFAQTVNSCDVMLGVHGAGLTNMVFLPEN--------AVVIQVLPIGGFEWLAKTDFEKPSEGMNLRYLEYKIAVEESTLVKKYGRDHEIVRDPSAVAKHGWEMFK-SVYLVQQNVSIDINRFKPVLVKALELL---------   384
AT3G18180      --NTEIASFAITVNSCDVMLGVHGAGMTNMVFLPDN--------AIVIQILPIGGFEWLAKMDFEYPSKGMNLRYLEYKITAEESTLVKQYGRDHEFVRDPLAVAKRGWGTFK-SVYLVQQNVSVDINRFKLVLVKALELLHNQSV----   470
AT3G57380      ---TELAKIYRCLNSSDVMIGVHGAAMTHLLFLKPK--------TVFIQIIPIG-TEWAAETYYGKPAKKMRLKYIGYKIKPKESSLYDEYGIDDPIIRDPKSFTQKGWDYTK-KIYLERQNVKLDLKRFRKPLSRAYDFSMKR-IGLVY   495
AT5G55500      VRREDYLAHPRHGGKVQSRLINEEEVFDSLHHWVATGSTGL---TKCGINLVNGLLAHMSMKDQVRAIQDASVIIGAHGAGLTHIVSATPNTTIFEIISVEFQRPHFELIAKWKGLEYHAMHLANSRAEPTAVIEKLTEIMKSLGC----   534
Os08g0503800   VRREDYLAHPRHSGKVESRLSNEKEVYDAIEGWAKG--------QKCKINVINGLFAHMNMKEQLRAIQEASVVIGAHGAGLTHLVSATPDTKVLEIISSMYRRPHFALISHWKSLEYHAINLPGSYARVTDVINELSNILKGFGC----   533
Os01g0118400   ---NSIEQVGKLINSCDALLGVHGAGLTNMMFLPPG--------ATMVQVVPWGGLQWIARMDYGEPAAAMGLNYIQYEIAVAESSLKDKYPAGDEIFTNPTGLHKRGFAFMK-QTLMDGQDITIDVTRFRPVLQQALDNLATK------   393
Os01g0118600   -GSGGVKRFASAVNSCDVLVGVHGAGLTNQAFLPRG--------GVVVQIVPWGRMEWMATNFYGAPAAAMELRYVEYHVAAEESSLARRYPREHAVFRDPMAIHGQGWKALA-DIVMT-QDVKLNLRRFRPTLLRVLDLLQD-------   604
Os01g0118700   -FNVDVGEFAKEVNRADVLMGVHGAGLTNSVFLPTG--------AVLIQVVPYGKMEHIGKVDFGDPAEDMRLKYMAYSAGVEESTLVETLGRDHPAVRDPESVHRSGWGKVA-EYYLGKQDIRLDLARFEPLLRDAMDYLKHQ------   518
Os01g0119000   -FDEPVEEVARKVNAFDAMVGVHGAGLTNAVFLPAG--------AVVIQVVPYGRLERMARADFGEPVADMGLRYMEYSVAADESTLLEMLGPEHQVVKDPEAVHRSGWDKVA-EYYLGKQDVRINVARFAATLAAAFDHLRPSHS----   482
Os01g0119100   --EHEVAPFAELANTCDAMVGVHGAGLTNMVFLPTG--------GVVIQVVPLGGLE-FVAGYFRGPSRDMGLRYLEYRITPEESTLIDQYPRDHPIFTDPDGVKSKGWNSLK-EAYLDKQDVRLDMKRFRPILKKAIAHLRKNSGNNNT   670
Os01g0498300   ---TELAKMYRALNASDVMVGVHGAAMTHFLFMRPG--------SVFIQVVPLG-TDWAAETYYGEPARRLGLRYMPYKIKPAESSLYRQYAKDDAVLTDPDTVNAKGWQVTK-KVYLDGQNVRLDMVRFRRRLRDAYDHWAELRRRHNA   525
Os01g0956200   ---LPLPASYASVSACDVLVGVHGADLTKLLFLRPG--------AALVQIAPLG-VAPIARGCYAEASARMGLHYEQYDAEGHESSLSRKYGLRDVVVSDPEAAKRDKGWGFVARVYLGGQNVTLDLSRFRHTLTRLHARALRVRSLHPA   490
Os02g0135500   --HTDMSTFAQLVNSADVMIGVHGAGLTNMVFLPRG--------AVLIQVVPFGGLEWLTTVTFKNPAKDMEVTYMDYNVQLEESSLIDQYPRNHQVLTDPYAVHKQGWDALK-TAYLDKQNIKMDMDRFKKTLQEALDRLPPA------   485
Os02g0327700   -IDSNLDEFVRLVDSCDAIMGVHGAGLTNMVFLRSG--------GVVVHIVPYG-IKFMADGFYGAPARDMGLRHVEYSISPEESTLLEKYGWNHTVINDPETIRKGGWEKVA-EFYMSKQDIVLNMTRFGPSLLNAIEFIM--------   554
Os02g0329800   ---ADVPAFAALVNSADVLLAVHGAGLTNQIFLPAE--------AVVVQIVPWGNMDWMATNFYGQPARDMQLRYVEYYVGEEETSLKHNYSRDHMVFKDPKALHAQGWQTLA-ATIMK-QDVEVNLTRFRPILLQALDRLQQ-------   566
Os02g0331200   --STDVSKFARLVNSADVMVGVHGAGLTNMVFLPAG--------AVLIQVVPYGGLEWLARGTFKEPAADMQIHYLEYMIQLDETTLSEQYPKDDPVLKDPYSIHKQGWNALK-MVYLDKQNVRPHLGRLKNTFMEALKLLPHG--HTN-   504
Os04g0196600   -PTSTYDEFAREVNSYDVMVGVHGAGLTNCVFLPTG--------AVLLQIVPYGRLESIAQTDFGEPARDMGLRYIEYDIAADESSLMDVFGKDHPMIKDPVAVHLSGWGNVA-EWYLGKQDVRVNIERFRPFLTQALEHLQ--------   550
Os05g0391600   ---TPLPAIHAALSSADAMVAVHGAAVTHFLFMRPG--------SVLLQIVPVG-LDWAADAFYGKPAQQLGLGYLEYKVAPEESSLAAEYGVNSTVVRDPSVISSRGWWEMK-KVYMDRQNVTVNIKRFGELLRSARLHLKNATACGKA   566
Os06g0311000   -EAGDVAGFAATVNSCDVMVGVHGAGLTNMVFLPRN--------GTVVQIIPWGGMKWPCWYDYGEPVPAMGLRYVEYEVAANETTLRERYPMDHPVFADPVSIHRKGFNHLW-STFLNGQNLTLDVNRFKAVMAEVYTSITAAPV----   526
Os06g0470000   ---ADVRGFAEKVNAADVLLAVHGAGLTNQIFLPTG--------AVLVQIVPWGKMDWMATNFYGQPARDMRLRYVEYYVSEEETTLKDKYPRDHYVFKDPMAIHAQGWPALA-EIVMK-QDVTVNVTRFKPFLLKALDELQE------   524
Os06g0475400   -RDLSIEEFSRVVDSCDVLMGAHGAGLTNFFFLRTN--------AVMLQVVPWGHMEHPSMVFYGGPAREMRLRDVEYSIAAEESTLYDKYGKDHPAIRDPESIHKQGWQFGM-KYYWIEQDIKLNVTRFAPTLQQVLQMLRG-------   534
Os06g0707000   --DAHIPAAASAVNSYDAMVAVHGSGLTNLVFLPMN--------AVVIQVVPLGRMEGLAMDEYGVPPRDMNMRYLQYNITAEESTLSEVYPRAHPVFLDPLPIHKQSWSLVK-DIYLGQQDVRLDVRRFRPVLLKALHLLR--------   465
Os06g0707200   --HTDMPNFARLVNSADVMMGVHGAGLTNMVFLPSR--------AVLIQVVPFGGLEWLTRVTFKDPAKDMDVNYMEYNVSFDESSLRELYPRDHFYIQHPYDVHKKGWDAIK-TVYLDKQNVELNLTKLTNTLERARDFLPEP------   460
Os07g0657400   ---TELCKIYRELNASDAMVGVHGAAMTHFLFMRPG--------KVFVQVVPLG-TDWAAGAYYGEPAARLGLRYVGYKILPEESSLSREYPTGDPVLTDPAGVGKRGWDVTK-KVYLDRQNVRLDLPRFREVLVGAHRHLVAGKRRRRQ   551
Os10g0492200   --STDTSKFARLVNSCDVMVGVHGAGLTNMVFLPAG--------AVLVQVVPYGKLEWLARNTFAEPSSAMEIHYLEYAVQLDETTLSEQYPADHPVLRDPMAIHKQGWEALK-TTYLDKQNVRPHLGRLKNTFLQALKLLPHDKETMN-   500
Os11g0575500   ---LQLPASYASVSACDVLVGVHSADLTKLLFLRPG--------AALV------------------------------------------------------------------------------------------------   295
Os12g0238900   ADHDDVARVARLVNSFDAVVGVHGAGLTNMVFLPPG--------AAAVQIVPWGGLRWLARADFGEPAVAMGLRYIQYEVAAGESTLKDKYPRDHEIFTNPTALHKKGFTFMR-HTFLNGQDIIVDIDRFKPVLLRALNSLAR-------   520
Os02g0330200   ---SDLSHFGKVVNSVDVMMGVHGAGLTNCVFLPQN--------ATLIQIVPWGGLDWISRIDFGNPAEQMGLRYKQYSIGVHESSLTDQYPLDHEIFTNPLSFHKHGFEFIR-QTFMDKQNVKLDCNRFKPVLLEVLDQLNQ-------   583
Os03g0567600   ---SDISRFARLVNSVDVMMGVHGAGLTNCVFLPQH--------ATLIQIVPWGGLDWISRTDFGNPAELMGLRYKQYSIGVDESSLTDQYPRDHEIFKNPISFHQRGFDFIR-QTFMDKQNVKLDCKRFRPILLEALDNLNP-------   576
               .......760.......770.......780.......790.......800.......810.......820.......830.......840.......850.......860.......870.......880.......890.......900
```

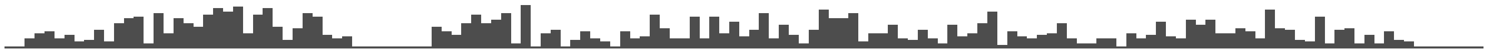

CLUSTAL 2.0.9 MULTIPLE SEQUENCE ALIGNMENT


```
PoGT61_7      ---------------   433
PoGT61_4      ---------------   452
PoGT61_3      ---------------   456
PoGT61_1      ---------------   492
PoGT61_6      ---------------   541
PoGT61_2      ---------------   535
PoGT61_5      ---------------   453
Bradi2g01420  --------------    521
Bradi2g13600  KPL-----------    518
Bradi2g01480  --------------    663
Bradi2g26590  NGNGKAAAVR----    555
Bradi2g01380  --------------    621
Bradi2g61230  ARP-----------    577
Bradi2g25450  --------------    574
Bradi4g43180  --------------    484
Bradi4g27930  --------------    448
Bradi4g27360  --------------    532
Bradi1g19160  --------------    536
Bradi1g06560  --------------    565
Bradi1g34670  --------------    455
Bradi3g11300  --------------    501
Bradi3g03120  --------------    501
Bradi3g11310  --------------    575
AT2G03360     ---------------   455
AT2G03370     ---------------   452
AT2G41640     LLHRED---------   500
AT3G10320     ---------------   494
AT3G18170     ---------------   384
AT3G18180     ---------------   470
AT3G57380     ISHKPDVTN------   504
AT5G55500     ---------------   534
Os08g0503800  ---------------   533
Os01g0118400  ---------------   393
Os01g0118600  ---------------   604
Os01g0118700  ---------------   518
Os01g0119000  ---------------   482
Os01g0119100  THN------------   673
Os01g0498300  DSTETEQRKPW----   536
Os01g0956200  P--------------   491
Os02g0135500  ---------------   485
Os02g0327700  ---------------   554
Os02g0329800  ---------------   566
Os02g0331200  ---------------   504
Os04g0196600  ---------------   550
Os05g0391600  ASAATTTAASATSWS   581
Os06g0311000  ---------------   526
Os06g0470000  ---------------   524
Os06g0475400  ---------------   534
Os06g0707000  ---------------   465
Os06g0707200  ---------------   460
Os07g0657400  RESQ-----------   555
Os10g0492200  ---------------   500
Os11g0575500  ---------------   295
Os12g0238900  ---------------   520
Os02g0330200  ---------------   583
Os03g0567600  ---------------   576
              .......910.....
```
