# Supplementary material for: Discovery of diversity in xylan biosynthetic genes by transcriptional profiling of a heteroxylan containing mucilaginous tissue
Source: Front Plant Sci. 2013 Jun 7;4:183. doi: 10.3389/fpls.2013.00183 (PMC3675317; doi:10.3389/fpls.2013.00183)
Supplement: Table S1 — 454 FLX sequencing and assembly of sequence reads. [file DataSheet5.PDF]

Supplemental Table S1. 454 FLX sequencing and assembly of sequence reads

Developmental stage	Mucilaginous layer				
	6 DPA[a]	8 DPA	10 DPA	12 DPA	Stem
Total sequence reads	$1.62x10^5$	$1.22x10^5$	$1.02x10^5$	$2.04x10^5$	$5.09x10^5$
Total sequence reads, all stages			$1.10x10^6$		
Total assembled contigs			33,167		
Total assembled contigs, reads ≥ 5			15,841		

[a] Days post anthesis.

Supplemental Table S3. Glycosyl linkages identified in psyllium mucilaginous heteroxylan and putative glycosyltransferases identified in this study that are hypothesized to form these linkages

Glycosyl linkage	Candidate glycosyltransferase(s)
Backbone (1→4)-linked Xyl*p*[1,2,3]	PoIRX10_1 to _4
Singel Xyl*p* side chain on O-2 of backbone Xyl*p*[1]	PoGT61_1 to _7 and/or PoIRX10_1 to _4
α-Ara*f*-(1→3)-β-Xyl*p*-(1→3)-Ara*f* side chain on O-3 of backbone Xyl*p*[1]	PoGT61_1 to _7 and/or PoIRX10_1 to _4
Rhamnose, glucose, glucoronic acid, galactose, and mannose in side chain[1,2,3]	Psyllium homologs of AT4G32290 and AT2G32750

[1]Fischer et al., 2004; [2]Guo et al., 2008; [3]Yin et al., 2012
