# Supplementary material for: Discovery of diversity in xylan biosynthetic genes by transcriptional profiling of a heteroxylan containing mucilaginous tissue
Source: Front Plant Sci. 2013 Jun 7;4:183. doi: 10.3389/fpls.2013.00183 (PMC3675317; doi:10.3389/fpls.2013.00183)
Supplement: File S2 — Protein sequence alignment of IRX10 proteins. [file DataSheet2.PDF]

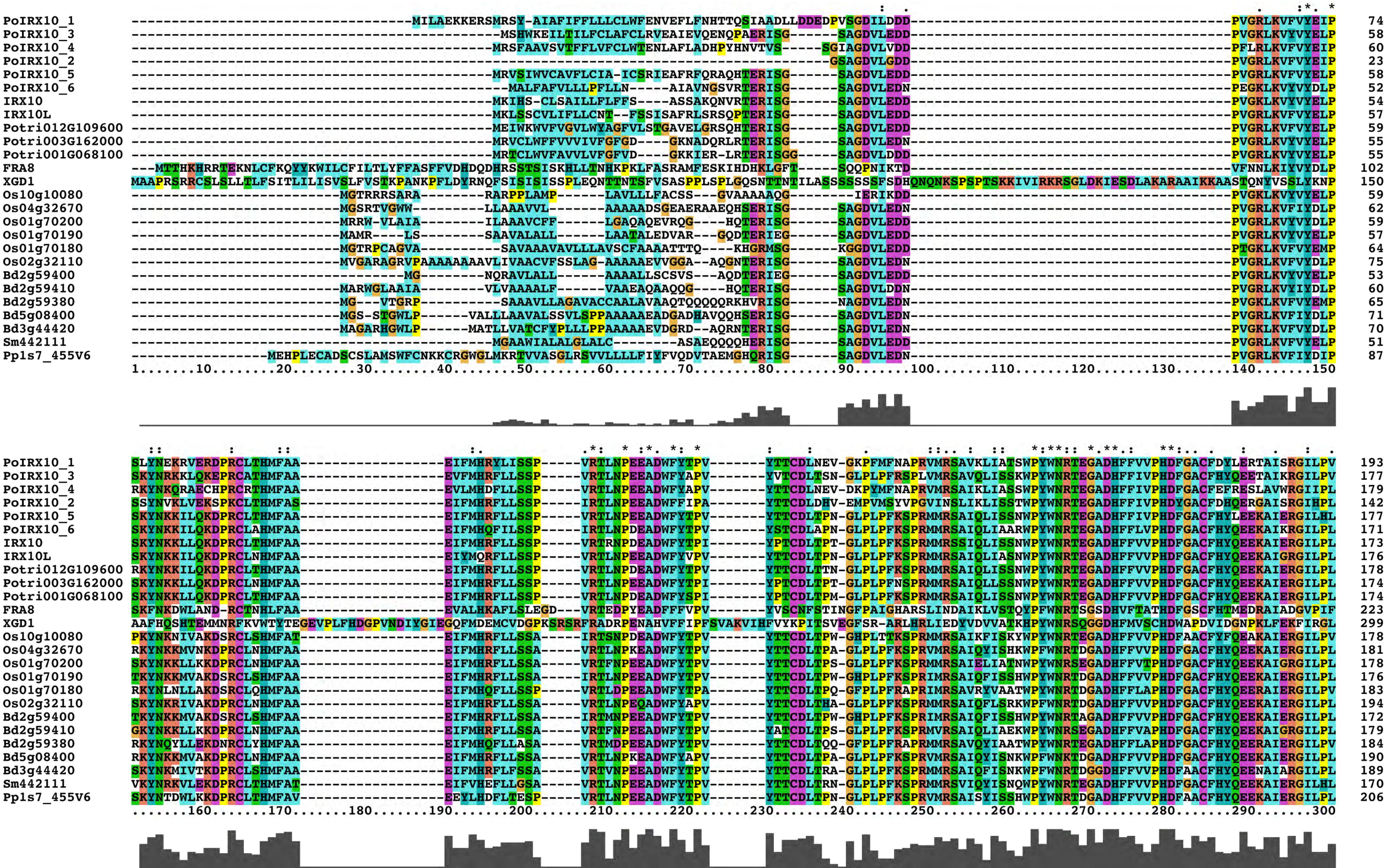

PoIRX10_1 -----------------------------------MILAEKKERSMRSY-AIAFIFFLLLCLWFENVEFLFNHTTQSIAADLLDDEDPVSGDILDDD--PVGRLKVFVYEIP 74
PoIRX10_3 --MSHWKEILTILFCLAFCLRVEAIEVQENQPAERISG------SAGDVLEDD--PVGKLKVYVYELP 58
PoIRX10_4 ---MRSFAAVSVTFFLVFCLWTENLAFLADHPYHNVTVS-----SGIAGDVLVDD--PFLRLKVFVYEIP 60
PoIRX10_2 ---GSAGDVLGDD--PVGRLKVFVYEIP 23
PoIRX10_5 ---MRVSIWVCAVFLCIA-ICSRIEAFRFQRAQHTERISG------SAGDVLEDD--PVGRLKVFVYELP 58
PoIRX10_6 ---MALFAFVLLLPFLLN-----AIAVNGSVRTERISG------SAGDVLEDN--PEGKLKVYVYDLP 52
IRX10 ---MKIHS-CLSAILLFLFFS----ASSAKQNVRTERISG------SAGDVLEDD--PVGKLKVYVYELP 54
IRX10L ---MKLSSCVLIFLLCNT--FSSISAFRLSRSQPTERISG------SAGDVLEDD--PVGRLKVFVYELP 57
Potri012G109600 ---MEIWKWVFVGVLWYAGFVLSTGAVELGRSQHTERISG------SAGDVLEDD--PVGRLKVFVYELP 59
Potri003G162000 ---MRVCLWFFVVVIVFGFGD----GKNADQRLRTERISG------SAGDVLEDN--PVGRLKVFVYELP 55
Potri001G068100 ---MRTCLWVFAVVLVFGFVD----GKKIER-LRTERISGG-----SAGDVLDDD--PVGRLKVYVYELP 55
FRA8 ---MTTHKHRRTEKNLCFKQYYKWILCFILTLYFFASFFVDHDQDHRSSTSISKHLLTNHKPKLFASRAMFESKIHDHKLGFT-----SQQPNIKTD--VFNNLKIYVYDLP 102
XGD1 MAAPRSRRCSLSLLTLFSITLILISVSLFVSTKPANKPFLDYRNQFSISISISSPLEQNTTNTSFVSASPPLSPLGQSNTTNTILASSSSSSSFSDHQNQNKSPSPTSKKIVIRKRSGLDKIESDLAKARAAIKKAASTQNVVSSLYKNP 150
Os10g10080 --------------------------MGTRRRSARA--------RARPPLAMP------LAVLLLFACSS---GVAAAAAQG--------IERIKDD--PVGRLKVYVYELP 59
Os04g32670 --------------------------MGSRTVGWW---------LLAAAVVL-------AAAAADSGEAERAAEQHSERISG------SAGDVLEDN--PVGRLKVFIYDLP 62
Os01g70200 --------------------------MRRW-VLAIA--------ILAAAVCFF------LGAQAQEVRQG----HQTERISG------SAGDVLEDD--PVGRLKVYVYDLP 59
Os01g70190 --------------------------MAMR----LS--------SAAVALALL------LAATALEDVAR---GQDTERIEG------SAGDVLEDD--PVGRLKVYVYELP 57
Os01g70180 --------------------------MGTRPCAGVA----------SAVAAAVAVLLLAVSCFAAAATTTQ----KHGRMSG------KGGDVLEDD--PTGKLKVFVYEMP 64
Os02g32110 --------------------------MVGARAGRVPAAAAAAAAVLIVAACVFSSLAG-AAAAAEVVGGA--AQGNTERISG------SAGDVLEDN--PVGRLKVFVYDLP 75
Bd2g59400 ----------------------------------MG--------NQRAVLALL------AAAALLSCSVS---AQDTERIEG------SAGDVLEDD--PVGRLKVYVYELP 53
Bd2g59410 --------------------------MARWGLAAIA--------VLVAAAALF------VAAEAQAAQQG----HQTERISG------SAGDVLDDN--PVGRLKVYIYDLP 60
Bd2g59380 --------------------------MG---VTGRP----------SAAAVLLAGAVACCAALAVAAQTQQQQQRKHVRISG------NAGDVLEDN--PVGKLKVFVYEMP 65
Bd5g08400 --------------------------MGS-STGWLP------VALLLAAVALSSVLSPPAAAAAEADGADHAVQQHSERISG------SAGDVLEDN--PVGRLKVFIYDLP 71
Bd3g44420 --------------------------MAGARHGWLP------MATLLVATCFYPLLLPPAAAAAEVDGRD--AQRNTERISG------SAGDVLEDN--PVGKLKVFVYDLP 70
Sm442111 ---MGAAWIALALGLALC--------ASAEQQQQHERISG------SAGDVLEDD--PVGRLKVFVYELP 51
Pp1s7_455V6 -----------------MEHPLECADSCSLAMSWFCNKKCRGWGLMKRTVVASGLRSVVLLLLFIYFVQDVTAEMGHQRISG------SAGDVLEDN--PVGRLKVFIYDIP 87
1.......10........20........30........40........50........60........70........80........90.......100.......110.......120.......130.......140.......150

PoIRX10_1 SLYNEKRVERDPRCLTHMFAA------------------EIFMHRYLISSP-----VRTLNPEEADWFYTPV-------YTTCDLNEV-GKPFMFNAPRVMRSAVKLIATSWPYWNRTEGADHFFVVPHDFGACFDYLERTAISRGILPV 193
PoIRX10_3 SKYNRKKLQKEPRCATHMFAA------------------EVFMHRFLLSSP-----VRTLNPEEADWFYAPV-------YVTCDLTSN-GLPLPFRSPLVMRSAVQLISSKWPYWNRTEGADHFFIVPHDFGACFHYQEETAIKRGILPV 177
PoIRX10_4 RKYNKQRAECHPRCRTHMFAA------------------EVLMHDFLLSSP-----VRTLNPEEADWFYAPV-------YTTCDLNEV-DKPYMFNAPRVMRSAIKLIASSWPYWNRTEGADHFFIVPHDFGACFEFRESLAVWRGIIPL 179
PoIRX10_2 SSYNVKLVEKSPKCLTHMFAS------------------EIFMHRFLLSSP-----IRTLNPEEADWFFIPA-------YTTCDLDHV-EMPVMSYVPGVINSLIKLISSTWPYWNRTEGADHFFIVPHDYGACFDHQERGAISRGIHPL 142
PoIRX10_5 SKYNKKILQKDPRCLTHMFAA------------------EIFMHRFLLSSP-----VRTLNPEEADWFYTPV-------YTTCDLTPN-GLPLPFKSPRMMRSAIQLISSNWPYWNRTEGADHFFIVPHDFGACFHYLEEKAIERGILHL 177
PoIRX10_6 SKYNKKILQKDPRCLAHMFAA------------------EIFMHQFILSSP-----IRTLNPDEADWFYTPV-------YSTCDLAPN-GLPLPFKSPRMMRSAIQLIAARWPYWNRTEGADHFFLVPHDFGACFHYQEEKAIKRGILPL 171
IRX10 SKYNKKLLQKDPRCLTHMFAA------------------EIFMHRFLLSSP-----VRTRNPDEADWFYTPI-------YPTCDLTPT-GLPLPFKSPRMMRSSIQLISSNWPYWNRTEGADHFFVVPHDFGACFHYQEEKAIERGILPL 173
IRX10L SKYNKKILQKDPRCLNHMFAA------------------EIYMQRFLLSSP-----VRTLNPEEADWFYVPV-------YTTCDLTPN-GLPLPFKSPRMMRSAIQLIASNWPYWNRTEGADHFFVVPHDFGACFHYQEEKAIGRGILPL 176
Potri012G109600 RKYNKKILQKDPRCLTHMFAA------------------EIFMHRFLLSSP-----VRTLNPDEADWFYTPV-------YTTCDLTTN-GLPLPFKSPRMMRSAIQLISSNWPYWNRTEGADHFFVVPHDFGACFHYQEEKAIERGILPL 178
Potri003G162000 SKYNKKLLQKDPRCLNHMFAA------------------EIFMHRFLLSSP-----VRTLNPEEADWFYTPI-------YPTCDLTPT-GLPLPFNSPRMMRSAIQLLSSNWPYWNRTEGADHFFVVPHDFGACFHYQEEKAIERGILPL 174
Potri001G068100 SKYNKKLLQKDPRCLTHMFAA------------------EIFMHRFLLSSP-----VRTLNPDEADWFYSPI-------YPTCDLTPM-GLPLPFKSPRMMRSAIQLISSNWPYWNRTEGADHFFVVPHDFGACFHYQEEKAIERGILPL 174
FRA8 SKFNKDWLAND-RCTNHLFAA------------------EVALHKAFLSLEGD---VRTEDPYEADFFFVPV-------YVSCNFSTINGFPAIGHARSLINDAIKLVSTQYPFWNRTSGSDHVFTATHDFGSCFHTMEDRAIADGVPIF 223
XGD1 AAFHQSHTEMMNRFKVWTYTEGEVPLFHDGPVNDIYGIEGQFMDEMCVDGPKSRSRFRADRPENAHVFFIPFSVAKVIHFVYKPITSVEGFSR-ARLHRLIEDYVDVVATKHPYWNRSQGGDHFMVSCHDWAPDVIDGNPKLFEKFIRGL 299
Os10g10080 PKYNKNIVAKDSRCLSHMFAT------------------EIFMHRFLLSSA-----IRTSNPDEADWFYTPV-------YTTCDLTPW-GHPLTTKSPRMMRSAIKFISKYWPYWNRTEGADHFFVVPHDFAACFYFQEAKAIERGILPV 178
Os04g32670 RKYNKKMVNKDPRCLNHMFAA------------------EIFMHRFLLSSA-----VRTLNPKEADWFYTPV-------YTTCDLTPA-GLPLPFKSPRVMRSAIQYISHKWPFWNRTDGADHFFVVPHDFGACFHYQEEKAIERGILPL 181
Os01g70200 SKYNKKLLKKDPRCLNHMFAA------------------EIFMHRFLLSSA-----VRTFNPEEADWFYTPV-------YTTCDLTPS-GLPLPFKSPRMMRSAIELIATNWPYWNRSEGADHFFVTPHDFGACFHYQEEKAIGRGILPL 178
Os01g70190 TKYNKKMVAKDSRCLSHMFAA------------------EIFMHRFLLSSA-----IRTLNPEEADWFYTPV-------YTTCDLTPW-GHPLPFKSPRIMRSAIQFISSHWPYWNRTDGADHFFVVPHDFGACFHYQEEKAIERGILPL 176
Os01g70180 RKYNLNLLAKDSRCLQHMFAA------------------EIFMHQFLLSSP-----VRTLDPEEADWFYTPA-------YTTCDLTPQ-GFPLPFRAPRIMRSAVRYVAATWPYWNRTDGADHFFLAPHDFGACFHYQEERAIERGILPV 183
Os02g32110 SKYNKRIVAKDPRCLNHMFAA------------------EIFMHRFLLSSA-----VRTLNPEQADWFYAPV-------YTTCDLTHA-GLPLPFKSPRMMRSAIQFLSRKWPFWNRTDGADHFFVVPHDFGACFHYQEEKAIERGILPL 194
Bd2g59400 TKYNKKMVAKDSRCLSHMFAA------------------EIFMHRFLLSSA-----IRTMNPEEADWFYTPV-------YTTCDLTPW-GHPLPFKSPRIMRSAIQFISSHWPYWNRTAGADHFFVVPHDFGACFHYQEEKAIERGILPL 172
Bd2g59410 GKYNKKLLKKDPRCLNHMFAA------------------EIFMHRFLLSSA-----VRTFNPEEADWFYTPV-------YATCDLTPS-GLPLPFKSPRMVRSAIQLIAEKWPYWNRSEGADHFFVAPHDFGACFHYQEEKAIGRGILPL 179
Bd2g59380 RKYNQYLLEKDNRCLYHMFAA------------------EIFMHQFLLASA-----VRTMDPEEADWFYTPV-------YTTCDLTQQ-GFPLPFRAPRMMRSAVQYIAATWPYWNRTEGADHFFLAPHDFGACFHYQEERAIERGILPV 184
Bd5g08400 RKYNKKMVAKDPRCLNHMFAA------------------EIFMHRFLLSSA-----VRTLNPKEADWFYAPV-------YTTCDLTPA-GLPLPFKSPRVMRSAIQYISNKWPFWNKTDGADHFFVVPHDFGACFHYQEEKAIERGILPL 190
Bd3g44420 SKYNKMIVTKDPRCLSHMFAA------------------EIFMHRFLFSSA-----VRTVNPEEADWFYTPV-------YTTCDLTRA-GLPLPFKSPRMMRSAIQFISNKWPFWNRTDGGDHFFVVPHDFAACFHYQEENAIARGILPL 189
Sm442111 VKYNRKVLEKDPRCLTHMFAT------------------EIFVHEFLLGSA-----VRTLNPEEADWFYTPV-------YTTCDLTRN-GLPLPFKSPRMMRSVIQYISNQWPYWNRTEGADHFFVVPHDFGACFHYQEEKAIERGILHL 170
Pp1s7_455V6 SKYNTDWLKKDPRCLTHMFAV------------------EEYLHDFLTESP-----VRTLNPEEADWFYTPV-------YTTCDLTPN-GLPLPFKSPRVMRSAISYISSHWPYWNRTDGADHFFVVPHDFAACFHYQEEKAIERGILPL 206
.......160.......170.......180.......190.......200.......210.......220.......230.......240.......250.......260.......270.......280.......290.......300

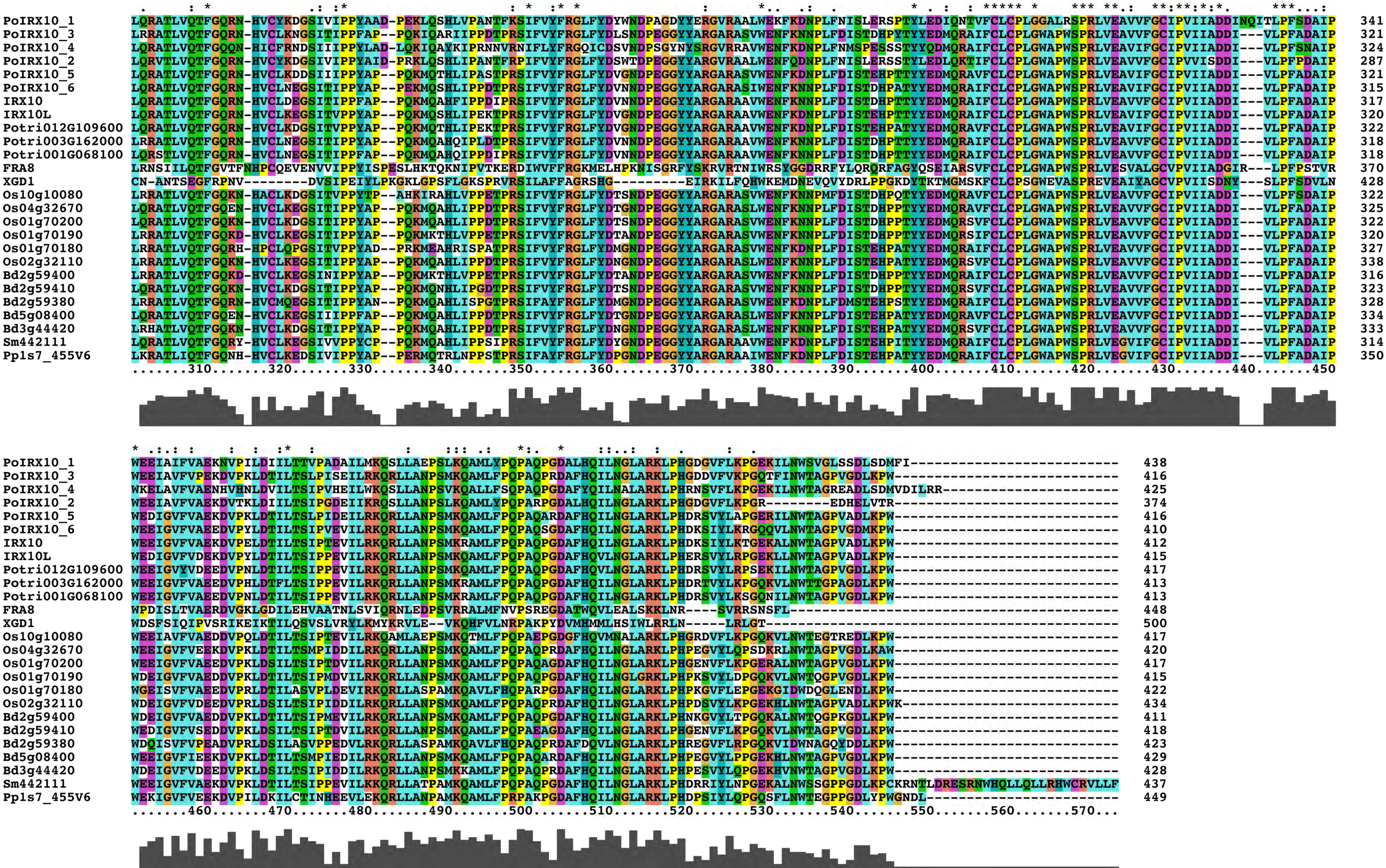

PoIRX10_1 LQRATLVQTFGQRN-HVCYKDGSIVIPPYAAD-PEKLQSHLVPANTFKSIFVYFRGLFYDYWNDPAGDYYERGVRAALWEKFKDNPLFNISLERSPTYLEDIQNTVFCLCPLGGALRSPRLVEAVVFGCIPVIIADDINQITLPFSDAIP 341
PoIRX10_3 LRRATLVQTFGQRN-HVCLKNGSITIPPFAP--PQKIQARIIPPDTPRSIFVYFRGLFYDLSNDPEGGYYARGARASVWENFKNNPLFDISTDHPYTYYEDMQRAIFCLCPLGWAPWSPRLVEAVVFGCIPVIIADDI---VLPFADAIP 321
PoIRX10_4 LQRATLVQTFGQQN-HICFRNDSIIIPPYLAD-LQKIQAYKIPRNNVRNIFLYFRGQICDSVNDPSGYNYSRGVRRAVWENFKDNPLFNMSPESSSTYYQDMQRAIFCLCPLGWAPWSPRLVEAVIFGCIPVIIADDI---VLPFSNAIP 324
PoIRX10_2 LQRVTLVQTFGQRN-HVCYKDGSIVIPPYAID-PRKLQSHLIPANTFRPIFVYFRGLFYDSWTDPEGGYYARGVRAALWENFQDNPLFNISLERSSTYLEDLQKTIFCLCPLGWAPWSPRLVEAVVFGCIPVVISDDI---VLPFPDAIP 287
PoIRX10_5 LQRATLVQTFGQRN-HVCLKDDSIIIPPYAP--PQKMQTHLIPASTPRSIFVYFRGLFYDVGNDPEGGYYARGARASVWENFKDNPLFDISTDHPATYYEDMQRAVFCLCPLGWAPWSPRLVEAVIFGCIPVIIADDI---VLPFADAIP 321
PoIRX10_6 LQRATLVQTFGQRN-HVCLNEGSITIPPYAP--PEKMQSHLIPPDTPRSIFVYFRGLFYDVNNDPEGGYYARGARASIWENFKNNPLFDISTDHPATYYEDMQRAIFCLCPLGWAPWSPRLVEAVIFGCIPVIIADDI---VLPFADAIP 315
IRX10 LQRATLVQTFGQRN-HVCLDEGSITIPPFAP--PQKMQAHFIPPDIPRSIFVYFRGLFYDVNNDPEGGYYARGARAAVWENFKNNPLFDISTDHPTTYYEDMQRAIFCLCPLGWAPWSPRLVEAVVFGCIPVIIADDI---VLPFADAIP 317
IRX10L LQRATLVQTFGQRN-HVCLKEGSITVPPYAP--PQKMQSHLIPEKTPRSIFVYFRGLFYDVGNDPEGGYYARGARAAVWENFKDNPLFDISTEHPTTYYEDMQRAIFCLCPLGWAPWSPRLVEAVIFGCIPVIIADDI---VLPFADAIP 320
Potri012G109600 LQRATLVQTFGQRN-HVCLKDGSITVPPYAP--PQKMQTHLIPEKTPRSIFVYFRGLFYDVGNDPEGGYYARGARAAVWENFKDNPLFDISTEHPATYYEDMQRAVFCLCPLGWAPWSPRLVEAVIFGCIPVIIADDI---VLPFADAIP 322
Potri003G162000 LRRATLVQTFGQRN-HVCLNEGSITIPPYAP--PQKMQAHQIPLDTPRSIFVYFRGLFYDVNNDPEGGYYARGARAAVWENFKNNPLFDISTDHPTTYYEDMQRAIFCLCPLGWAPWSPRLVEAVVFGCIPVIIADDI---VLPFADAIP 318
Potri001G068100 LQRSTLVQTFGQRN-HVCLNEGSITIPPFAP--PQKMQAHQIPPDIPRSIFVYFRGLFYDVNNDPEGGYYARGARAAVWENFKNNPLFDISTDHPTTYYEDMQRAIFCLCPLGWAPWSPRLVEAVVFGCIPVIIADDI---VLPFADAIP 318
FRA8 LRNSIILQTFGVTFNHPCQEVENVVIPPYISPESLHKTQKNIPVTKERDIWVFFRGKMELHPKNISGRFYSKRVRTNIWRSYGGDRRFYLQRQRFAGYQSEIARSVFCLCPLGWAPWSPRLVESVALGCVPVIIADGIR---LPFPSTVR 370
XGD1 CN-ANTSEGFRPNV---------DVSIPEIYLPKGKLGPSFLGKSPRVRSILAFFAGRSHG---------EIRKILFQHWKEMDNEVQVYDRLPPGKDYTKTMGMSKFCLCPSGWEVASPREVEAIYAGCVPVIISDNY---SLPFSDVLN 428
Os10g10080 LRRATLVQTFGQKN-HACLKDGSITVPPYTP--AHKIRAHLVPPETPRSIFVYFRGLFYDTSNDPEGGYYARGARASVWENFKNNPMFDISTDHPQTYYEDMQRAVFCLCPLGWAPWSPRLVEAVVFGCIPVIIADDI---VLPFSDAIP 322
Os04g32670 LQRATLVQTFGQEN-HVCLKEGSITIPPYAP--PQKMQAHLIPPDTPRSIFVYFRGLFYDTGNDPEGGYYARGARASLWENFKNNPLFDISTDHPPTYYEDMQRAVFCLCPLGWAPWSPRLVEAVVFGCIPVIIADDI---VLPFADAIP 325
Os01g70200 LQRATLVQTFGQKN-HVCLKDGSITIPPYAP--PQKMQAHLIPPDTPRSIFVYFRGLFYDTSNDPEGGYYARGARASVWENFKNNPLFDISTDHPPTYYEDMQRSVFCLCPLGWAPWSPRLVEAVVFGCIPVIIADDI---VLPFADAIP 322
Os01g70190 LRRATLVQTFGQKD-HVCLKEGSITIPPYAP--PQKMKTHLVPPETPRSIFVYFRGLFYDTANDPEGGYYARGARASVWENFKNNPLFDISTDHPPTYYEDMQRSIFCLCPLGWAPWSPRLVEAVVFGCIPVIIADDI---VLPFADAIP 320
Os01g70180 LRRATLVQTFGQRH-HPCLQPGSITVPPYAD--PRKMEAHRISPATPRSIFVYFRGLFYDMGNDPEGGYYARGARASVWENFKDNPLFDISTEHPATYYEDMQRAIFCLCPLGWAPWSPRLVEAVVFGCIPVIIADDI---VLPFADAIP 327
Os02g32110 LRRATLVQTFGQKN-HVCLKEGSITIPPYAP--PQKMQAHLIPPDTPRSIFVYFRGLFYDNGNDPEGGYYARGARASLWENFKNNPLFDISTEHPATYYEDMQRSVFCLCPLGWAPWSPRLVEAVVFGCIPVIIADDI---VLPFADAIP 338
Bd2g59400 LRRATLVQTFGQKD-HVCLKEGSINIPPYAP--PQKMKTHLVPPETPRSIFVYFRGLFYDTANDPEGGYYARGARASVWENFKNNPLFDISTDHPPTYYEDMQRAIFCLCPLGWAPWSPRLVEAVVFGCIPVIIADDI---VLPFADAIP 316
Bd2g59410 LQRATLVQTFGQKN-HVCLKDGSITIPPYAP--PQKMQNHLIPGDTPRSIFVYFRGLFYDTSNDPEGGYYARGARASVWENFKNNPLFDISTDHPPTYYEDMQRSVFCLCPLGWAPWSPRLVEAVVFGCIPVIIADDI---VLPFADAIP 323
Bd2g59380 LRRATLVQTFGQRN-HVCMQEGSITIPPYAN--PQKMQAHLISPGTPRSIFAYFRGLFYDMGNDPEGGYYARGARASVWENFKDNPLFDMSTEHPSTYYEDMQRAIFCLCPLGWAPWSPRLVEAVVFGCIPVIIADDI---VLPFADAIP 328
Bd5g08400 LQRATLVQTFGQEN-HVCLKEGSIIIPPFAP--PQKMQAHLIPPDTPRSIFVYFRGLFYDTGNDPEGGYYARGARASLWENFKNNPLFDISTEHPATYYEDMQRAVFCLCPLGWAPWSPRLVEAVVFGCIPVIIADDI---VLPFADAIP 334
Bd3g44420 LRHATLVQTFGQKN-HVCLKDGSITIPPYAP--PQKMQAHLIPPDTPRSIFVYFRGLFYDNGNDPEGGYYARGARASLWENFKNNPLFDISTEHPATYYEDMQRSVFCLCPLGWAPWSPRLVEAVVFGCIPVIIADDI---VLPFADAIP 333
Sm442111 LQRATLVQTFGQRY-HVCLKEGSIVVPPYCP--PQKMQAHLIPPSIPRSIFVYFRGLFYDYGNDPEGGYYARGARAAVWENFKDNPLFDISTEHPITYYEDMQRAIFCLCPLGWAPWSPRLVEGVIFGCIPVIIADDI---VLPFADAIP 314
Pp1s7_455V6 LKRATLIQTFGQNH-HVCLKEDSIVIPPYAP--PERMQTRLNPPSTPRSIFAYFRGLFYDPGNDPEGGYYARGARAAIWENFKDNPLFDISTEHPATYYEDMQRAIFCLCPLGWAPWSPRLVEGVIFGCIPVIIADDI---VLPFADAIP 350
.......310.......320.......330.......340.......350.......360.......370.......380.......390.......400.......410.......420.......430.......440.......450
PoIRX10_1 WEEIAIFVAEKNVPILDIILTTVPADAILMKQSLLAEPSLKQAMLYPQPAQPGDALHQILNGLARKLPHGDGVFLKPGEKILNWSVGLSSDLSDMFI------------------------- 438
PoIRX10_3 WEEIAVFVPEKDVPKLDTILTSLPISEILRKQRLLANPSMKQAMLFPQPAQPRDAFHQILNGLARKLPHGDDVFVKPGQTFINWTAGPVGDLKPW------------------------- 416
PoIRX10_4 WKELAVFVAENHVHNLDVILTSIPVHEILWKQSLLANPSVKQALLFSQPAQPGDAFYQILNALARKLPHRNSVFLKPGEKILNWTAGREADLSDMVDILRR------------------------- 425
PoIRX10_2 WEEIAVFVAEKDVTKLDIILTSIPGDEIIKRQSLLANPSLKQAMLYPQPARPGDALHQILNGLARKLPHGDGVFLKPGR---------EDHELVTR------------------------- 374
PoIRX10_5 WEDIGVFVAEKDVPKLDTILTSLPIDEILRKQRLLANPSMKQAMLFPQPAQARDAFHQILNGLARKLPHDRSVYLAPGERILNWTAGPVADLKPW------------------------- 416
PoIRX10_6 WEEIGVFVAEEDVPYLDTILTSIPVEVILRKQRLLANPSMKQAMLFPQPAQSGDAFHQILNGLARKLPHDKSIYLKRGQQVLNWTAGPVGDMKPW------------------------- 410
IRX10 WEEIGVFVAEKDVPELDTILTSIPTEVILRKQRLLANPSMKRAMLFPQPAQPGDAFHQILNGLARKLPHDKSIYLKTGEKALNWTAGPVADLKPW------------------------- 412
IRX10L WEDIGVFVDEKDVPYLDTILTSIPPEVILRKQRLLANPSMKQAMLFPQPAQPGDAFHQVLNGLARKLPHERSVYLRPGEKLLNWTAGPVADLKPW------------------------- 415
Potri012G109600 WEEIGVYVDEEDVPNLDTILTSIPPEVILRKQRLLANPSMKQAMLFPQPAQPGDAFHQVLNGLARKLPHDRSVYLRPSEKILNWTAGPVADLKPW------------------------- 417
Potri003G162000 WEEIGVFVAEEDVPHLDTFLTSIPPEVILRKQRLLANPSMKRAMLFPQPAQPGDAFHQILNGLARKLPHDRTVYLKPGQKVLNWTTGPAGDLKPW------------------------- 413
Potri001G068100 WEEIGVFVAEEDVPNLDTILTSIPPEVILRKQRLLANPSMKRAMLFPQPAQPGDAFHQILNGLARKLPHDRSVYLKSGQNILNWTAGPVGDLKPW------------------------- 413
FRA8 WPDISLTVAERDVGKLGDILEHVAATNLSVIQRNLEDPSVRRALMFNVPSREGDATWQVLEALSKKLNR----SVRRSNSFL------------------------- 448
XGD1 WDSFSIQIPVSRIKEIKTILQSVSLVRYLKMYKRVLE--VKQHFVLNRPAKPYDVMHMMLHSIWLRRLN-----LRLGT------------------------- 500
Os10g10080 WEEIAVFVAEDDVPQLDTILTSIPTEVILRKQAMLAEPSMKQTMLFPQPAEPGDGFHQVMNALARKLPHGRDVFLKPGQKVLNWTEGTREDLKPW------------------------- 417
Os04g32670 WEEIGVFVEEKDVPKLDTILTSMPIDDILRKQRLLANPSMKQAMLFPQPAQPRDAFHQILNGLARKLPHPEGVYLQPSDKRLNWTAGPVGDLKAW------------------------- 420
Os01g70200 WEEIGVFVAEEDVPKLDSILTSIPTDVILRKQRLLANPSMKQAMLFPQPAQAGDAFHQILNGLARKLPHGENVFLKPGERALNWTAGPVGDLKPW------------------------- 417
Os01g70190 WDEIGVFVAEDDVPKLDTILTSIPMDVILRKQRLLANPSMKQAMLFPQPAQPGDAFHQILNGLGRKLPHPKSVYLDPGQKVLNWTQGPVGDLKPW------------------------- 415
Os01g70180 WGEISVFVAEEDVPRLDTILASVPLDEVIRKQRLLASPAMKQAVLFHQPARPGDAFHQILNGLARKLPHPKGVFLEPGEKGIDWDQGLENDLKPW------------------------- 422
Os02g32110 WDEIGVFVDEEDVPRLDSILTSIPIDDILRKQRLLANPSMKQAMLFPQPAQPRDAFHQILNGLARKLPHPDSVYLKPGEKHLNWTAGPVADLKPWK------------------------ 434
Bd2g59400 WDEIGVFVAEDDVPKLDTILTSIPMEVILRKQRLLANPSMKQAMLFPQPAQPGDAFHQILNGLARKLPHNKGVYLTPGQKALNWTQGPKGDLKPW------------------------- 411
Bd2g59410 WEDIGVFVSEDDVPKLDSILTSIPTDVILRKQRLLANPSMKQAMLFPQPAEAGDAFHQILNGLARKLPHGENVFLKPGQKVLNWTAGPVGDLKPW------------------------- 418
Bd2g59380 WDQISVFVPEADVPRLDSILASVPPEDVLRKQRLLASPAMKQAVLFHQPAQPRDAFDQVLNGLARKLPHREGVFLRPGQKVIDWNAGQYDDLKPW------------------------- 423
Bd5g08400 WEEIGVFIEEKDVPKLDTILTSMPIEDILRKQRLLANPSMKQAMLFPQPAQARDAFHQILNGLARKLPHPEGVYLPPGEKHLNWTAGPVGDLKPW------------------------- 429
Bd3g44420 WDEIGVFVDEEDVPKLDSILTSIPIDDILRKQRLLANPSMKKAMLFPQPAQPRDAFHQILNGLARKLPHPESVYLQPGEKHVNWTAGPVGDLKPW------------------------- 428
Sm442111 WEEIGVFVAEKDVPKLDTILTSIPPEVILKKQRLLATPAMKQAMLFPQPAQPGDAFHQILNGLARKLPHDRRIYLNPGEKALNWSSGPPGDLKPCKRNTLDRESRNWHQLLQLLRHWCRVLLF 437
Pp1s7_455V6 WEKIGVFVEEKDVPILDKILCTINHEEVLEKQRLLANPAMKQAMLFPRPAKPGDAFHQILNGLARKLPHDPSIYLQPGQSFLNWTEGPPGDLYPWGNDL------------------------- 449
.......460.......470.......480.......490.......500.......510.......520.......530.......540.......550.......560.......570...
